# A multi-strategy antimicrobial discovery approach reveals new ways to treat Chlamydia

**DOI:** 10.1371/journal.pbio.3003123

**Published:** 2025-04-29

**Authors:** Magnus Ölander, Daniel Rea Vázquez, Karsten Meier, Aakriti Singh, Amanda Silva de Sousa, Fabiola Puértolas-Balint, Milica Milivojevic, Lieke Mooij, Johanna Fredlund, Eduard Calpe Bosch, María Rayón Díaz, Moa Lundgren, Karin van der Wal, Shaochun Zhu, André Mateus, Bjoern O. Schroeder, Jeremy R. Lohman, Barbara S. Sixt

**Affiliations:** 1 Department of Molecular Biology, Umeå University, Umeå, Sweden; 2 The Laboratory for Molecular Infection Medicine Sweden (MIMS), Umeå University, Umeå, Sweden; 3 Umeå Centre for Microbial Research (UCMR), Umeå University, Umeå, Sweden; 4 Department of Biochemistry and Molecular Biology, Michigan State University, East Lansing, Michigan, United States of America; 5 Department of Chemistry, Umeå University, Umeå, Sweden; Universität zu Köln, GERMANY

## Abstract

While the excessive use of broad-spectrum antibiotics is a major driver of the global antibiotic resistance crisis, more selective therapies remain unavailable for the majority of bacterial pathogens. This includes the obligate intracellular bacterial pathogens of the genus *Chlamydia*, which cause millions of urogenital, ocular, and respiratory infections each year. Conducting a comprehensive search of the chemical space for novel antichlamydial activities, we identified over 60 compounds that are chemically diverse, structurally distinct from known antibiotics, non-toxic to human cells, and highly potent in preventing the growth of *Chlamydia trachomatis* in cell cultures. Some blocked *C. trachomatis* development reversibly, while others eradicated both established and persistent infections in a bactericidal manner. The top molecules displayed compelling selectivity, yet broad activity against diverse *Chlamydia* strains and species, including both urogenital and ocular serovars of *C.* *trachomatis*, as well as *Chlamydia muridarum* and *Chlamydia caviae*. Some compounds also displayed synergies with clinically used antibiotics. Critically, we found the most potent antichlamydial compound to inhibit fatty acid biosynthesis via covalent binding to the active site of *Chlamydia* FabH, identifying a new mechanism of FabH inhibition and highlighting a possible way to selectively treat *Chlamydia* infections.

## Abstract 2

Bacteria of the genus Chlamydia cause millions of infections each year and have an obligate intracellular lifestyle that makes finding specific treatments particularly difficult. This study developed a discovery platform that identified over sixty new potent antichlamydial compounds, including one that selectively inhibits bacterial fatty acid synthesis.

## Introduction

Antimicrobial resistance is a major societal challenge that is quickly aggravating and is estimated to cause over 10 million deaths annually by 2050 [1]. A prime driver of this alarming development is the heavy use of broad-spectrum antibiotics that select for resistance not only in the targeted pathogen but in a wide range of exposed microbes [2]. Critically, by perturbing commensal and environmental microbial communities, these broad-acting drugs can also damage human health and ecosystems [3,4]. Clearly, there is an urgent need to develop more selective and sustainable treatment alternatives, especially for those pathogens that account for significant global consumption of antibiotics.

A prime example is *Chlamydia trachomatis*, the world’s most common bacterial agent of sexually transmitted infections (STIs), responsible for over 130 million cases annually [5]. *Chlamydia* STIs can cause serious complications and long-term sequelae, such as pelvic inflammatory disease, infertility, and pregnancy complications, and are considered a risk factor for cervical and ovarian cancers [6–8]. *C. trachomatis* also causes trachoma, a devastating blinding ocular disease [9]. Moreover, closely related *Chlamydia* spp. have significant medical and veterinary impact as well [10,11].

Lacking both effective vaccines and selective therapeutics, the current clinical management of *C. trachomatis* relies on the use of broad-acting antibiotics [12]. Given that in the context of STIs this must include treatment of sexual contacts [6], and that mass treatment of entire communities remains common practice in managing trachoma [13], this sums up to substantial global antibiotic use for the control of this single pathogen alone. Even worse, the clinically most commonly used antibiotics, doxycycline and azithromycin [12], are especially disruptive to the human gut microbiota [14], and their use in anti-*Chlamydia* therapy indeed exacerbates resistance development in bystander pathogens [15–17]. At the same time, treatment failure rates of up to 5%–23% have been reported for *Chlamydia* STIs [18].

Critically, developing therapeutics that can target *Chlamydia* spp. selectively appears feasible when considering the unique biology of these bacteria, which form a phylogenetically distinct group of microbes, with all members sharing a strictly host-associated and obligate intracellular lifestyle. Within their host cells, the bacteria thrive inside membrane-enclosed vacuoles, called inclusions, engage in highly specific host-pathogen interactions, and undergo complex developmental transitions [19]. More specifically, the infectious form of the bacteria, the elementary body (EB), invades a host cell and then differentiates into the reticulate body (RB), the replicative form, which multiplies within the inclusion. Eventually, RBs differentiate back into EBs, which are released by host cell lysis or extrusion of the inclusion about 48–72 h post-infection (hpi) enabling spread of infection to other cells [20]. Given their adaptation to the intracellular niche, *Chlamydia* spp. have highly reduced metabolic capacities and therefore depend on numerous metabolites provided by their host cells [21]. Overall, this distinct biology and strong dependence on host cells should present ample opportunities for targeting the pathogen’s unique traits or virulence mechanisms selectively without causing collateral damage. Yet, the development of such narrow-spectrum therapeutics has not yet been achieved, in part because our mechanistic understanding of targetable *Chlamydia*–host interactions remains poor despite recent advances empowered by emerging tools enabling molecular genetic manipulation of *C. trachomatis* [22].

To identify molecular targets suitable for the development of selective antichlamydials, as well as chemical scaffolds acting on those, we here chose to apply an initially target-agnostic approach, which combined both experimental and virtual screening of large libraries of small drug-like molecules, followed later by an in-depth analysis of candidate molecules by determinations of compound potency, toxicity, selectivity, interactions, and mode of action (MoA). As a result, we here report the identification of numerous potent selective antichlamydials that are chemically distinct from currently known antibiotics. We further determined the most potent selective antichlamydial identified to be a covalent inhibitor of *Chlamydia* FabH, highlighting the unique features of this enzyme initiating fatty acid biosynthesis to enable selective therapeutic targeting.

## Results

### Development of a screening assay for novel chemical inhibitors of *C. trachomatis* growth

To efficiently screen for antichlamydial compounds, we developed a screening assay that operates in a 384-well plate format, requires minimal manual handling of plates, and can monitor, in parallel, inhibition of intracellular bacterial growth and compound toxicity towards host cells. Specifically, we used a Green Fluorescent Protein (GFP)-expressing strain of *C. trachomatis* L2/434/Bu (CTL2-GFP) to infer bacterial growth from bulk GFP fluorescence, and we used the metabolic conversion of resazurin into its product resorufin to infer host cell viability from bulk resorufin fluorescence (S1A Fig). In brief, human cervical epithelial (HeLa) cells were infected with CTL2-GFP in suspension and seeded into plates containing compounds in dimethyl sulfoxide (DMSO). After 26 h of incubation, resazurin was added, followed by further incubation for 2.5 h, and measurement of GFP and resorufin fluorescence. Wells treated with ciprofloxacin or staurosporine served as positive controls for bacterial growth inhibition and cytotoxicity, respectively, while wells treated solely with DMSO (solvent) served as negative control.

During assay optimization, we found a seeding density of 4,000 cells per well and an infection dose of 30 inclusion-forming units (IFUs) per cell to be ideal, as this gave near-maximal resorufin fluorescence without signal saturation (S1B Fig and S1A Data) and high GFP fluorescence without cytotoxicity (S1C–S1E Fig and S1B and S1C Data). Importantly, we found that CTL2-GFP and HeLa cells showed reasonable tolerance to DMSO, up to concentrations of around 0.4% and 1.6%, respectively (S1F Fig and S1D Data). Moreover, in an interleaved-signal plate layout [23], both readouts resulted in data that were robust and uniform throughout the plate, with *Z*′ factors of 0.53–0.62 and 0.51–0.73 for bacterial growth inhibition and toxicity, respectively (S1G and S1H Fig and S1E and S1F Data).

We benchmarked our assay using a set of traditional antibiotics, all of which gave dose-dependent reductions in GFP fluorescence with minimal effects on host cell viability (S2A Fig and S1G Data). Minimum inhibitory concentrations (MICs) calculated from these data spanned more than five orders of magnitude and were, with one exception, within or close to the ranges reported [24–34] (S2B Fig and S1H Data).

Taken together, we developed an assay with excellent performance parameters that can reliably measure the inhibition of *C. trachomatis* growth over a wide range of compound concentrations.

### Identification of novel antichlamydials through experimental compound library screening

**Fig 1:**
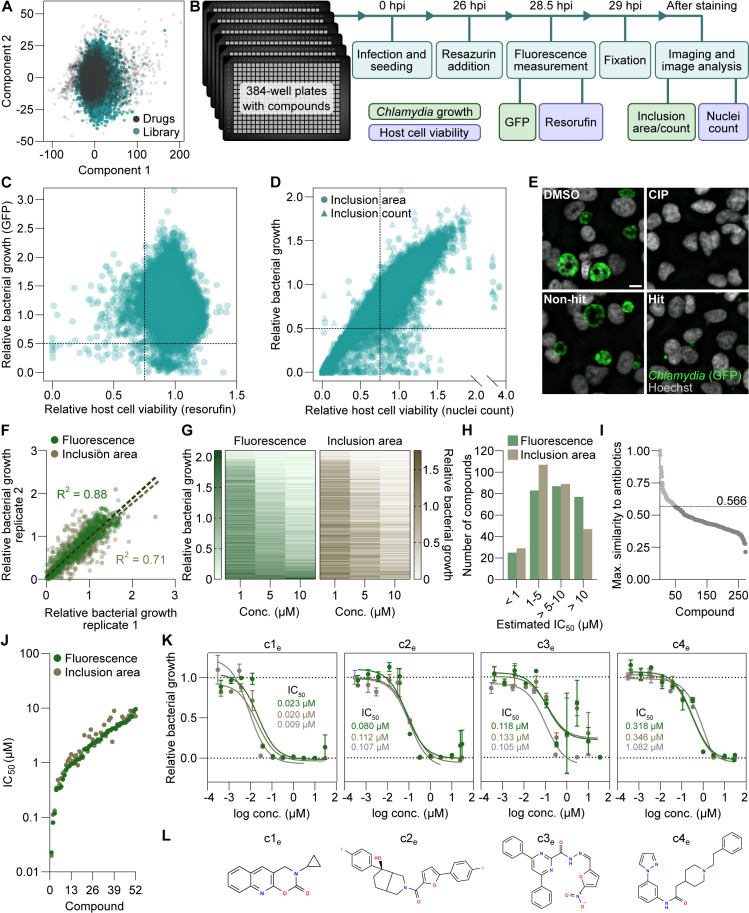
Identification of novel antichlamydials through experimental compound library screening. **(A)** Chemical diversity of the screening library of 36,785 compounds, illustrated by principal component analysis based on molecular descriptors calculated with PaDEL-Descriptor [35]. “Drugs” denotes 6,798 compounds from the Drug Repurposing Hub [36], included to represent the chemical space of pharmaceutical drugs. **(B)** Screening protocol, highlighting major experimental steps, time points, and measurements. Compounds were tested at 10 µM. **(C)** Bulk fluorescence measurements of GFP and resorufin, representing *C.* *trachomatis* growth and host cell viability, respectively. The dashed lines indicate the cut-offs used for hit selection. **(D)** Image-based measurements of inclusion area and count and host cell nuclei count, representing *C. trachomatis* growth and host cell viability, respectively. The dashed lines indicate the cut-offs used for hit selection. **(E)** Representative image examples of a non-hit and a hit, as well as positive (ciprofloxacin, CIP) and negative (DMSO vehicle) controls for bacterial growth inhibition. Scale bar is 10 µm. **(F)** The 271 hits from the screen retested in duplicate with the screening assay protocol at 1, 5, and 10 µM, highlighting the overall consistency between the two replicates. **(G)** Bacterial growth inhibition achieved by the 271 hit compounds, shown at the single-compound level. The compounds are sorted by mean bulk fluorescence across the three concentrations. **(H)** Estimated IC_50_ of the 271 hits. **(I)** Maximal structural similarity, represented by Tanimoto coefficients, between the hits and 506 known antibiotics in the Drug Repurposing Hub [36]. The dashed line indicates the maximum value among the compounds prioritized. **(J)** Potency of the 52 priority compounds, determined with the screening assay protocol. **(K)** Dose–response curves of the four most potent compounds identified by experimental screening (c1_e_–c4_e_) (mean ± SD, *n* = 3). Lines (green, GFP fluorescence; brown, inclusion area; gray, bacterial genome copies) indicate curve fits used for IC_50_ calculation. **(L)** Chemical structures of the four most potent compounds, drawn based on their SMILES strings using OpenBabel (version 3.0.0) [37]. The data underlying this figure can be found in S2 Data.

We screened a library of 36,785 small molecules displaying drug-like properties and covering a wide chemical space (Fig 1A and S2A Data). In this process, we also complemented the assay described above with an imaging-based readout (Fig 1B). After measurements of bulk GFP and resorufin fluorescence, cells were fixed, stained with the DNA dye Hoechst, and imaged at a high-content imaging platform to detect chlamydial inclusions (GFP) and host cell nuclei (Hoechst). The total area and number of inclusions in individual wells and the number of nuclei then served as additional proxies for chlamydial growth and host cell viability.

When analyzing the data derived from bulk fluorescence measurements in the screen, we identified 303 compounds that at 10 µM displayed antichlamydial activity (>50% reduction in GFP fluorescence), but were non-toxic toward the host cells (<25% reduction in resorufin fluorescence) (Fig 1C and S2B Data). Using the same cut-offs in the analysis of image-derived data, we identified 136 compounds as non-toxic antichlamydials according to the number of nuclei and either total inclusion area or inclusion count (Fig 1D and 1E and S2B Data). We then generated a decision tree to integrate data from both fluorescence measurements and imaging for a final classification into hits and non-hits and thereby selected a total of 271 promising compounds for further evaluation (S3 Fig and S2B Data).

A retesting of the 271 compounds in duplicate at three concentrations confirmed 179 compounds to be antichlamydial and non-toxic (>50% reduction in GFP fluorescence and inclusion area, and <25% reduction in resorufin fluorescence, at ≤10 µM; Fig 1F and 1G and S2C Data). We further estimated compound potencies, using as measure the half-maximal inhibitory concentration (IC_50_), considered less ambiguous than MICs [38] (Fig 1H and S2C and S2D Data), and decided to prioritize compounds with estimated IC_50_ ≤ 5 µM (according to both GFP fluorescence and inclusion area) for further follow-up. Moreover, because our objective was to discover antichlamydials with novel chemical structures, not yet associated with antimicrobial activities, we excluded compounds that resembled known antibiotics (Fig 1I and S2C and S2E Data), resulting in a list of 52 priority compounds (S4 Fig and S2C Data).

Subsequently, we retested these 52 compounds in triplicate at a wide range of concentrations to enable more accurate potency determinations (S5 Fig and S2F Data). Ten compounds showed submicromolar IC_50_ values for both growth parameters (i.e., GFP fluorescence and inclusion area) (Fig 1J and S2F Data). Three were particularly potent, with IC_50_ values around or below 0.1 µM, while the next had an IC_50_ value around 0.3 µM (Fig 1K and 1L and S2F Data). The antichlamydial efficacy of these four most potent compounds was also validated by an orthogonal assay based on the detection of bacterial genome copies by quantitative PCR (Fig 1K and S2G Data).

Altogether, our efforts led to the identification of a set of highly potent non-toxic antichlamydial compounds that are structurally different from known antibiotics.

### Predictive modeling enabling antichlamydial discovery through virtual screening

**Fig 2:**
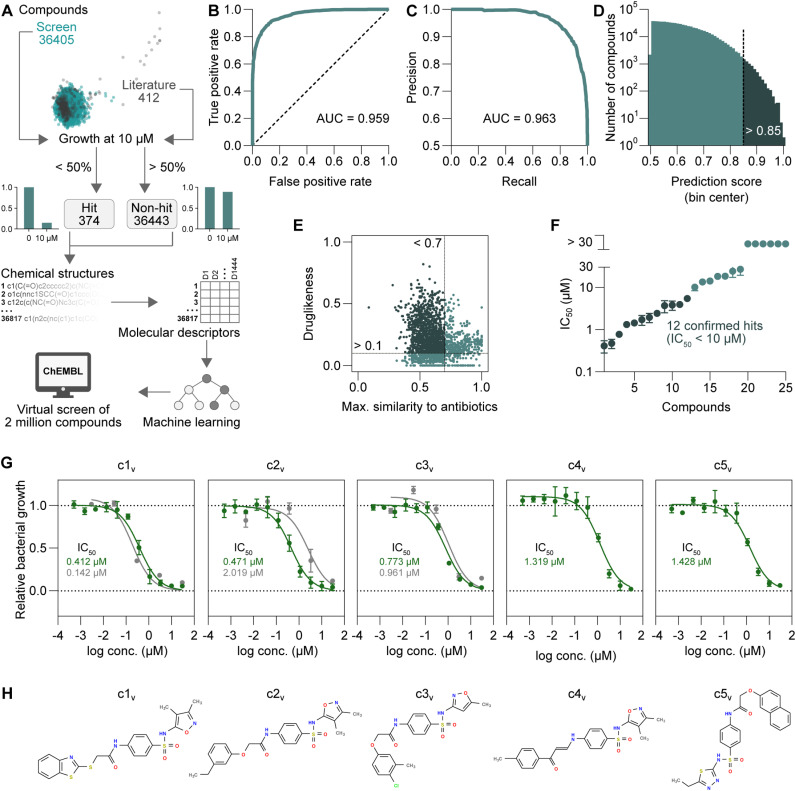
Predictive modeling enabling antichlamydial discovery through virtual screening. **(A)** Schematic illustration of model development. **(B, C)** An area under the receiver operating characteristic curve (ROC-AUC) (B) and an area under the precision-recall curve (PRC-AUC) (C) plot showing the performance of the model after training. **(D)** Histogram of predicted antichlamydial hits from a virtual screen of the ChEMBL database. The dashed line indicates the cut-off used to filter the hits based on prediction scores. **(E)** Maximal structural similarity to known antibiotics (Tanimoto coefficients) and quantitative estimate of drug-likeness (QED) of the filtered hits. The dashed lines indicate the cut-offs used for further filtering. **(F)** Potency of 25 compounds subjected to experimental testing, as measured by bulk GFP fluorescence using the screening assay protocol (mean ± SD, *n* = 3). **(G)** Dose–response curves of the five most potent compounds identified by virtual screening (c1_v_–c5_v_) (mean ± SD, *n* = 3). Lines (green, GFP fluorescence; gray, bacterial genome copies) indicate curve fits used for IC_50_ calculation. **(H)** Chemical structures of the five most potent compounds, drawn based on their SMILES strings using OpenBabel (version 3.0.0) [37]. The data underlying this figure can be found in S3 Data.

As a complement to our experimental approach, we took advantage of our data to develop a simple machine-learning-based prediction model, which then enabled us to identify additional compounds with potential antichlamydial activity through virtual screening (Fig 2A).

The model was based on our own dataset (including all 36,405 compounds from the experimentally screened library for which structures were available) and 412 additional compounds previously reported to have been tested for antichlamydial activity (S3A Data). All molecules in this expanded dataset were classified as hits or non-hits using the same criterion as used in our experimental screen (>50% growth inhibition at 10 µM). Using the PaDEL-Descriptor tool [35], we then calculated 1,444 1D and 2D molecular descriptors for all compounds. Subsequently, these were used as input to train a random forest classifier, which, given a new structure, predicts a molecule’s probability of anti-chlamydial activity based on the chemical information contained in the training dataset. The model achieved an area under the receiver operating characteristic curve (ROC-AUC) of 0.959 and an area under the precision-recall curve (PRC-AUC) of 0.963 on the training data (Fig 2B and 2C and S3B and S3C Data), with 88.9% correctly classified instances (based on 10-fold cross-validation).

Next, we applied our model to perform predictions on the ChEMBL database, which includes over 2 million chemically diverse drug-like compounds [39]. This virtual screen resulted in 5,474 possible antichlamydials with prediction scores >0.85 (Fig 2D and S3D Data). Filtering these to retain only one structural isomer per compound reduced the number to 2,871. We further retained only such that had a low structural similarity to known antibiotics (Tanimoto coefficient < 0.7) and a quantitative estimate of drug-likeness (QED [40]) > 0.1 (Fig 2E and S3E Data), which left 174 compounds (S3F Data). After determining their commercial availability, we selected 25 affordable compounds for experimental validation (S6 Fig). Twelve of these (representing two series of chemical analogs and one more distinct molecule) indeed showed antichlamydial activity with IC_50_ values below 10 µM, with the top three displaying submicromolar IC_50_ values, and the following two values below 1.5 µM (Fig 2F–2H and S3G and S3H Data). The antichlamydial activity of the top three compounds was also confirmed by quantitative PCR (Fig 2G and S3I Data). The 12 validated compounds share a sulfonamide core structure with previously identified antichlamydials included in the training set [41], yet have in part relatively low overall structural similarity (Tanimoto coefficients of around 0.37–0.78) to the previously described antichlamydials (S3F Data). Notably, among the 25 tested molecules, we also observed seven additional molecules to display some though less potent antichlamydial activity with IC_50_ values between 10 µM and 30 µM (Fig 2F and S3G and S3H Data). These molecules also included compounds that lack a sulfonamide core structure (S6 Fig).

Taken together, we present here a predictive model that could successfully identify highly potent antichlamydials through virtual screening of a very large compound database.

### Image analysis-based discovery of persistence-inducing compounds

The integration of an image-based readout in the experimental compound library screen also allowed our data to be searched for compounds inducing specific alterations in inclusion morphology. A prominent example is “chlamydial persistence”, a stress response that is induced by certain antibiotics, in particular beta-lactams, but also other stressors, such as the immune mediator interferon-γ or nutrient shortages, and is characterized by the formation of aberrant bodies (ABs), i.e., highly enlarged RBs [42]. These ABs do not divide nor differentiate into EBs but can survive within their host cell for prolonged periods of time. Upon removal of the stressor, ABs give rise to normal RBs, and chlamydial replication and development can resume [42]. While persistence inducers may not be the preferred starting points for the development of novel clinical drugs, they may serve as tools for deciphering the mechanistic basis of this poorly understood phenomenon that has potential clinical importance in chronic and recurrent infections [42].

An inspection of the image data obtained in the experimental compound library screen indeed identified two compounds (persistence-inducing compounds 1–2, in brief c1_p_–c2_p_) to induce AB formation (S7A and S7B Fig). We used confocal microscopy to study the effects of those compounds on bacterial morphology at higher resolution (S7C and S7D Fig and S4A Data). As expected, treatment with the beta-lactam antibiotic penicillin G caused the appearance of enlarged bacteria (i.e., ABs) with diameters of around 4–8 µm. AB formation was also observed in cells treated with c1_p_ or c2_p_, with mean bacterial diameters of about 2 µm and 4 µm, respectively, well within the reported range of AB sizes [43]. We further observed that persistence induction by c1_p_ and c2_p_ was reversible, as ABs disappeared and bacterial growth resumed, when the compounds were washed out after 28 h of treatment (S7E Fig). Moreover, c1_p_ and c2_p_ blocked the formation of EBs, as quantified by determining the appearance of bacteria capable of initiating a new round of infection, and this blockage was also reversible (S7F Fig and S4B Data). It is noteworthy that the number of EBs recovered 40 h after wash-out of c1_p_ or c2_p_ was about 10-fold lower when compared to EB yields obtained after wash-out of penicillin G. This may suggest a less efficient compound wash-out, a need for longer recovery times, or that c1_p_ and c2_p_ have mixed persistence-inducing and bactericidal activities.

Collectively, these data demonstrate that the image-based aspects of our screening approach can also be used to identify compounds causing more complex morphological phenotypes, typified here by the discovery of two novel persistence inducers. However, our further mechanistic work described below focused on molecules that abrogated bacterial growth without causing morphological signs of persistence.

### Determination of antibacterial selectivity of our best antichlamydials

**Fig 3:**
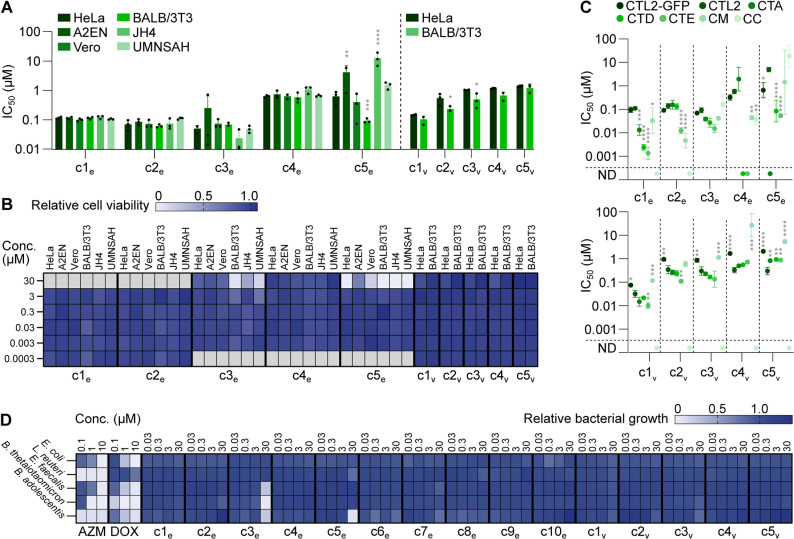
Determination of antibacterial selectivity of our best antichlamydials. **(A)** Potency of selected top compounds in different cell lines infected with CTL2-GFP, as measured by bulk GFP fluorescence (mean ± SD, *n* = 3, two-way ANOVA with Sidak’s multiple comparisons test of HeLa vs. other cell lines). **(B)** Effects of selected top compounds on viability in different cell lines, measured in parallel with (A), using resorufin fluorescence (mean of *n* = 3). Gray boxes indicate concentrations not tested. **(C)** Potency of selected top compounds in HeLa cells infected with the indicated strains, as measured by inclusion area (mean ± SD, *n* = 3, two-way ANOVA with Dunnett’s multiple comparisons test vs. CTL2). ND, not determinable from the obtained curve fits, due to low or inconsistent antichlamydial activity. **(D)** Effect of top compounds on the growth of five gut microbiota species in liquid medium, monitored by OD_600_ measurements (mean of *n* = 3). The data underlying this figure can be found in S5 Data.

To verify that the antichlamydial activities described above were not restricted to HeLa cells, we selected top compounds from the experimental (c1_e_–c5_e_) and virtual (c1_v_–c5_v_) screens and tested them against CTL2-GFP in cells of different origins. Beside HeLa cells, we chose A2EN cells, as they are human endocervical epithelial cells derived from non-cancerous tissue [44]. Moreover, we included cells from animal species, i.e., monkey (Vero), mouse (BALB/3T3), guinea pig (JH4), and chicken (UMNSAH), that may in part serve as hosts in future in vivo testing. Overall, we observed IC_50_ values to be similar across all cell lines for c1_e_, c2_e_, and c4_e_, while c3_e_ and c5_e_ showed some variability (Figs 3A and S8A and S5A and S5B Data). Compounds c1_v_–c5_v_ displayed similar potency in HeLa and BALB/3T3 cells (Figs 3A and S8B and S5A and S5B Data). Critically, c1_e_, c2_e_, c4_e_, and c1_v_–c5_v_ were non-toxic up to the highest tested concentration (3 µM or 30 µM; Figs 3B and S9A and S9B and S5C Data). While c3_e_ and c5_e_ were toxic to some cell lines at 30 µM, both were well tolerated at lower potently antichlamydial concentrations.

Next, we evaluated the effect of c1_e_–c5_e_ and c1_v_–c5_v_ on other strains and species of *Chlamydia*. IC_50_ values for c1_e_–c4_e_ and c1_v_–c3_v_ differed by less than 3-fold between CTL2-GFP and parental CTL2, and those seen for c5_e_ and c4_v_–c5_v_ by less than 10-fold (Fig 3C and S5D Data). All compounds were active against the trachoma serovar, *C. trachomatis* A/HAR-13 (CTA), yet c5_e_ was less potent, precluding a calculation of its IC_50_. Moreover, c1_e_–c3_e_, c5_e_, and c1_v_–c5_v_ showed similar or higher potency against the genital serovars, *C. trachomatis* D/UW-3/Cx (CTD) and *C. trachomatis* E/Bour (CTE), than against CTL2, while c4_e_ was less active against these strains. Compounds c1_e_–c5_e_ and c2_v_ also showed similar or higher potency against *Chlamydia muridarum* (CM) than against CTL2, while compounds c1_v_ and c3_v_–c5_v_ were less potent against this species. Results for *Chlamydia caviae* (CC) were variable with c3_e_–c5_e_ being active and c1_e_–c2_e_ and c1_v_–c5_v_ less potent, precluding calculation of their IC_50_.

As our objective was to discover compounds that inhibit *Chlamydia* selectively but spare commensal bacteria, we further tested 15 of our top compounds (c1_e_–c10_e_ and c1_v_–c5_v_) against five bacterial species, *Escherichia coli*, *Lactobacillus reuteri*, *Enterococcus faecalis*, *Bacteroides thetaiotaomicron*, and *Bifidobacterium adolescentis*, representing the four dominating phyla of the human gut microbiota [45]. In general, the compounds did not affect the growth of the bacteria in liquid media at up to 30 µM (Figs 3D and S10A and S5E and S5F Data). Exceptions were c3_e_ and c5_e_, which at 30 µM were inhibitory to some species. In stark contrast, azithromycin fully inhibited the growth of all tested species at 10 µM or lower, and doxycycline fully inhibited all but *L. reuteri* (Fig 3D and S5E Data), already previously reported to resist the action of this drug [46]. Of note, c1_e_–c3_e_ had little or no effect on the growth of an even larger set of species of gut bacteria, also including *Blautia coccoides*, *Prevotella copri*, *Prevotella histicola, Bacteroides fragilis,* and *Dubosiella newyorkensis*, when tested in radial diffusion assays, in which the bacteria were exposed to compounds for 24–48 h before measurements of growth inhibition zones (S10B Fig and S5G Data). Moreover, selected compounds (c1_e_–c4_e_ and c1_v_–c2_v_) were also tested for growth-inhibitory effects against key species of the vaginal microbiota [47], including *Lactobacillus crispatus*, *Lactobacillus iners*, *Gardnerella vaginalis, and Candida albicans*, and were found in liquid media to be non-inhibitory at concentrations up to 30 µM, with the exception of c3_e_, which partially inhibited *L. iners* and *G. vaginalis* at 30 µM (S11 Fig and S5F and S5H Data).

Taken together, the majority of our top compounds are non-toxic, potent, and selective inhibitors of the intracellular growth of *Chlamydia* spp. and do not, in general, affect commensal bacteria of the human microbiota.

### Determination of between-compound interactions and interactions with clinical antibiotics

Next, we set out to explore the potential for synergistic interactions among our top compounds and between them and antibiotics currently used in the clinics to treat *Chlamydia* or other infectious diseases. From a clinical perspective, synergistic interactions could be leveraged in combination therapies, allowing multitarget engagement and a reduction in compound dose, thereby lowering the risk for side effects and resistance development [48]. Moreover, interaction profiles can inform about the compound MoA [49].

**Fig 4:**
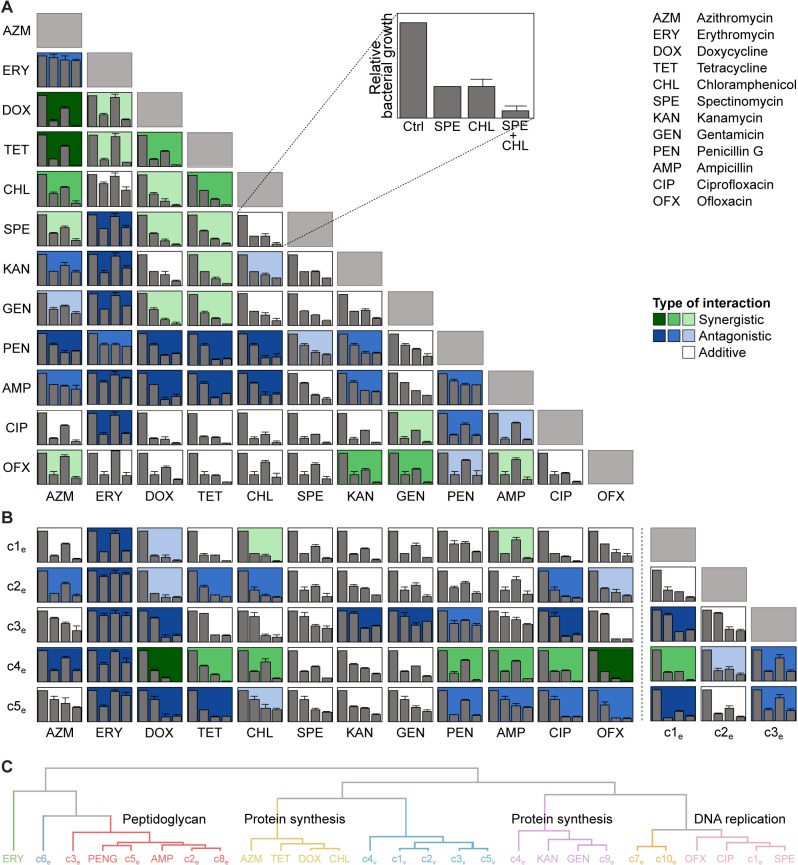
Determination of between-compound interactions and interactions with clinical antibiotics. **(A)** Bacterial growth inhibition in HeLa cells infected with CTL2-GFP and classification of interaction type for all pairwise combinations of 12 clinical antibiotics, tested at IC_50_ (mean ± SD, *n* = 3). Interaction type was classified based on calculations of epistasis, as previously described [49]. Darker colors indicate stronger synergistic or antagonistic interactions. **(B)** Bacterial growth inhibition and classification of interaction type for pairwise combinations of selected compounds and antibiotics, tested at IC_50_ as in (A) (mean ± SD, *n* = 3). **(C)** Hierarchical clustering based on principal component analysis of pairwise interaction profiles (first three principal components), using the calculated epistasis values. The naming of the clusters is based on the targets of the known antibiotics they contain. The data underlying this figure can be found in S6 Data.

To establish a framework for testing combination treatments, we tested 12 clinical antibiotics in all possible pairwise combinations against CTL2-GFP in HeLa cells. Among the 66 combinations, we observed instances of all interaction types (synergistic, antagonistic, and additive) (Fig 4A and S6A Data). We then tested our best compounds (c1_e_–c10_e_ and c1_v_–c5_v_) in combination with the 12 antibiotics and found that c4_e_ and c9_e_ showed strong or moderate synergistic interactions with some of the antibiotics (Figs 4B and S12 and S6B Data). Most prominently, c4_e_ synergized strongly with doxycycline and ofloxacin. Finally, we tested c1_e_–c3_e_ in pairwise combinations with c1_e_–c10_e_ and c1_v_–c5_v_ and observed relatively strong synergistic interactions between c1_e_ and c4_e_ and between c2_e_ and c6_e_ (Figs 4B and S12 and S6C Data).

The kind of overall pairwise interaction profiles generated here have previously been used to cluster antibiotics by MoA and to identify compounds with novel mechanisms, for instance in *E. coli* [49]. Thus, we used hierarchical clustering based on principal component analysis to compare our interaction profiles (Fig 4C). Antibiotics with similar MoA indeed formed clusters. For instance, the ribosome-targeting antibiotics azithromycin, chloramphenicol, doxycycline, and tetracycline formed one cluster, while the peptidoglycan-targeting antibiotics ampicillin and penicillin G formed another (Fig 4C). While the precision with which this approach can predict the MoA of a novel antichlamydial compound may be low at the current stage, as the analysis included only a few known MoAs and no molecules having selective activity against *Chlamydia*, we did notice that c2_e_, c3_e_, c5_e_, and c8_e_ cluster together with the peptidoglycan-targeting antibiotics. Moreover, c1_e_ clustered with the fluoroquinolones ciprofloxacin and ofloxacin, which interfere with bacterial DNA replication, while compounds c4_e_ and c9_e_ clustered with the aminoglycosides gentamicin and kanamycin, which disturb proofreading during translation. Unsurprisingly, considering their structural similarities, c1_v_-c5_v_ clustered tightly together, suggesting that they act in a similar way.

Overall, these interaction data provide first hints towards possible MoAs of selected compounds and highlight synergistic interactions that may in the future be leveraged in potential combination therapies.

### Determining the stage in *Chlamydia* development targeted by our best antichlamydials

**Fig 5:**
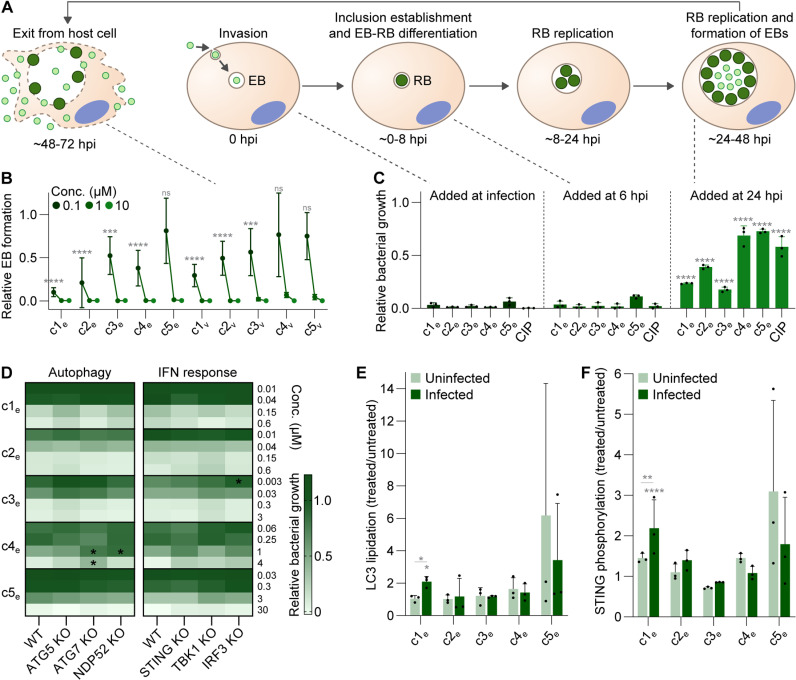
Determining the stage in *Chlamydia* development targeted by our best antichlamydials. **(A)** Illustration of the chlamydial developmental cycle. **(B)** Effect of top compounds on EB formation in HeLa cells infected with CTL2-GFP (mean ± SD, *n* = 3, two-way ANOVA with Dunnett’s multiple comparisons test vs. untreated, **** also for all unmarked; ns, not significant). **(C)** Growth inhibition after addition of top compounds (10 µM) at different points in the developmental cycle, tested in HeLa cells infected with CTL2-GFP, as measured at 48 hpi by bulk GFP fluorescence (mean ± SD, *n* = 3, two-way ANOVA with Dunnett’s multiple comparisons test vs. added at infection). **(D)** Antichlamydial activity of our best compounds in A2EN cells (wild-type (WT) or mutant as indicated) infected with CTL2-GFP, as measured at 28.5 hpi by bulk GFP fluorescence (mean of *n* = 3, two-way ANOVA with Dunnett’s multiple comparisons test). **(E, F)** LC3 lipidation (E) and STING phosphorylation (F) after compound exposure in uninfected A2EN cells and cells infected with CTL2, represented as the ratio of compound-treated and DMSO-control samples (mean ± SD, *n* = 3, two-way ANOVA with Tukey’s multiple comparisons test, vs. DMSO-control if not indicated otherwise, c5_e_ excluded from analysis). CIP, ciprofloxacin. The data underlying this figure can be found in S7 Data.

A key event in the chlamydial developmental cycle is the generation of EBs that can exit the host cell to spread the infection (Fig 5A). Because our compounds can prevent intracellular replication of *C. trachomatis* when present throughout infection, and EB formation is a late event that occurs once RBs have ceased to replicate, we were not surprised to see that c1_e_–c5_e_ and c1_v_–c5_v_ abrogated EB formation by CTL2-GFP in HeLa cells (Fig 5B and S7A Data). To further investigate which stage of intracellular growth was interfered with, we added selected compounds (c1_e_–c5_e_) at a later time point and then assessed bacterial growth at 48 hpi, based on bulk GFP fluorescence. Compound addition at 6 hpi (after invasion and early inclusion formation) resulted in essentially identical growth inhibition compared to addition at the time of infection (Fig 5C and S7B Data). As expected, addition at 24 hpi (to an established infection) reduced bacterial growth less strongly, though most compounds still caused significant levels of inhibition. Notably, c1_e_–c3_e_ reduced growth still by around 60%–80% (Fig 5C and S7B Data), clearly demonstrating that they act on the replicative phase of the infection.

In the replicative phase, the ability of *C. trachomatis* to thrive intracellularly strongly depends on its ability to evade the intrinsic defenses of its host cell [50]. Hence, we reasoned that some of our antichlamydials may act by boosting the defenses or by weakening bacterial evasion. As a first foray into this question, we tested the activity of c1_e_–c5_e_ in A2EN cells deficient for key proteins of either the autophagic machinery (ATG5, ATG7, NDP52) or the STING pathway of the type I interferon response (STING, TBK1, IRF3) (S13 Fig). In most cases, we observed no significant differences between wild-type and defense-defective cells (Fig 5D and S7C Data). However, c4_e_ was less effective in cells deficient for ATG7 or NDP52 (observed as a trend also for ATG5), suggesting that a functional autophagy machinery may be necessary for its full antichlamydial activity. We further analyzed the induction of the defense programs in treated cultures by measuring LC3 lipidation and STING phosphorylation (Fig 5E and 5F and S7D Data). We observed that c5_e_ displayed a tendency to induce both, but highly variably and in infected as well as in uninfected cells, likely connected to its higher toxic potential (Fig 3B and S5C Data). We further noticed a trend for an infection-specific induction of both programs by c1_e_. Interestingly, c4_e_ did not induce overall enhanced levels of LC3 lipidation.

In conclusion, the top antichlamydials identified in this study affect the bacteria in their replicative RB phase. As a consequence, they also abrogate the formation of EBs and can thus stall the spread of infection. Moreover, we found that autophagy may be involved in the action of some of the compounds.

### Compounds can eradicate both established and persistent infections in a bactericidal manner

**Fig 6:**
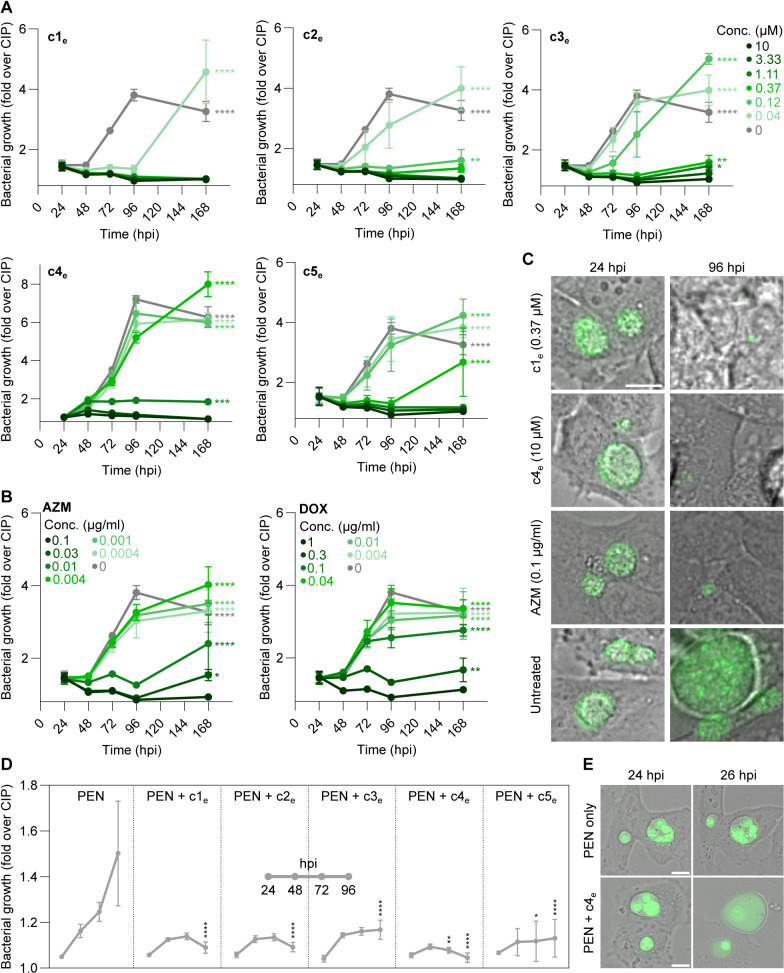
Compounds can eradicate both established and persistent infections in a bactericidal manner. **(A, B)** Time course of bacterial growth in HeLa cells infected with CTL2-GFP and treated with different concentrations of c1_e_-c5_e_ (A) or azithromycin or doxycycline (B) (mean ± SD, *n* = 3). Compounds and antibiotics were first added at 24 hpi, then replenished daily until wash-off at 96 hpi. The data was normalized to ciprofloxacin-treated wells, thus showing fold-increase of GFP fluorescence above background levels (two-way ANOVA with Dunnett’s multiple comparisons test of 168 h vs. ciprofloxacin-treated control). **(C)** Images corresponding to selected time points and concentrations in (A) and (B). Scale bar is 10 µm. **(D)** Time course of bacterial growth in HeLa cells infected with CTL2-GFP in the presence of penicillin G (100 U/ml; present throughout the experiment) and treated with c1_e_–c5_e_ (c1_e_, 0.15 µM; c2_e_–c3_e_, 0.13 µM; c4_e_, 1.74 µM; c5_e_, 0.71 µM) from 24 hpi with daily replenishment (mean ± SD, *n* = 3, two-way ANOVA with Dunnett’s multiple comparisons test of compound vs. penicillin G at each time point). **(E)** Live imaging of cells treated as in (D). Scale bar is 10 µm. AZM, azithromycin; DOX, doxycycline; PEN, penicillin G. The data underlying this figure can be found in S8 Data.

Since our best compounds displayed inhibitory effects even when added to established infections (Fig 5C and S7B Data), we wanted to assess whether a longer treatment duration would lead to total eradication. Thus, we infected HeLa cells with CTL2-GFP, added compounds (c1_e_–c5_e_) at 24 hpi, and replenished them at 48 and 72 hpi, followed by wash-off at 96 hpi and incubation until 168 hpi. Bacterial growth was assessed every 24 h, based on bulk GFP fluorescence. At sufficient concentrations, all compounds acted bactericidal, as they blocked further growth during the course of treatment and did not allow growth to resume after wash-off (Fig 6A and S8A Data). We saw similar trends with azithromycin and doxycycline (Fig 6B and S8A Data), which are known to exert bactericidal effects on *C. trachomatis* [51]. Moreover, fluorescence microscopic imaging supported these findings (Fig 6C).

Next, we wanted to evaluate if our compounds would be effective against persistent infections. Thus, we performed a similar time-course experiment, but adding penicillin G at the time of infection to induce persistence. In cells treated with penicillin G alone, we observed a slow but steady increase in fluorescence throughout the experiment, due to continuous enlargement of ABs. Yet, in the presence of compounds (c1_e_–c5_e_, added at 24 hpi and replenished daily), there was almost no increase, and several compounds reduced fluorescence to close to background by 96 hpi (Fig 6D and S8B Data). Moreover, in live imaging of compound-treated persistent infections, we observed a noticeable effect of c4_e_ as early as 2 h post-treatment. The number of ABs decreased, and inclusions began to fill with diffuse GFP fluorescence, indicating lysis of ABs (Fig 6E).

In summary, these results indicate that our top antichlamydials have bactericidal activity against *C. trachomatis* and can eradicate established as well as persistent infections.

### Identification of *Chlamydia* FabH as molecular target of c1_e_

**Fig 7:**
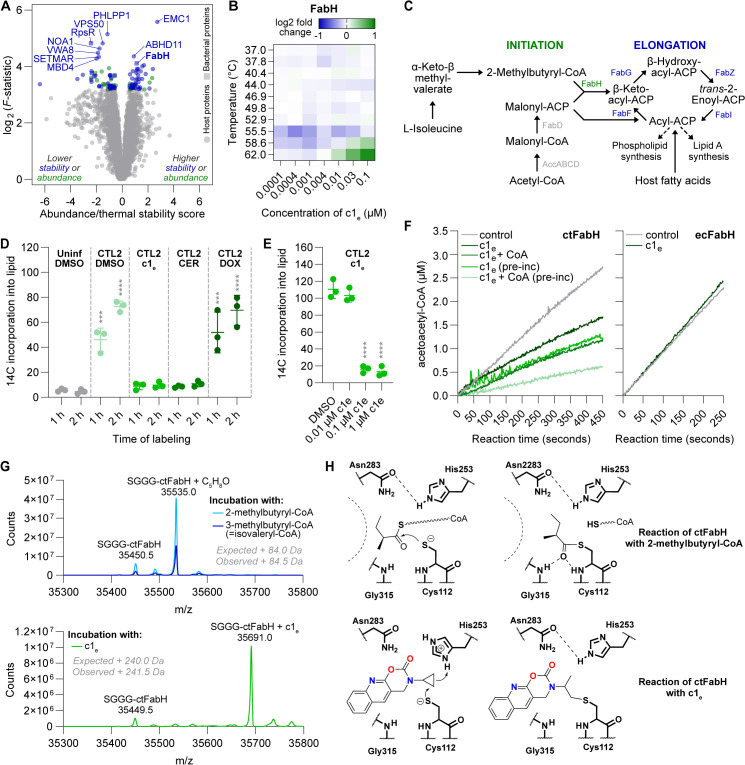
Identification of *Chlamydia* FabH as a molecular target of c1_e_. **(A)** Volcano plot displaying results from the thermal proteome profiling analysis of HeLa cells infected with CTL2 and treated with various concentrations of c1_e_ for 60 min at 24 hpi. Marked in color are the top 100 hits based on log2 of F-statistic. **(B)** Heatmap displaying results from the thermal proteome profiling for FabH, the top stabilized bacterial protein. At each temperature, displayed are fold changes of remaining soluble protein in treated vs. untreated samples. **(C)** Illustration of the branched-chain fatty acid synthesis pathway in CTL2, based on recent literature [52,53,54]. **(D, E)** Inhibition of chlamydial branched-chain fatty acid biosynthesis by c1_e_. HeLa cells infected for 22 h with CTL2 were incubated for 1 or 2 h with l-[U-14C]-isoleucine in presence of the indicated compounds (c1_e_, 1 µM; CER, 50 µM; DOX, 1 µM; DMSO). In (E) cells were incubated for 2 h in presence of the indicated concentrations of c1_e_. 14C incorporation into lipids is displayed as background-subtracted counts per minute (mean ± SD, *n* = 3, two-way ANOVA with Dunnett’s multiple comparisons test vs. uninfected control (D) or DMSO control (E)). **(F)** Enzyme kinetics progress curves of CTL2 FabH (ctFabH, 20 µM) and *Escherichia coli* FabH (ecFabH, 5 µM) with acetyl-CoA (100 µM) and malonyl-CoA substrates (100 µM). Where indicated, reactions were conducted in the presence of c1_e_ (30 µM) and/or CoA (30 µM) with or without preincubation (pre-inc) for 10 min prior to starting the reaction. **(G)** Intact protein mass spectrometry of purified acyl-modification-free ctFabH (10 µM) after incubation with the indicated acyl-CoAs or c1_e_ (50 µM), revealing covalent modification of ctFabH by c1_e_. **(H)** Proposed mechanism of ctFabH acylation by substrate and mode of inhibition by c1_e_ through covalent bond formation with the active site cysteine. CER, cerulenin; DOX, doxycycline. The data underlying this figure can be found in S9 Data.

To better understand the MoA of our most potent compound, c1_e_, we next aimed to identify its molecular target. To this end, we conducted thermal proteome profiling, a technique that can identify proteins that the compound binds and stabilizes against thermal denaturation. In brief, HeLa cells infected for 24 h with CTL2 were treated for 1 h with serial dilutions of c1_e_ and subsequently exposed to a temperature gradient ranging from 37 to 67 °C. The remaining soluble (non-denatured) proteins in each sample were then identified and quantified by mass spectrometry. Among the top three proteins stabilized by c1_e_ in a dose-dependent manner, we found two host proteins, EMC1 and ABHD11, but also CTL2 FabH (3-oxoacyl-[acyl-carrier-protein (ACP)] synthase III), a protein that functions in fatty acid biosynthesis (Fig 7A and 7B and S9A and S9B Data).

To strengthen these findings, we evolved mutant strains of CTL2 resistant to c1_e_’s inhibitory action and then screened them for mutations in *fabH*. To this end, three separately kept lines of CTL2 were passaged in HeLa cells a total of 20 times in the presence of initially low but steadily increasing concentrations of c1_e_. At passage 20, all three evolved mutants could be propagated efficiently at a compound concentration 50 times higher than c1_e_’s initial IC_50_ (S14A Fig and S9C Data). At this concentration, c1_e_ even enhanced the growth of the mutants about 3–5-fold compared to the DMSO control, while it fully inhibited the growth of the wild-type strain (S14B Fig and S9D Data). This suggests that the mutants not only evolved resistance to c1_e_ but also dependence, a phenomenon that has previously been described for several other antibiotic agents [55]. Interestingly, all three mutants shared a point mutation located not within but immediately upstream of *fabH* (S14C Fig), potentially affecting gene expression. Indeed, at the transcriptional level, we observed an about 2-fold increase in *fabH* expression in the mutants relative to the wild-type bacteria (S14D Fig and S9E Data), while a proteome analysis conducted with one of the mutants (mutant 3) indicated that FabH protein levels were increased 5-fold (S14E Fig and S9F Data).

CTL2 FabH (here further referred to as ctFabH) participates in the initiation of fatty acid biosynthesis, using the substrates malonyl-ACP and acetyl-CoA for the synthesis of straight-chain fatty acids, and malonyl-ACP and branched-chain acyl-CoAs (e.g., 2-methylbutyryl-CoA, isovaleryl-CoA, isobutyryl-CoA) for the synthesis of branched-chain fatty acids [52] (Fig 7C). Because human cells do not synthesize branched-chain fatty acids, the activity of ctFabH can be determined by incubating infected cell cultures with a 14C-labeled variant of l-isoleucine, the precursor of 2-methylbutyryl-CoA, and then detecting 14C incorporation into the lipid fraction [56]. Indeed, when we infected cells for 22 h with CTL2 followed by 1 or 2 h of incubation with the labeled amino acid prior to lipid extraction, we observed a robust label incorporation, while hardly any incorporation was observed in the uninfected control (Fig 7D and S9G data). Crucially, when c1_e_ (1 µM) was added together with 14C-labeled l-isoleucine at 22 hpi, incorporation of 14C into lipids was almost completely blocked. The same effect was observed when we instead added cerulenin (50 µM), an established inhibitor of fatty acid elongation, while doxycycline (1 µM), an antibiotic that inhibits bacterial protein synthesis and hence has an entirely different MoA, did not affect 14C incorporation during the 2-h incubation period. Critically, significant inhibition of label incorporation was also observed at 0.1 µM but not 0.01 µM c1_e_ (Fig 7E and S9H Data), correlating with the compound’s antichlamydial activity (Fig 1K and S2F and S2G Data).

To investigate if c1_e_ can inhibit the activity of ctFabH in vitro, we expressed and purified the protein and then studied the inhibitory effects of c1_e_ in a previously established assay that measures the activity of FabH enzymes using acetyl-CoA and malonyl-CoA (instead of malonyl-ACP) as substrates [57]. Kinetic analysis of ctFabH activity revealed the recombinant enzyme to be active (Fig 7F and S9I Data). Critically, c1_e_ displayed inhibitory activity towards ctFabH when added at about equimolar concentration. Yet, consistent with the selective antichlamydial activity of c1_e_, we observed no inhibition of *E. coli* FabH (Fig 7F and S9I Data).

To further assess the action of c1_e_, we conducted mass spectrometric investigations to detect c1_e_ binding to ctFabH. Interestingly, our initial analyses revealed that purified recombinant ctFabH was modified by acyl groups, primarily pentanoyl-groups, brought along from the *E. coli* host in which the protein had been expressed (S15A Fig and S9J Data). We suspected that these acyl chains could potentially interfere with the inhibitory action of c1_e_ by impeding the compound’s binding to ctFabH. Indeed, the addition of CoA, which can serve as an acceptor for the acyl groups, and in particular the pre-incubation of ctFabH with CoA, potentiated the c1_e_-mediated inhibition of ctFabH observed in the in vitro assay (Fig 7F and S9I Data).

Prior to additional mass spectrometric analyses involving recombinant ctFabH, we removed any acyl chains bound by incubating the protein with dithiothreitol at high pH. Subsequently, we observed that incubation of this stripped ctFabH with 2-methylbutyryl-CoA or isovaleryl-CoA, as expected, generated the corresponding acyl-enzyme intermediates, while incubation with c1_e_ generated an FabH-c1_e_ adduct (Fig 7G and S9K Data). Moreover, an initial incubation with 2-methylbutyryl-CoA or isovaleryl-CoA followed by subsequent incubation with c1_e_ prevented c1_e_ binding to FabH (S15B Fig and S9L Data), in line with above-described observation that acylated FabH cannot as efficiently be inhibited by c1_e_. Critically, the prior binding of c1_e_ prevented FabH’s interaction with its substrates (S15B Fig and S9L Data). Further mass spectrometric (MS/MS) analysis of the FabH-c1_e_ adduct revealed the site of c1_e_ attachment to be FabH’s active site cysteine (Figs 7H and S16A–S16C).

As CTL2 has been shown to be able to scavenge host fatty acids for phospholipid synthesis [53], we suspected that a more detailed analysis of the resistant mutants may provide additional information on c1_e_’s MoA. Indeed, whole genome sequencing not only confirmed the presence of the point mutation upstream of *fabH* but also revealed that all three mutants carry additional mutations, notably including such that can be expected to reduce expression and/or activity of acetyl-CoA carboxylase transferase, the biotin-dependent enzyme that generates the malonyl-CoA precursor for fatty acid biosynthesis (S9M and S9N Data). Mutants 1 and 2 carry non-synonymous point mutations in *accA* (encoding one component of acetyl-CoA carboxylase transferase), as well as *birA* (encoding the protein that biotinylates acetyl-CoA carboxylase). Mutant 3 carries an early frameshift mutation in *accA*, which only allows for the expression of about 2.5-fold reduced levels (S14E Fig and S9F Data) of an N-terminally truncated and modified, though possibly still partially functional, protein. Considering these observations, it appears plausible that the bactericidal effects caused by FabH inhibition via c1_e_ in wild-type *Chlamydia* could at least in part result from an accumulation of the FabH substrate precursor malonyl-CoA, which the mutations could ameliorate by decreasing malonyl-CoA production. Moreover, in the mutant strains, a decreased flux of malonyl-CoA into fatty acid biosynthesis could increase the amount of branched-chain acyl-CoAs available to re-acylate FabH after catalysis, thereby protecting FabH from the inhibitory action of c1_e_.

In summary, these multiple independent lines of evidence demonstrate that c1_e_, the most potent antichlamydial compound identified in this study, selectively inhibits *Chlamydia* FabH, the enzyme that initiates fatty acid biosynthesis. The compound is a covalent inhibitor of *Chlamydia* FabH and prevents substrate binding by occluding the enzyme’s active site.

## Discussion

In this study, we reported major conceptual additions to the available toolkit for antichlamydial discovery, the identification and in-depth characterization of a set of highly potent and selective antichlamydials chemically distinct from known antibiotics, and the molecular target and MoA of the most potent molecule identified. The significance of this work has two major dimensions. First, by identifying active molecules and biological functions in *Chlamydia* spp. that can be selectively targeted, it promotes the development of more sustainable therapeutics that could aid in ameliorating the threat of antimicrobial resistance. Second, by identifying molecules that can potently but selectively perturb the intracellular growth, host–pathogen interactions, or development of *Chlamydia* spp., it provides tools for advancing our understanding of the pathogens’ unique biological features and virulence strategies.

Summarized in more detail, the key achievements reported in this study were the following: We developed a simple, robust, and information-rich assay for high-throughput screening for non-toxic inhibitors of *Chlamydia* growth (S1 and S2 Fig and S1 Data), applied this assay for a comprehensive search of the chemical space of drug-like small molecules (Figs 1 and S3 and S2 Data), integrated new and previously reported data on antichlamydials into a machine-learning-based model that can predict antichlamydial activities in silico (Fig 2 and S3 Data), and applied this model in a virtual search to probe an even larger chemical space (Fig 2 and S3 Data). Combined, these efforts led to the identification of over 60 potent antichlamydials that are chemically diverse and dissimilar to known antibiotics (Figs 1 and 2 and S4–S6 and S2–S3 Data). In-depth characterization of top molecules revealed that they (with exceptions) were: (a) active in all tested cell lines (Figs 3A and S8 and S5A and S5B Data); (b) non-toxic to host cells at concentrations 30-fold higher than their IC_50_ (Figs 3B and S9 and S5C Data); (c) active against a range of *Chlamydia* strains and species (Fig 3C and S5D Data); (d) selective, i.e., not growth-inhibitory towards microbiota species (Figs 3D and S10 and S11 and S5E–S5H Data); (e) able to prevent inclusion establishment and EB formation when present at the beginning of the infection (Fig 5B and 5C and S7A and S7B Data); (f) able to eradicate established inclusions in a bactericidal manner (Fig 6A and S8A Data); and (g) able to eradicate persistent infections (Fig 6D and 6E and S8B Data). Some compounds also showed synergistic interactions with each other and/or clinically used antibiotics (Figs 4B and S12 and S6B and S6C Data). Moreover, we found that the most potent antichlamydial identified, i.e., compound c1_e_, is a covalent inhibitor of the *Chlamydia* fatty acid biosynthesis protein FabH (Figs 7 and S14–S16 and S9 Data), highlighting the opportunity for selective targeting of this metabolic pathway and the value of the compound as a tool for advancing our understanding of *Chlamydia* biology.

We applied a classic antimicrobial discovery strategy based on the compound-first principle, involving a phenotypic screen for molecules blocking pathogen growth to be later followed by MoA determinations. To date, this strategy has been most commonly applied in antichlamydial discovery (e.g., [41,58–60]), although a few studies described target-based approaches, which involved experimental screening of compound libraries for molecules inhibiting specific targets [61,62] or virtual screening for molecules that bind to selected target structures [63,64]. Target-based approaches require a profound knowledge of the target, such as its structure, molecular function, and biological role [65], hence their applicability to *Chlamydia* remains restricted by our limited understanding of its biology. While powerful in drug development, they are inferior in uncovering new biology. Moreover, while they may uncover molecules that can inactivate a target potently in vitro, these may lack other properties needed for activity in a biological system, such as the ability to penetrate the multiple membrane barriers that shield intracellular bacteria [66].

Phenotypic high-throughput screening of drug libraries requires a simple, fast, and cost-effective setup. Yet, *Chlamydia*’s lifestyle entails a need for detecting intracellular pathogen growth, which makes screening protocols more demanding and explains why the majority of previous screens, with exceptions (e.g., [41,59]), involved rather small compound selections. When compared to previously applied strategies in antichlamydial discovery (e.g., [41,58,59,67–74]), our assay stands out by combining maximum simplicity with cost-effectiveness, and an ability to provide a wealth of information for each compound (Fig 1B). By seeding already pre-infected cells in compound-containing plates, we bypassed many steps carried out individually in most described protocols. Moreover, by omitting medium exchange or wash steps prior to the fluorescence measurements, we significantly lowered the risk for (cross-)contamination or wash-off effects. Moreover, the additional recording of imaging data made the screen more robust, as visual information can help to identify false positives and exclude compounds with non-desired MoA.

It is noteworthy that there are certain possible antichlamydial MoAs that our experimental screening set-up could have missed. This includes for instance antichlamydial activities that do not affect inclusion establishment or bacterial growth, but primarily the formation of EBs capable of establishing a new round of infection. A compound affecting EB maturation in a way compromising its subsequent differentiation into RBs has for instance recently been described [75]. Designing a screening procedure capable of identifying such molecules would be feasible. However, as it would require including a second round of infection, the set-up would be significantly more laborious, cost-intensive, and error-prone.

Because experimental screening will always remain time- and resource-consuming, no matter how simple the assay, an intriguing idea is to exploit available information to first define what properties make a molecule likely to be active and then to use this information for a virtual pre-screening. Along this line, Karhu and colleagues mapped 19 known antichlamydial compounds into the chemical space, based on their physicochemical properties, and then compared these with a library of 502 molecules to select only such with properties most close to the known antichlamydials for experimental testing [76]. More powerful virtual screening for novel antimicrobials can be enabled by training machine-learning-based prediction models on data from compound library screens together with descriptors of chemical structures [77]. We took a similar approach, using data taken from our own experimental screen and the literature to train a binary random forest classifier that can predict the antichlamydial activity of input structures (Fig 2 and S3 Data). Our aim was to leverage available data to identify additional antichlamydials based on chemical similarities.

Along the way of developing the prediction model, we encountered hurdles, such as the problem of class imbalance [78], which required us to employ class balancing techniques to improve model performance, and difficulties in comparing data between studies that employed a plethora of different readouts. These problems may be mitigated in the future by generating an expanded in-house dataset for model training. It is also important to be aware that the prediction model generated in this study is rather simple in nature and cannot be expected to predict antichlamydial activities for molecules that are chemically very different to those included in the training set. As more experimental data and validations become available in the future, the model can be updated, and the database be rescreened, potentially yielding additional antichlamydial molecules. Moreover, a scaffold splitting approach, or similar, preventing the involvement of similar compounds in the training and test sets during model development and validation [79], could in the future help mitigate limitations arising from overfitting and could thereby help generate a model that can better generalize to novel chemical spaces. Nevertheless, despite potential for future improvements, the present model enabled us to screen many more compounds than otherwise feasible and successfully identified novel molecules with potent antichlamydial activities (Fig 2 and S3 Data). As in the present study we chose to restrict experimental validation experiments to compounds that were easily accessible commercially, the full chemical diversity of the antichlamydials uncovered through the virtual screening approach will need to be revealed in follow-up work. Indeed, about 150 additional predicted molecules await experimental validation.

The reasons for a significant number of *Chlamydia* infections to become chronic or recurrent or to prove otherwise recalcitrant to treatment are not fully understood. However, *Chlamydia* persistence could be a major contributing factor [42]. Persistence can be induced by various stress conditions, including nutrient deprivation [80,81] and exposure to cytokines [82], but also exposure to antibiotics, most notably beta-lactams [83,84]. The currently most commonly used therapeutics, doxycycline, and azithromycin, may also induce persistence when used for the bacteria sub-lethal doses [85,86]. Significantly, once in a persistent state, the bacteria are less susceptible to eradication by antibiotic treatment [51,87–89]. Hence, effective therapeutics would be expected to not be prone to induce persistence and to be able to combat persistent infections. In this context, it was reassuring to see that our top compounds did not induce morphological signs of persistence and were able to eradicate, in a bactericidal manner, established (Fig 6A and S8A Data) as well as persistent inclusions (Fig 6D and S8B Data). However, we also identified two new persistence-inducing compounds (S7 Fig and S4 Data), which in the future may aid in deciphering the molecular basis of this poorly understood phenomenon.

Crucially, for novel antichlamydial therapeutics to be more sustainable than those currently available, they would need to be more selective (narrow spectrum), though a broad activity against *Chlamydia* spp. may still be desirable. Indeed, our top compounds were active against a range of *Chlamydia* serovars and species (Fig 3C and S5D Data), suggesting that they could inspire new treatments for various *Chlamydia* diseases, potentially also zoonotic and veterinary infections. Critically, when tested against a representative selection of commensal gut and vaginal microbes, our top compounds displayed striking selectivity, in stark contrast to the broad action of doxycycline and azithromycin (Figs 3D and S10 and S11 and S5E–S5H Data). Yet, ultimately, both efficacy and selectivity will also need to be confirmed in vivo.

Understanding compound MoA can deepen our knowledge of *Chlamydia* biology, identify targets for future target-based discovery approaches, and guide the further chemical optimization of the molecules. Via a combination of approaches, more specifically, thermal proteome profiling (Fig 7A and 7B and S9A and S9B Data) and the analysis of evolved resistant mutants (S14 Fig and S9C–S9F and S9M and S9N Data), we uncovered the fatty acid biosynthesis protein FabH as a potential molecular target of c1_e_. Exploiting the fact that human cells, in contrast to the bacteria, do not synthesize branched-chain fatty acids, we could then demonstrate that c1_e_ inhibits bacterial fatty acid synthesis in infected cell cultures with a dose–response relationship that matches its antichlamydial activity (Fig 7D and 7E and S9G and S9H Data). Crucially, we confirmed that c1_e_ inhibits the catalytic activity of recombinant FabH from *C. trachomatis*, but not *E. coli*, in an in vitro activity assay (Fig 7F and S9I Data), and demonstrated that c1_e_ binds covalently to FabH’s active site thereby impeding the enzyme’s interactions with its natural substrates (Figs 7G and S15B and S9K and S9L Data). The ability of c1_e_ to covalently modify ctFabH at the active site cysteine is likely due to the electrophilic nature of its cyclopropane ring (Fig 7H). When compared to cerulenin, a natural product fatty acid synthesis inhibitor that inhibits beta-ketoacyl synthases in a similar way, but through a reaction between a reactive epoxide and the active site cysteine [90], the significantly less reactive cyclopropane warhead of c1_e_ can be expected to provide an advantage over cerulenin in terms of target selectivity. Overall, our findings underscore the previously recognized, but not yet realized, possibility of exploiting fatty acid biosynthesis in the therapeutic targeting of *Chlamydia* spp. [52,56,91] and identify a molecule that can target this metabolic pathway selectively in *Chlamydia*.

Despite identifying FabH as a major target of c1_e_, we cannot entirely exclude that the molecule could have additional targets contributing to its antichlamydial activity. For instance, thermal proteome profiling suggested two candidate host targets as well (Fig 7A and S9A and S9B Data). Moreover, in relation to its interaction profile, c1_e_ clustered with fluoroquinolones (Fig 4C), suggesting that the compound may also interfere with chlamydial DNA replication. Neither our analyses of the resistant mutants nor thermal proteome profiling suggested respective candidates, such as topoisomerases, yet this possibility should nevertheless be explored in the future. Moreover, it will be of interest to determine the interaction profiles of other bacterial fatty acid biosynthesis inhibitors. Regarding the other compounds, we observed c4_e_ and c9_e_ to cluster with aminoglycosides (Fig 4C), which may suggest that c4_e_ and c9_e_ compromise ribosome (proofreading) function in *Chlamydia*. The latter would also be consistent with the bacteriolytic activity of c4_e_ (Fig 6E), as aminoglycosides are known to lyse bacterial cells [92,93]. On the other hand, our observation that c4_e_ was less effective in autophagy-deficient cells (Fig 5D and S7C Data), while not causing a bulk stress-induced increase in autophagy (Fig 5E and S7D Data), may suggest that this compound sensitizes the pathogen to host cell-autonomous defenses. Future work will test these hypotheses.

Taken together, our innovative multi-strategy antimicrobial discovery approach identified more than 60 potent and in part selective *Chlamydia* growth-inhibitory small molecules, including a covalent inhibitor of *Chlamydia* FabH. In the future, these molecules can serve as tools to transform our understanding of *Chlamydia* pathogenesis, but also as chemical starting points for the development of more sustainable therapeutics, which are urgently needed for this medically challenging and prevalent group of bacterial pathogens.

## Materials and methods

### Cell culture

HeLa (ATCC CCL-2), Vero (ATCC CCL-81), HEK293T (ATCC CRL-3216), BALB/3T3 (ATCC CCL-163), and UMNSAH/DF-1 (ATCC CRL-12203) cells were cultivated in Dulbecco’s Modified Eagle’s Medium (DMEM; Gibco) supplemented with 10% heat-inactivated fetal bovine serum (FBS; Gibco). In experiments involving bulk fluorescence measurements or live cell imaging, a phenol red-free DMEM was used to reduce background. A2EN cells [44] were cultivated in Keratinocyte-SFM (Gibco) and JH4 cells (ATCC CCL-158) in Ham’s F-12 (Kaighn’s) medium (Gibco), both supplemented with 10% heat-inactivated FBS. In experiments including fluorescence measurements in A2EN and JH4 cells, their specific media were replaced by phenol red-free DMEM 2.5 h before the measurements (i.e., prior to resazurin addition). All cell cultures were maintained at 37 °C and 5% CO_2_ in a humidified incubator.

### Generation of STING pathway- and autophagy-deficient cell lines

Cell lines deficient for the indicated genes were generated using CRISPR/Cas9-mediated genome editing [94]. A lentiviral delivery system was used to introduce genes coding for Cas9 nuclease and gene-specific sgRNAs. The following sgRNAs, previously validated by Sanjana and colleagues [95], were cloned into vector lentiCRISPRv2 [95] (Addgene 52961): ATG5 (5′-AGATCAAATAGCAAACCAAT-3′, 5′-TTCCATGAGTTTCCGATTGA-3′), ATG7 (5′-AGAAGAAGCTGAACGAGTAT-3′, 5′-TAGGGTCCATACATTCACTG-3′), NDP52 (5′-CAACAAATCAGCTAAACAGC-3′, 5′-AATCAGAGTGGATCAGCTTC-3′), STING (5′-GGATGTTCAGTGCCTGCGAG-3′, 5′-GGTGCCTGATAACCTGAGTA-3′), TBK1 (5′-CATAAGCTTCCTTCGTCCAG-3′, 5′-ATCACTTCTTTATTCCTACG-3′), IRF3 (5′-GGGAGTGGGATTGTCCAAGC-3′, 5′-GGCACCAACAGCCGCTTCAG-3′). Lentiviral particles were harvested from supernatants of HEK293T cells that had been co-transfected with psPAX2 (Addgene 12260), pMD2.G (Addgene 12259), and the respective sgRNA-encoding derivative of lentiCRISPRv2. After filtration (0.45 µm), virus-containing supernatants were used to transduce A2EN cells in the presence of 8 µg/ml polybrene (Sigma-Aldrich). Cells were co-transduced with lentiviral vectors encoding the two distinct sgRNAs that target the same gene. Transduced cells were selected in the presence of puromycin (1 µg/ml; Gibco) and cloned by limiting dilution.

### Confirmation of gene knockouts by western blot analysis

Protein extracts were prepared by cell lysis in boiling 1% SDS buffer, as previously described [96], separated by SDS PAGE (Mini-PROTEAN TGX 4%–20% gels, Bio-Rad), and transferred onto nitrocellulose membranes (pore size of 0.2 µm, Bio-Rad). Membranes were then blocked for 20 min with 3% BSA in Tris-buffered saline with Tween (TBST; 20 mM Tris (pH 7.5), 150 mM NaCl, 0.1% Tween 20) and incubated overnight at 4 °C with primary antibodies diluted in blocking buffer. The following primary antibodies were used: mouse-anti-β-actin (1:2000; Cell Signaling, 3700), rabbit-anti-ATG5 (1:1000; Cell Signaling, 12994), rabbit-anti-ATG7 (1:1000; Cell Signaling, 8558), rabbit-anti-NDP52 (1:1000; Abcam, ab68588), rabbit-anti-STING (1:1000; Cell Signaling, 13647), rabbit-anti-TBK1 (1:1000; Cell Signaling, 3504), and rabbit-anti-IRF3 (1:1000; Cell Signaling, 11904). After incubation, membranes were washed thrice with TBST, incubated for 1 h with horse radish peroxidase (HRP)-conjugated secondary antibodies (anti-mouse: Biorad, 172-1011; anti-rabbit: Thermo-Fisher-Scientific, G-21234; 1:10,000–1:50,000 diluted in blocking buffer), and washed again thrice with TBST. Membranes were then incubated for 1 min with HRP substrate (SuperSignal West Pico PLUS or SuperSignal West Atto, Thermo-Fisher-Scientific) and chemiluminescent signals were recorded with an Amersham Imager 680RGB (GE Healthcare). Membranes were stripped for 15–30 min with Restore Plus western blot stripping buffer (Thermo-Fisher-Scientific) and blocked with 3% BSA in TBST before the detection of additional targets. Band intensities were quantified using Image Quant TL (GE Healthcare). Expression levels of target proteins were normalized to the expression of β-actin.

### *Chlamydia* strains

Infection experiments were carried out with the following *Chlamydia* strains: *C. trachomatis* L2/434/Bu (CTL2, ATCC VR-902B), an rsGFP-expressing derivative of CTL2 (CTL2-GFP; i.e., CTL2 transformed with plasmid p2TK2-SW2-IncDProm-RSGFP-IncDTerm [97]), *C. trachomatis* A/HAR-13 (CTA; DSMZ, 19440), *C. trachomatis* D/UW-3/Cx (CTD; DSMZ, 19411), *C. trachomatis* E/Bour (CTE; DSMZ, 19131), *C. caviae* GPIC (CC; [98]), and *C. muridarum* MoPn (CM; DSMZ, 28544). Two different procedures were used to prepare infection inocula, i.e., density-gradient purified EBs (of CTL2 and CTL2-GFP) and crude preparations (of all other strains). Both procedures were described in detail in a previous publication [99]. Bacterial preparations obtained by either procedure were resuspended in SPG (sucrose-phosphate-glutamate) buffer (75 g/l sucrose, 0.5 g/l KH_2_PO_4_, 1.2 g/l Na_2_HPO_4_, 0.72 g/l glutamic acid, pH 7.5), briefly sonicated, and stored at −80 °C. Bacteria were titered as previously described [99].

### Screening assay protocol

HeLa cells in suspension were mixed with CTL2-GFP (30 IFU/cell) and then centrifuged (800*g*, 5 min) to enhance infection. Subsequently, the infected cells were seeded (4,000 cells/well) in black transparent bottom 384-well plates (containing compounds or controls) using a Multidrop Combi liquid dispenser (Thermo-Fisher-Scientific) and the plates were incubated for 26 h (37 °C, 5% CO_2_). At that time, 1/5 volume of resazurin (Sigma-Aldrich; 0.15 mg/ml in Dulbecco’s phosphate-buffered saline (DPBS; Gibco)) was added followed by further incubation for 2.5 h. Resorufin and rsGFP fluorescence were measured at a Spark plate reader (Tecan) using wavelengths of 560/590 nm (excitation/emission) and 490/510 nm, respectively. Cells in plates intended for imaging were then fixed with 4% formaldehyde for 20 min at room temperature, washed two times with DPBS, and stored at 4 °C in DPBS until staining. Cells were stained with Hoechst 33342 (Invitrogen, 5 µg/ml in DPBS) for 15 min, washed three times with DPBS, and imaged at an ImageXpress Micro Confocal high-content imaging system (Molecular Devices), using the FITC filter for rsGFP fluorescence and the DAPI filter for Hoechst fluorescence. Image processing and detection of inclusions and host cell nuclei were performed with CellProfiler (version 4.0.7) [100]. Total inclusion area in an image was calculated by multiplying the number of detected inclusions by the mean inclusion area in that image. Wells treated with ciprofloxacin (15 µg/ml) or staurosporine (1 µM) served as positive controls for complete bacterial growth inhibition and host cell toxicity, respectively. Wells treated solely with DMSO (vehicle control) served as negative control for both. Data was normalized to values obtained from DMSO-treated wells, after subtraction of background levels represented by the positive controls.

### Benchmarking the screening assay with antibiotics

Concentration series of chloramphenicol (0.005–500 µg/ml, Sigma-Aldrich), ciprofloxacin (0.003–300 µg/ml, Sigma-Aldrich), doxycycline (0.0002–20 µg/ml, Sigma-Aldrich), erythromycin (0.002–200 µg/ml, Sigma-Aldrich), gentamicin (0.01–1,000 µg/ml, Thermo-Fisher-Scientific), kanamycin (0.01–1,000 µg/ml, Duchefa), spectinomycin (0.02–2000 µg/ml, Sigma-Aldrich), and tetracycline (0.002–200 µg/ml, Molekula) were tested for inhibition of *C. trachomatis* growth and host cell toxicity using the bulk fluorescence readouts of the screening assay protocol (as described above). To mimic the conditions of the screen, the antibiotics were added to the plates before seeding the infected cells (i.e., at 0 hpi). MICs were calculated using GraphPad Prism (version 8.4.3) based on a previously described method of data analysis [101].

### Compound library screen

The compound library screened experimentally was obtained from the Chemical Biology Consortium Sweden (CBCS), which owns a large collection of molecules (>350,000) derived from various sources, in-house and commercial, and such donated by biotech companies. Specifically, we screened the CBCS primary screening set, which includes 36,785 small molecules selected to represent the diversity within the larger collection, but with a bias towards molecules with drug-like profiles. The compounds were obtained pre-dispensed in assay-ready black transparent bottom 384-well plates. Each compound was represented as a single well (final concentration after seeding of cells: 10 µM compound, 0.1% DMSO). Each plate also included the respective control wells, containing DMSO (final concentration 0.1%), ciprofloxacin (final concentration 15 µg/ml), or staurosporine (final concentration 1 µM). The compounds were tested for inhibition of *C. trachomatis* growth and host cell toxicity using the screening assay protocol (as described above). In the hit validation and potency determination steps, compounds were tested in duplicate and triplicate, respectively, with each replicate on separate plates. IC_50_ values were determined by fitting data into dose–response curves using the “[inhibitor] versus response (three parameters)” equation in GraphPad Prism (version 8.4.3). MICs were calculated as described above.

### Validation of selected screening hits by quantitative PCR

HeLa cells grown in 6-well plates were in parallel treated with the compounds (five serial dilutions from 3 to 0.0003 µM) or solvent (DMSO) and infected with CTL2 (5 IFU/cell), followed by centrifugation (1,500*g*, 30 min), and incubation for 28.5 h. Subsequently, total DNA was isolated using the DNeasy Blood & Tissue Kit (Qiagen) and was quantified using the Qubit RNA BR Assay Kit (Thermo-Fisher-Scientific). Bacterial genome copy numbers were determined by quantitative PCR using an adaptation of a previously described assay [102]. PCR runs were conducted on a CFX Connect qPCR machine (Biorad) using the Power SYBR Green PCR Master Mix (Thermo-Fisher-Scientific). Primers used allowed amplification of a part of the CTL2 gene *ompA* (5′-TGCCGCTTTGAGTTCTGCTT-3′, 5′-GTCGATCATAAGGCTTGGTTCAG-3′) and a part of the host gene ACTB encoding beta-actin (5′-GGTGTATCTCTGCCTTACAGATC-3′, 5′-ACAGCCTGGATAGCAACGTACAT-3′). To enable copy number determinations, a standard curve, consisting of six serial dilutions (10^1^–10^6^ copies) of a plasmid containing the respective genes, was included in each PCR run. Bacterial genome copy numbers (based on *ompA*) were normalized to the ACTB copy numbers detected in the same sample. Results are displayed as relative bacterial growth compared to the DMSO-treated control. IC_50_ values were determined as described above.

### Virtual screen and validations

Model development was based on the data from the experimental screen (all screened compounds, except for 380 molecules for which the structures were not available), supplemented with a set of compounds found to have been previously tested for antichlamydial activity according to a literature search. The literature search was conducted in PubMed and Web of Science, including studies published up until May 7th 2020, using the following terms: “antichlamydial”, “novel *Chlamydia* therapy”, “*Chlamydia trachomatis* growth inhibition”, and “*Chlamydia trachomatis* inclusion inhibition”. For obvious reasons, only studies that included information on chemical structures were included. The literature compounds were binarized as hits or non-hits based on the same criterion as applied in the screen (i.e., compounds reported to achieve >50% bacterial growth inhibition at 10 µM were considered hits). It is worth noting that the literature compounds had been tested with different experimental methods and that their antichlamydial activities had been reported in various ways; usually either as IC_50_ or as percent inhibition at a single concentration. In the latter case, an estimated IC_50_ was calculated by extrapolating to 50% inhibition (if the reported inhibition was within 10%–90%; otherwise the compound was excluded). Compounds with a molecular weight of over 1,000 g/mol were also excluded. This left a total of 412 compounds. After curation of the expanded dataset (experimental and literature data), molecular descriptors (1D and 2D) were calculated for all included molecules by PaDEL-Descriptor (version 2.21) [35] with default settings, using as input SMILES strings to represent chemical structures. The molecular descriptors were then used to train a random forest classifier (default settings) with the Weka machine learning tool (version 3.8.4) [103]. Prior to training the model, the synthetic minority oversampling technique (SMOTE), as implemented in Weka, was used to increase the number of instances in the minority (hit) class by a factor of three, and the majority (non-hit) class was undersampled to achieve a final ratio of 1:1 between the classes. The final model could classify an input compound as a hit or a non-hit and reported a prediction score of 0.5–1. The model was then applied to virtually screen the ChEMBL database [39] (release CHEMBL28) in Weka, using the same molecular descriptors (calculated by PaDEL-Descriptor) as in training. A total of 25 selected hit compounds were then purchased (from ChemBridge, TargetMol, or Vitas-M Laboratory) and experimentally tested for antichlamydial activity using the screening assay protocol described above, with bulk GFP fluorescence as the readout.

### Verification of persistence induction by confocal microscopy

Confluent HeLa cells, seeded at 1.5 × 10^4^ cells/well in 8-well chamber slides (Ibidi), were infected with CTL2 (10 IFU/cell) and at the same time treated with compounds (10 µM). Positive control wells for persistence induction were treated with penicillin G (100 U/ml). The cells were incubated for 28 h (37 °C, 5% CO_2_), then fixed with 4% formaldehyde in DPBS for 20 min, and permeabilized for 15 min with 0.2% Triton X-100 in DPBS. Subsequently, the cells were incubated for 20 min in blocking solution (2% BSA in DPBS), and then for 1 h with blocking solution containing primary antibodies (goat-anti-OmpA (1:1000; Thermo-Fisher-Scientific, PA1-7209) and rabbit-anti-Slc1 (1:500; [104])). Cells were then washed thrice with DPBS, incubated for 1 h in blocking solution containing Hoechst 33342 (Invitrogen; 5 µg/ml) and secondary antibodies labeled with AlexaFluor 488 or AlexaFluor 555 (Invitrogen; 1:1000), and then washed again thrice with DPBS. Images were taken at a Leica SP8 confocal laser scanning microscope. Processing of confocal images and measurements of bacterial diameters were performed with LAS X Office (Leica, version 1.4.4).

### Verification of morphological recovery from persistence

HeLa cells in suspension were infected with CTL2-GFP (30 IFU/cell), centrifuged (800*g*, 5 min), and seeded (2 × 10^4^ cells/well) in black transparent bottom 96-well plates (with compound-containing wells (10 µM) and control wells containing DMSO (0.2%) or penicillin G (100 U/ml)). The cells were then incubated for 28 h (37 °C, 5% CO_2_). At that time, the compounds were removed, and the cells were washed with DPBS, followed by the addition of new medium. Cells to be imaged at 0 h post-removal were immediately fixed with 4% formaldehyde as above. Other cells were further incubated for 24 and 48 h, and then fixed. Prior to imaging, the cells were stained with Hoechst 33342 (Invitrogen; 5 µg/ml in DPBS) for 15 min and washed thrice with DPBS. Images were taken with an ImageXpress Micro Confocal high-content imaging system (Molecular Devices) as described above.

### Quantification of EB formation and its recovery from persistence

Quantification of EBs was performed essentially as previously described [98]. Briefly, confluent HeLa cells in 96-well plates were infected with CTL2-GFP (5 IFU/cell) and at the same treated with compounds (at the indicated concentrations). At 40 hpi, cell lysates were prepared by incubation of the cells in sterile water, followed by addition of 1/4 volume of 5× SPG buffer. In experiments measuring recovery from persistence, compounds were washed off at 40 hpi by two washes with compound-free medium, followed by further incubation and preparation of cell lysates at 40 h post-wash-off. To quantify the actual number of infectious particles (i.e., EBs) in the initial inoculum (input) and the lysates collected at 40 hpi or 40 h post-wash-off (output), confluent monolayers of Vero cells in 96-well plates were infected with serial dilutions of the different samples. Inclusion numbers were determined by fluorescence microscopy at 28 hpi, as described above for the imaging of screening plates. Based on the number of inclusions found in the input and the output, the number of EBs formed per infected cell could be determined for each sample. Values obtained for samples containing medium only (blank) were subtracted, and the data was then normalized to values obtained for infected cells treated with DMSO only (solvent control).

### Compound effects in different cell lines

The compounds were first diluted in medium containing CTL2-GFP (media containing 0.3% DMSO or 15 µg/ml ciprofloxacin were included as controls), and then transferred to confluent monolayers of cells in black transparent bottom 96-well plates. The infection dose used was cell-line-dependent: 5 IFU/cell for HeLa, Vero, JH4, and BALB/3T3, 10 IFU/cell for A2EN, and 25 IFU/cell for UMNSAH/DF-1. The plates were centrifuged (1,500*g*, 30 min), and the cells were incubated for 26 h. At that time, 1/5 volume of resazurin (0.15 mg/ml in DPBS) was added followed by further incubation for 2.5 h. Resorufin and bulk GFP fluorescence were measured as described above. Compound effects in STING pathway- and autophagy-deficient A2EN cells were tested in the same way.

### Compound effects against different *Chlamydia* spp.

Compound effects against different *Chlamydia* spp. were tested in a similar way as effects in different cell lines, but instead using different bacterial strains to infect HeLa cells only, with infection doses of 1.5 IFU/cell for CTL2-GFP, 0.5 IFU/cell for CTL2, 2 IFU/cell for CTA, 1 IFU/cell for CTD, CTE, and CC, and 0.3 IFU/cell for CM. After incubation, the cells were fixed and stained with an antibody targeting Slc1, as described above. Images were taken with an ImageXpress Micro Confocal high-content imaging system (Molecular Devices), and inclusion areas were measured with CellProfiler (version 4.0.7). Values from ciprofloxacin-treated wells or wells containing medium only (blank) were subtracted, and the data was normalized to values from DMSO-treated control wells. IC_50_ values were determined as described above.

### Compound effects on commensal microbes in dilution assay

Cultures of *E. coli* (DSM 301), *L. reuteri* (DSM 20016), *L. crispatus* (DSM 20584)*, L. iners* (DSM 13335), *E. faecalis* (DSM 20478), *B. thetaiotaomicron* (DSM 2079), and *B. adolescentis* (DSM 20083) were maintained in a Whitley H35 hypoxystation adapted to anaerobic conditions (37 °C, 0% O_2_, 5% CO_2_). Cultures of *G. vaginalis* (DSM 4944) were maintained in a Whitley H135 HEPA hypoxystation adjusted to microaerophilia (37 °C, 5% O_2_, 5% CO_2_). Culture of *C. albicans* (DSM 1577) was done under normoxic conditions at 30 °C. The following liquid growth media were used in the experiments: MRS medium (*L. crispatus*), New York City III broth supplemented with 10% inactivated horse serum medium (NYCIII, [105]; *L. iners* and *G. vaginalis*), YPD medium (*C. albicans*), and modified Gifu Anaerobic Medium (mGAM, HyServe; all others). Prior to their use in experiments, the microbes were cultivated overnight in liquid growth medium, and then used to inoculate fresh cultures, which were grown to exponential phase. In parallel, 96-well plates containing dilutions of the compounds to be tested were prepared under normoxic conditions in the respective growth media. Azithromycin and doxycycline were included as controls for growth inhibition, and DMSO was included as vehicle control. The plates were then moved to the hypoxystation 3 h before inoculation with the microbes to allow the plates to equilibrate to the anoxic or microaerophilic conditions. Subsequently, the exponentially growing microbes were first diluted to an OD_600_ of 0.5 and then further diluted resulting in the final suspension used to inoculate the compound plates (typically 10^5^–10^6^ microbes/well). The plates were incubated under normoxic conditions at 30 °C for 20 h (*C. albicans*), under microaerophilia (5% O_2_, 5% CO_2_) at 37 °C for 20 h (*G. vaginalis*), or under anaerobic conditions (0% O_2_, 5% CO_2_) at 37 °C for 18–20 h (all other microbes). The incubation was followed by measurements of OD_600_ to estimate microbial growth. Values from wells containing medium only (blank) were subtracted, and the data was normalized to values from DMSO-treated control wells.

### Compound effects on commensal bacteria in radial diffusion assay

Cultures of *B. coccoides* (DSM935), *P. copri* (DSM18205), *B. fragilis* (DSM2151), *D. newyorkensis* (DSM103457), *P. histicola* (DSM26979), *E. faecalis* (DSM 20478), *B. thetaiotaomicron* (DSM2079), and *B. adolescentis* (DSM20083) were incubated under anaerobic conditions (37 °C, 0% O_2_, 5% CO_2_) in a Whitley H35 hypoxystation. *E. coli* (DSM 301) and *L. reuteri* (DSM 20016) were grown aerobically at 37 °C. At the start of the assay, approximately 10^7^ CFU from mid-log cultures of the bacteria were mixed with 10 ml underlay gel consisting of 0.1% EEO-Agarose (Sigma), 0.1% broth powder [brain heart infusion broth (Merck Millipore) supplemented with 5 g/l yeast extract (Gibco) for *B. coccoides*, *B. fragilis*, and *B. thetaiotaomicron*; fastidious anaerobe broth (Neogen) for *P. copri*, *P. histicola*, and *D. newyorkensis*; tryptic soy broth (BD) for *E. coli* and *E. faecalis*; de Man, Rogosa and Sharpe broth (Merck Millipore) for *L. reuteri*; and reinforced clostridial medium (VWR) for *B. adolescentis*], and 10 mM sodium phosphate buffer (pH 7.4) and poured into an empty petri dish. 2 mm holes were punched into the underlay agar and 4 µl of 160 µM compound solution were added to the holes. After 3 h of incubation, 10 ml of nutrient-rich overlay gel, consisting of 3% broth powder, 0.1% EEO-Agarose, and 10 mM sodium phosphate buffer (pH 7.4), were added on top of the underlay gel. The petri dishes were incubated at 37 °C under aerobic (*E. coli* and *L. reuteri*) or anaerobic (all other bacteria) conditions for 24–48 h. The results are reported as diameter of inhibition zone measured in mm.

### Antibiotic interactions

Black transparent bottom 96-well plates were prepared to contain known antibiotics and a selected set of our top compounds, each at its previously determined IC_50_ concentration. The antibiotics/compounds were added in pairwise combinations and single-antibiotic/compound wells were included for comparison. Ciprofloxacin (15 µg/ml) was used as control for complete bacterial growth inhibition and DMSO (0.1%) was used as vehicle control. HeLa cells in suspension were infected with CTL2-GFP (30 IFU/cell), centrifuged (800*g*, 5 min), and seeded in the compound-containing plates at 2 × 10^4^ cells/well. The cells were then incubated for 28.5 h (37 °C, 5% CO_2_), followed by measurements of bulk GFP fluorescence. Data was normalized to values obtained from DMSO-treated wells, after subtraction of background levels represented by values from ciprofloxacin-treated wells. Interaction type (synergistic, antagonistic, or additive) was classified based on calculations of epistasis, as previously described [49].

### Compound effects on STING pathway and autophagy activation

A2EN cells (wild-type) were seeded in 6-well plates at 1.5 × 10^5^ cells/well and incubated overnight (37 °C, 5% CO_2_). Cells were then either left uninfected or were infected with CTL2 (3 IFU/cell), followed by centrifugation (1,500*g*, 30 min) and further incubation. At 20 hpi, cells were treated with compounds (c1_e_ and c2_e_: 0.6 µM; c3_e_ and c5_e_: 3 µM; c4_e_: 4 µM) or a corresponding amount of DMSO. At 8 h post-treatment, protein samples were generated, and western blot analysis was conducted as described above. The following primary antibodies were used: mouse-anti-β-actin (1:2000; Cell Signaling, 3700), rabbit-anti-phospho-STING (1:1000; Cell Signaling, 19781), and rabbit-anti-LC3A/B (1:1000; Sigma-Aldrich, L8918). Band intensities were quantified using Image Quant TL (GE Healthcare). Expression levels of target proteins were normalized to the expression of β-actin. Values from compound-treated samples were then divided by values from DMSO-treated samples.

### Long-term treatment

HeLa cells in suspension were infected with CTL2-GFP (30 IFU/cell), centrifuged (800*g*, 5 min), and seeded in black transparent bottom 96-well plates at 2 × 10^4^ cells/well, followed by incubation (37 °C, 5% CO_2_). At 24 hpi, the medium was replaced with medium containing compounds or antibiotics (azithromycin and doxycycline). The cells were then further incubated, and the compounds were replenished at 48 and 72 hpi, followed by wash-off at 96 hpi. Finally, the cells were incubated until 168 hpi. Measurements of bulk GFP fluorescence were performed every 24 h. To visualize growth over time, data was normalized to values from ciprofloxacin-treated control wells (treated with ciprofloxacin (15 µg/ml) from 0 hpi, representing zero growth) at each time point. Images were also taken at each time point (or every 0.5 h during live cell imaging), using an ImageXpress Micro Confocal high-content imaging system (Molecular Devices). Long-term treatments in the presence of penicillin G (100 U/ml) were performed in a similar fashion.

### Thermal proteome profiling

HeLa cells were seeded in 6-well plates at 1.5 × 10^5^ cells/well and incubated overnight (37 °C, 5% CO_2_). Cells were then infected with CTL2 (5 IFU/cell), followed by centrifugation (1,500*g*, 30 min), and further incubation. At 24 hpi, the medium was replaced with medium containing serial dilutions of c1_e_ (0.1–0.0001 µM) or a corresponding amount of DMSO, followed by further incubation for 60 min. Subsequently, the cells were collected, pooled (from two identically treated wells), washed with DPBS, resuspended in DBPS (220 µl), and transferred to a 96-well PCR plate (20 µl/well, 10 wells). Throughout all the washing steps, the compound was present at the concentration at which the cells had been incubated. The thermal proteome profiling procedure was performed as previously described [106]. Briefly, cells were subjected to a heat treatment with each of the 10 wells per sample being exposed for 3 min to a different temperature between 37 and 67 °C. Subsequently, the cells were lysed with lysis buffer (0.8% NP40, 1 mM MgCl_2_, 1 × cOmplete protease inhibitor, and 0.25 U/µl benzonase in PBS) for 20 min at room temperature followed by three freeze-thaw cycles. The remaining soluble protein fraction at each temperature was collected by filtration using a 0.22 µm filter plate (Millipore; 500*g*, 5 min, 4 °C). Samples were prepared for proteomics by denaturing the samples with a final concentration of 2% SDS and 20 mM TCEP, followed by a modified sp3 protocol [107], as previously described [108]. Briefly, samples were added to a bead suspension (10 µg Sera-Mag Speed Beads (Cytiva) in 10 µl 15% formic acid and 30 µl ethanol) and incubated shaking for 15 min at room temperature. Beads were then washed four times with 70% ethanol. Proteins were digested overnight by adding 40 µl of 5 mM chloroacetamide, 1.25 mM TCEP, and 200 ng trypsin in 100 mM HEPES pH 8.5. Peptides were eluted from the beads and dried under vacuum. Subsequently, they were then labeled with TMTpro (Thermo-Fisher-Scientific), pooled, and desalted with solid-phase extraction using an OASIS HLB µElution Plate (30 µm; Waters). Samples were fractionated onto 48 fractions on a reversed-phase C18 system running under high pH conditions, with every 18th fraction being pooled together. Samples were analyzed by LC-MS/MS using a data-dependent acquisition strategy on a Vanquish Neo LC (Thermo-Fisher-Scientific) coupled with an Orbitrap Exploris 480 (Thermo-Fisher-Scientific). Raw files were processed with MSFragger [109] against a concatenated *Homo sapiens* (UP000005640 from UniProt) and *C. trachomatis* L2/434/Bu (UP001154402 from UniProt) database using standard settings for TMT. Data was normalized using vsn [110]. Data analysis was performed in R using the package TPP2D, as previously described [111]. The top 100 proteins with the highest F-statistic were considered to significantly change in their abundance or thermal stability.

### Generation of resistant mutants

HeLa cells were seeded in 96-well plates at 5 × 10^3^ cells/well and incubated overnight (37 °C, 5% CO_2_). Cells were then infected with CTL2 (1 IFU/cell), followed by centrifugation (1,500*g*, 30 min). In parallel, different wells were treated with three distinct concentrations of c1_e_ corresponding to its IC_50_ (21 nM), 0.5× IC_50_ (10.5 nM), or 2× IC_50_ (42 nM). At 48 hpi, bacteria were harvested from the well that had been treated with the highest concentration still allowing significant bacterial growth (based on inclusion number and size detectable in the inverted cell culture microscope). For this purpose, the cells were lysed by incubation in 100 µl/well sterile water. Subsequently, 1–3 µl of the lysate were transferred to a new plate with HeLa cells (prepared as described above), followed by centrifugation (1,500*g*, 30 min). Again, distinct wells were treated with three different concentrations of c1_e_, corresponding to the concentration that had been used in the well harvested, as well as 0.5× and 2× of that amount. This passage procedure was done 20 times in total with three separately kept evolving mutant lines, whereby the concentration could be increased over time. To confirm resistance development, HeLa cells seeded in black transparent bottom 96-well plates were treated with serial dilutions of c1_e_ and parallelly infected with the evolved strains. At 26 hpi, the cells were fixed, and inclusion areas were quantified as described for the experiments measuring compound effects against different *Chlamydia* spp. Data was normalized to a DMSO-treated control.

### Whole-genome sequencing of resistant mutants

Genomic DNA of wild-type and mutant CTL2 was isolated using a previously described procedure [98]. Sequencing library preparation, Illumina PE150 sequencing, and bioinformatic data analysis were conducted by Novogene (UK) Company Limited. Following quality control, reads were mapped to the reference genome of *C. trachomatis* L2/434/Bu [RefSeq NC_010287.1] using Burrows-Wheeler Aligner (BWA) [112]. SNPs and indels were detected using GATK [113].

### Quantification of *fabH* expression by reverse transcription quantitative PCR (RT-qPCR)

HeLa cells were seeded in 12-well plates at 1 × 10^5^ cells/well and incubated overnight (37 °C, 5% CO_2_). Cells were then infected with CTL2 wildtype or mutant (5 IFU/cell), followed by centrifugation (1,500*g*, 30 min), and further incubation. At 46 hpi, RNA was isolated using the RNeasy Mini Plus kit (Qiagen) and quantified using the Qubit RNA BR Assay Kit (Thermo Fisher Scientific). RT-qPCR was conducted using the Power SYBR Green RNA-to-CT 1-Step Kit (Thermo Fisher Scientific). Per reaction, 10 ng of template RNA were used. Relative expression levels of *fabH* were determined according to the comparative C_T_ method [114], and the resulting delta C_T_ values were further normalized to the mean delta C_T_ value observed for cells infected with CTL2 wildtype. Calculations were conducted separately with either *ompA* mRNA or chlamydial 16S rRNA as reference for normalization, and then the mean was calculated. The following primers were used: *ompA* (5′-CGATTCGTATTGCTCAGCCG-3′, 5′-CTGCGCTAGCTTTCACATCG-3′), 16S (5′-CTGCGGTAATACGGAGGGTG-3′, 5′-TTACCTTTCCGCCTACACGC-3′), and *fabH* (5′- CAGCAAACTCCATTCGTCGC −3′, 5′- GTCCGGCTTCTCAGGATACC-3′).

### Mass spectrometry-based proteomics for quantification of FabH protein levels

HeLa cells were seeded in 6-well plates at 4 × 10^5^ cells/well and incubated overnight (37 °C, 5% CO_2_). Cells were then infected with CTL2 wildtype or mutant 3 (5 IFU/cell), followed by centrifugation (1,500*g*, 30 min), and further incubation. At 29 hpi, cells were treated for 1 h with c1_e_ (0.1 µM) or DMSO (solvent-only). At 30 hpi, protein samples were generated by cell lysis in boiling lysis buffer (50 mM Tris/HCl (pH 7.5), 1% SDS, 0.1 M NaCl), followed by incubation with 0.25 U/µl benzonase (37 °C, 1 h). Mass spectrometry-based proteomics was conducted as described for thermal proteome profiling, yet without TMT labeling and fractionation. Raw data derived from LC-MS/MS runs were processed against a concatenated *Homo sapiens* and *C. trachomatis* L2/434/Bu database as described above using standard settings for label free quantification with match between runs. Data was normalized using vsn [110]. Statistical significance was determined using limma [115].

### Compound effects on branched-chain fatty acid synthesis

HeLa cells were seeded in 24-well plates at 4 × 10^4^ cells/well and incubated overnight (37 °C, 5% CO_2_). Cells were then infected with CTL2 (5 IFU/cell), followed by centrifugation (1,500*g*, 30 min) and further incubation. At 22 hpi, the medium was replaced with medium containing cycloheximide (0.5 µg/ml; Sigma-Aldrich), as well as c1_e_ (1 µM, or as indicated), doxycycline (1 µM), cerulenin (50 µM; Sigma-Aldrich), or equivalent amounts of solvent (DMSO). To respective wells, l-[U-14C]-isoleucine (5 µCi/ml; Revvity) was added immediately after. Following additional incubation for 1 or 2 h, lipid extracts were prepared. In brief, the cells were washed thrice with DPBS and then lysed by a 10-min incubation in 200 µl sterile water. For lipid extraction, the lysates were mixed with 750 µl of a 2:1 (vol/vol) mixture of methanol and chloroform, followed by addition of 250 µl chloroform, 30 s of vortexing, addition of 250 µl water, and another 30 s of vortexing. The lower phase (lipid phase) was then collected, transferred to a scintillation vial, and mixed with four volumes of Optiphase HiSafe 3 liquid scintillation fluid (Revvity), prior to measurement of 14C counts at a Beckman LS 6500 scintillation counter. The average counts in unlabeled infected and uninfected samples were used for background subtraction.

### Production of FabH protein

*E. coli* FabH (ecFabH, NCBI AAC74175.1, UniProt P0A6R0) was produced as described previously [57]. To produce CTL2 FabH (ctFabH, NCBI CAP03931.1, UniProt A0A0H3MKU9), the CTL2 *fabH* gene was synthesized by GenScript and cloned into a previously described modified pRSF-Duet1 vector [57]. The ctFabH protein was then expressed and purified following the same methods used for the production of ecFabH [57]. In this way, we generated two different variants of ctFabH. One variant had the N-terminal sequence MGSSHHHHHHSGSENLYFQ↓SG**RASIW**, where ↓ indicates a TEV protease site and the bold sequence the N-terminal residues of FabH. Despite the presence of the protease site, we only used the non-processed protein [i.e., His6(-fMet)SG-ctFabH] in experiments. The second variant had the N-terminal sequence MGSSHHHHHHSGSENLYFQ↓SGGG**RASIW** containing two extra glycines in the linker to improve TEV protease cleavage efficiency. In experiments, we used the non-processed protein [i.e., His6(-fMet)SGGG-ctFabH] or the processed protein [i.e., SGGG-ctFabH], as indicated. To remove acyl-modifications, the purified protein was treated with 10 mM DTT and then desalted into neutral desalting buffer (10 mM Tris pH 7.0, 200 mM NaCl).

### Synthesis of acyl-CoAs

We synthesized (*S*)-2-methylbutyryl- and isovaleryl-CoA by reacting 15 mg of (*S*)-2-methylbutanoic acid or isovaleric acid with 24 mg of carbonyldiimidazole in 5 ml of acetonitrile (dried with molecular sieves) for 20 min, followed by the addition of ~55 mg of coenzyme A (sodium salt) in 5 ml of 0.1 M sodium phosphate pH 7.5. After an hour the reaction was quenched with acetic acid to 5% and the acyl-CoAs were purified by preparative HPLC over a C18 column from a gradient of 0.5% formic acid to acetonitrile. Pure fractions as judged by LC/MS were pooled and lyophilized.

### FabH enzyme kinetics

FabH kinetics were followed using a previously described continuous spectroscopic UV-vis assay for the production of acetoacetyl-CoA·Mg^2+^ by FabH monitored at 302 nm (calculated *ɛ*_302_ = 17.7 mM^−1^·cm^−1^) [57]. All measurements were performed using a Hewlett Packard 8453 UV-vis spectrophotometer in a semimicro quartz cuvette (Starna Cells) held at 25 °C. The buffer consisted of 100 mM bis–tris propane:HCl (pH 8.0), 50 mM MgCl_2_, 10 mM KCl, 50 mM NaCl. In initial reactions including ctFabH, the concentrations of acetyl-CoA (5–200 µM), malonyl-CoA (5–200 µM), and ctFabH (0.5–20 µM) were varied to find conditions under which reasonable rates could be obtained. The experiments were conducted using His6(-fMet)SG-ctFabH.

### Intact protein mass spectrometry of heterologously expressed ctFabH

The intact ctFabH protein after purification and after various treatments was analyzed by LC/MS. To determine the interaction of purified ctFabH with c1_e_ or acyl-CoAs, we treated acyl-modification free ctFabH at 10 µM with 50 µM c1_e_ or acyl-CoA (synthesized as described above) in neutral desalting buffer for a minimum of 10 min and up to an hour. LC/MS analysis was conducted using a Waters Xevo G2-XS QTof interfaced with a Waters Acquity UPLC. The samples were injected onto a short online desalting column (1.0 × 10 mm, HyperSil Gold CN, Thermo) and after flushing with 0.1 formic acid in water with 2% acetonitrile for 5 min, the proteins were eluted using a gradient to 75% acetonitrile over 10 min. The column temperature was 30 °C and the flow rate was 0.1 ml/min. Proteins were ionized by electrospray operating in positive ion mode with capillary voltage at 3 kV, cone voltage at 35 V, source temp 100 °C, desolvation temp at 350 °C, desolvation gas flow was 600 L/h and cone gas flow was 25 L/h. Mass spectra were acquired in continuum mode with a 1 s scan time across an *m*/*z* range of 200–2000. Protein mass spectra were deconvoluted to give a neutral mass of the intact proteins using Masslynx software and the MaxEnt I algorithm.

### Mass spectrometric identification of c1_e_’s binding site in ctFabH

To prepare samples for LC-MS/MS analysis, ~10 µg of c1_e_-treated ctFabH was suspended in 40 µl of 50 mM triethylammonium bicarbonate (pH 8). A mixture of trypsin/LysC (100 ng) was added, followed by incubation at 37 °C for 4 h with shaking at 1,500 rpm. After digestion, the samples were mixed with 40 µl of acetonitrile and frozen at −20 °C. Before LC/MS/MS analysis, the samples were dried and resuspended in 20 µl of 2% acetonritile/0.1% trifluoroacetic acid. About 100 ng was injected onto a Thermo Acclaim PepMap RSLC C18 trapping column (0.1 mm × 20 mm) and washed for ~5 min with 0.1% formic acid using a Thermo EASYnLC system at 0.4 µl/min maintained at 50 °C. Retained peptides were eluted over 35 min onto a Thermo Acclaim PepMAp RSLC resolving column (0.075 mm × 250 mm) with a gradient from 8% to 35% acetonitrile buffer (80% acetonitrile, 20% water + 0.1% formic acid). Eluted peptides were injected into a ThermoScientific Q-Extractive HF-X mass spectrometer using a FlexSpray ion source. Survey scans were taken in the Orbi trap (60,000 resolution, determined at *m*/*z* 200) and the top 10 ions in each survey scan were subjected to automatic higher energy collision induced dissociation with fragment spectra acquired at a resolution of 15,000. The resulting MS/MS spectra were converted to peak lists using Mascot Distiller, v2.8.5 (www.matrixscience.com) and searched against a protein sequence database containing the ctFabH sequence appended with all *E. coli* protein sequences and common laboratory contaminants using the Mascot1 searching algorithm, v3.1.0 [116]. The Mascot output was then analyzed using Scaffold, v5.3.3 (www.proteomesoftware.com) to probabilistically validate protein identifications. Assignments validated using the Scaffold 1% FDR confidence filter were considered true.

### Statistics

Statistical analyses were performed with Graphpad Prism (version 8.4.3). Statistical significance is indicated as follows: *, *P* ≤ 0.05; **, *P* ≤ 0.01; ***, *P* ≤ 0.001; ****, *P* ≤ 0.0001. Principal component analysis and hierarchical clustering were done with SIMCA (version 17; Sartorius). Tanimoto coefficients (CDK standard) were calculated using a previously developed KNIME workflow [117].

## Supporting information

S1 Fig Development of a screening assay for novel chemical inhibitors of *Chlamydia trachomatis* growth. **(A)** Outline of the main steps and measurements in the screening assay. **(B)** Resorufin fluorescence at different HeLa cell seeding densities (mean ± SD, *n* = 3). The table shows Pearson’s r from a seeding density of 0 cells/well to the seeding density indicated. **(C)** GFP fluorescence at different infection doses (IFU/cell), with a seeding density of 4,000 cells/well (mean ± SD, *n* = 3, two-way ANOVA with Sidak’s multiple comparisons test of untreated vs. ciprofloxacin). **(D)** Host cell viability (via resorufin fluorescence) at different infection doses, with a seeding density of 4,000 cells/well (mean ± SD, *n* = 3, one-way ANOVA with Dunnett’s multiple comparisons test of infected vs. uninfected cells). **(E)** Representative images of HeLa cells infected in suspension with different amounts of CTL2-GFP. Since infection in suspension is less efficient than infection of adherent cells, even a dose of 30 IFU/cell left a significant number of cells uninfected. Scale bar is 10 µm. **(F)** DMSO tolerance of CTL2-GFP and HeLa cells, as measured by GFP and resorufin fluorescence, respectively (mean ± SD, *n* = 2 and *n* = 3, respectively, one-way ANOVA with Dunnett’s multiple comparisons test vs. 0% DMSO). **(G, H)** Plate uniformity and signal variability of the bacterial growth inhibition assay, based on measuring GFP fluorescence derived from CTL2-GFP (G), and the corresponding resazurin-based assay for host cell viability (H). Displayed are data from a representative of three independent experiments. In the left panels, each dot denotes a single well. NC, M, and PC refer to negative control, midlevel, and positive control for bacterial growth inhibition (G) and host cell toxicity (H), respectively. The data underlying this figure can be found in S1 Data.
(TIF)

S2 Fig Assay performance benchmarked with clinical antibiotics. **(A)** Eight clinical antibiotics were tested for bacterial growth inhibition and host cell viability with the bulk fluorescence readouts of the screening assay protocol (mean ± SD, *n* = 3). **(B)** MICs of eight antibiotics as determined in (A) (mean of *n* = 3) and compared with previously reported data. The calculated MIC of erythromycin is likely less accurate, due to the shape of its dose-response curve (see (A)). Beta-lactam antibiotics were not included in this analysis, as the plasmid driving GFP-expression in CTL2-GFP also encodes a beta-lactamase. The data underlying this figure can be found in S1 Data.
(TIF)

S3 Fig Decision tree for classification of screening compounds as hits or non-hits. The decision tree enabled integration of information from both bulk fluorescence measurements and high-content imaging for hit selection. Inclusion area was selected as the parameter of choice for image-based assessment of bacterial growth, as all compounds that would have been hits according to inclusion count were also hits according to inclusion area, but not vice versa.
(TIF)

S4 Fig Chemical structures of the 52 priority compounds selected from the experimental compound library screen. The structures were drawn based on their SMILES strings, using OpenBabel (version 3.0.0).
(TIF)

S5 Fig Dose-response curves of the 52 priority compounds selected from the experimental compound library screen. Data is shown for bulk GFP fluorescence as well as total inclusion area (mean ± SD, *n* = 3). The lines indicate the curve fits used for IC_50_ calculation, and the IC_50_ values are given in the respective plots. The data underlying this figure can be found in S2 Data.
(TIF)

S6 Fig Chemical structures of the 25 predicted antichlamydials selected for experimental validation. The structures were drawn based on their SMILES strings, using OpenBabel (version 3.0.0). Compounds c1_v_–c12_v_ displayed antichlamydial activity with IC_50_ values below 10 µM and compounds c13_v_–c19_v_ with IC_50_ values between 10 and 30 µM.
(TIF)

S7 Fig Image analysis-based discovery of persistence-inducing compounds. **(A)** Persistence-inducing compounds identified by inspection of images from the experimental compound library screen. Scale bar is 10 µm. **(B)** Structures of the identified compounds, drawn based on their SMILES strings, using OpenBabel (version 3.0.0). **(C, D)** Confocal microscopic confirmation of a persistent phenotype (formation of ABs) in HeLa cells infected with CTL2 and exposed to the indicated compounds. (C) Representative images of one of three experiments. Bacteria were detected using antibodies specific for OmpA (the major outer membrane protein) and Slc1 (a type III secretion chaperone). The annotated lines show the diameter of representative individual bacteria. Scale bar is 5 µm. (D) Size of compound-treated bacteria (collated data of *n* = 3, line at mean, 80 bacteria measured per condition and experiment, two-way ANOVA with Dunnett’s multiple comparisons test). **(E)** Recovery of the growth of CTL2-GFP in HeLa cells after compound removal. The images are representative of three experiments. Scale bar is 10 µm. **(F)** EB formation by CTL2-GFP and its recovery from persistence in HeLa cells after exposure to the compounds (10 µM) or penicillin G (100 U/ml) for 40 h (mean ± SD, *n* = 3). Data was normalized to values from DMSO-treated wells at wash-off (40 hpi), unpaired t tests with Holm-Sidak correction for multiple comparisons. PEN, penicillin G. The data underlying this figure can be found in S4 Data.
(TIF)

S8 Fig Dose-response curves of selected top compounds in different host cell lines. **(A)** Growth inhibition by top compounds from the experimental compound library screen. The data is based on measurements of bulk GFP fluorescence and shows separate curves from three biological replicates. Error bars indicate standard deviations of three technical replicates. The lines indicate the curve fits used for IC_50_ calculation. **(B)** Growth inhibition by top compounds from the virtual screen, presented as in (A). The data underlying this figure can be found in S5 Data.
(TIF)

S9 Fig Toxicity of selected top compounds against different host cell lines. **(A)** Host cell viability after exposure to top compounds from the experimental compound library screen. The data is based on measurements of bulk resorufin fluorescence (mean ± SD, *n* = 3, two-way ANOVA with Dunnett’s multiple comparisons test of the lowest tested concentration of each compound vs. other concentrations). **(B)** Host cell viability after exposure to top compounds from the virtual screen, presented as in (A). The data underlying this figure can be found in S5 Data.
(TIF)

S10 Fig Effect of top compounds on the growth of gut bacteria. **(A)** Effect of top compounds on the growth of five species of gut bacteria in dilution assays. The bacteria were grown in liquid medium containing compounds for 18 h, at which point OD_600_ was measured. Data was normalized to values obtained from DMSO-treated wells (mean ± SD, *n* = 3, two-way ANOVA with Sidak’s multiple comparisons test of each compound and concentration vs. the mean of all 0.03 µM samples for a particular species). **(B)** Effect of c1_e_–c3_e_ on the growth of additional gut bacteria species in radial diffusion assays (mean ± SD, *n* = 2–5, one-way ANOVA with Dunnett’s multiple comparisons test vs. non-inhibited DMSO-treated controls). Please note that the radial diffusion assay has limitations. It primarily produces qualitative results by indicating the presence or absence of antimicrobial activity and the diameters of inhibition zones can be influenced by various factors, including solubility and diffusion rate of the compounds. However, the more quantitative dilution assay was not applicable to all tested species, as for some species we encountered inconsistent bacterial growth in this format. AZM, azithromycin; DOX, doxycycline. The data underlying this figure can be found in S5 Data.
(TIF)

S11 Fig Effect of top compounds on the growth of four species of the vaginal microbiota. The microbes were grown in liquid medium containing compounds for 18–20 h, at which point OD_600_ was measured. Data was normalized to values obtained from DMSO-treated wells (mean ± SD, *n* = 3, two-way ANOVA with Sidak’s multiple comparisons test of each compound and concentration vs. the mean of all 3 µM samples for a particular species). AZM, azithromycin; DOX, doxycycline. The data underlying this figure can be found in S5 Data.
(TIF)

S12 Fig Determination of between-compound interactions and interactions with clinical antibiotics. Bacterial growth inhibition in HeLa cells infected with CTL2-GFP (30 IFU/cell, 28.5 hpi) and classification of interaction type for all pairwise combinations of 15 selected top compounds with 12 clinical antibiotics (and c1_e_–c3_e_), tested at IC_50_ (mean ± SD, *n* = 3). Interaction type was classified based on calculations of epistasis, as described in the methods. Darker colors indicate stronger synergistic (green) or antagonistic (blue) interactions. Data for c1_e_–c5_e_ were also included in Fig 4B. The data underlying this figure can be found in S6 Data.
(TIF)

S13 Fig Confirmation of gene knockouts by western blot analysis. Western blot analysis of A2EN cell lines (wild-type (WT) or knockout (KO) for indicated genes) to confirm the absence of the targeted proteins in autophagy or the STING-pathway of the type I IFN response. Marked in green are the KO cell clones used in this study. The raw images of the blots are shown in S1 Raw Images.
(TIF)

S14 Fig CTL2 strains resistant to c1_e_ express increased levels of FabH. **(A)** Increase in the concentrations of c1_e_ used during parallel passaging of three CTL2 strains evolving resistance (two-way ANOVA with Dunnett’s multiple comparisons test vs. passage 1). The starting concentration was 10.5 nM (0.5× IC_50_). Please note that the generation of resistant mutants was conducted solely for the purpose of target identification, not to estimate rates of resistance development. **(B)** Growth of the three evolved CTL2 strains in HeLa cells treated with the indicated concentrations of c1_e_, as measured by inclusion area. Bacterial growth is displayed relative to the DMSO control of the respective strain (mean ± SD, *n* = 3, two-way ANOVA with Dunnett’s multiple comparisons test vs. Wildtype). **(C)** Scheme illustrating the location of the mutation upstream of *fabH* (modified nucleotide marked in blue). **(D)** Expression levels of *fabH* in the three evolved CTL2 strains during infection of HeLa cells, as measured at 46 hpi by quantitative reverse transcription PCR (qRT-PCR) (mean ± SD, *n* = 3, one-way ANOVA with Dunnett’s multiple comparisons test vs. Wildtype). **(E)** Volcano plot displaying bacterial proteins up- or downregulated during infection of HeLa cells with the c1_e_-resistant CTL2 mutant 3 compared to infection with wild-type CTL2. Displayed are only data from the analysis conducted in the absence of c1_e_. The full dataset is available in S9F Data. The data underlying this figure can be found in S9 Data.
(TIF)

S15 Fig Binding of c1_e_ to ctFabH prevents substrate interaction. **(A)** Intact protein mass spectrometry of recombinant ctFabH after purification from *Escherichia coli*, demonstrating a large proportion of the protein to be modified by acyl groups, primarily pentanoyl-groups. **(B)** Intact protein mass spectrometry of purified acyl-modification-free ctFabH (10 µM) after sequential 10-min incubations with isovaleryl-CoA (IV-CoA, 50 µM) and c1_e_ (50 µM) in the indicated (>) order. Note that prior incubation with isovaleryl-CoA or c1_e_ prevents interaction with the respective other molecule during the second incubation step. The expected mass difference for +isovaleryl is +84.0 Da and for +c1_e_ is +240.0 Da. The data underlying this figure can be found in S9 Data.
(TIF)

S16 Fig Mass spectrometric analysis demonstrating c1_e_ binding to the active site cysteine of ctFabH. **(A)** Two representative MS/MS fragmentation spectra for a sample containing 10 µM ctFabH and 50 µM c1_e_. The predicted y- and b-ions are shown along the top of the figures and the fragments found in the spectra are marked in bold. The c1_e_-cysteine adduct is represented by the C+240 label. **(B, C)** AlphaFold3 model of a ctFabH dimer with the active site cysteine shown as spheres (black for carbon and gold for sulfur) and the active site peptide observed by mass spectrometry shown in magenta (B). A close-up of the active site peptide (C).
(TIF)

S1 Data Development of a screening assay for novel chemical inhibitors of *Chlamydia trachomatis* growth. The data underlying S1 and **S2 Figs**. (**A)** Resorufin fluorescence at different HeLa cell seeding densities. Data are displayed in S1B Fig. **(B)** GFP fluorescence at different infection doses. Data are displayed in S1C Fig. **(C)** Host cell viability (via resorufin fluorescence) at different infection doses. Data are displayed in S1D Fig. **(D)** DMSO tolerance of CTL2-GFP and HeLa cells, as measured by GFP and resorufin fluorescence, respectively. Data are displayed in S1F Fig. **(E)** Plate uniformity and signal variability of the bacterial growth inhibition assay, based on measuring GFP fluorescence derived from CTL2-GFP. Replicate 1 is displayed in S1G Fig. **(F)** Plate uniformity and signal variability of the host cell viability assay, based on measuring resorufin fluorescence. Replicate 1 is displayed in S1H Fig. **(G)** Eight clinical antibiotics tested for bacterial growth inhibition and host cell viability with the bulk fluorescence readouts of the screening assay protocol. Data displayed in S2A Fig. **(H)** MICs of eight antibiotics as determined in this study and compared with previously reported data. Data displayed in S2B Fig.
(XLSX)

S2 Data Identification of novel antichlamydials through experimental compound library screening. The data underlying Figs 1 and S5. **(A)** Chemical diversity of the screening library of 36,785 compounds, illustrated by principal component analysis based on molecular descriptors calculated with PaDEL-Descriptor. Data are displayed in Fig 1A. **(B)** Host cell viability and *Chlamydia* growth data from the experimental compound library screen. Data are displayed in Fig 1C and 1D. **(C)** Host cell viability and *Chlamydia* growth data from the retesting of the 271 hits from the experimental compound library screen. Data are displayed in Fig 1F–1I. **(D)** Estimated IC50 of the 271 hits. Data are displayed in Fig 1H. **(E)** Maximal structural similarity, represented by Tanimoto coefficients, between the hits and 506 known antibiotics in the Drug Repurposing Hub. Data are displayed in Fig 1I. **(F)** The 52 priority compounds from the experimental compound library screen, tested at 11 concentrations in triplicate. Data are displayed in Figs 1J and 1K, and S5. **(G)** Quantitative PCR-based validation of antichlamydial activity of selected compounds identified in the experimental compound library screen. Data are displayed in Fig 1K.
(XLSX)

S3 Data Predictive modeling enabling antichlamydial discovery through virtual screening. The data underlying Fig 2. **(A)** Compilation of compounds previously tested for antichlamydial activity. **(B)** An area under the receiver operating characteristic curve (ROC-AUC) plot showing the performance of the model after training. Data are displayed in Fig 2B. **(C)** An area under the precision-recall curve (PRC-AUC) plot showing the performance of the model after training. Data are displayed in Fig 2C. **(D)** Histogram of predicted antichlamydial hits from a virtual screen of the ChEMBL database. Data are displayed in Fig 2D. **(E)** Maximal structural similarity to known antibiotics (Tanimoto coefficients) and quantitative estimate of drug-likeness (QED) of the filtered hits. Data are displayed in Fig 2E. **(F)** The 174 hits from the virtual screen remaining after a series of filtering steps. **(G)** Potency of 25 compounds subjected to experimental testing, as measured by bulk GFP fluorescence using the screening assay protocol. Data are displayed in Fig 2F. **(H)** The 25 experimentally tested compounds from the virtual screen, tested at 11 concentrations in triplicate. A part of the data is displayed in Fig 2G. **(I)** Quantitative PCR-based validation of antichlamydial activity of selected compounds identified through virtual screening. Data are displayed in Fig 2G.
(XLSX)

S4 Data Image analysis-based discovery of persistence-inducing compounds. The data underlying S7 Fig. **(A)** Size of bacteria after treatment with persistence-inducing compounds. Data are displayed in S7D Fig. **(B)** EB formation by CTL2-GFP and its recovery from persistence in HeLa cells after exposure to the compounds or penicillin G for 40 h. Data are displayed in S7F Fig.
(XLSX)

S5 Data Determination of antibacterial selectivity of our best antichlamydials. The data underlying Figs 3 and S8–S11. **(A)** Potency of selected top compounds in different cell lines infected with CTL2-GFP, as measured by bulk GFP fluorescence. Data are displayed in Fig 3A. **(B)** Growth inhibition by selected top compounds in different host cell lines. The data is based on measurements of bulk GFP fluorescence. Data are displayed in S8A and S8B Fig. **(C)** Toxicity of selected top compounds against different host cell lines. The data is based on measurements of bulk resorufin fluorescence. Data are displayed in Figs 3B and S9A and S9B. **(D)** Potency of selected top compounds in HeLa cells infected with the indicated *Chlamydia* strains, as measured by inclusion area. Data are displayed in Fig 3C. **(E)** Effect of top compounds on the growth of five species of gut bacteria in dilution assays. Data are displayed in Figs 3D and S10A. **(F)** Effect of top compounds on the growth of microbiota species in comparison to their IC50 and MIC against *Chlamydia trachomatis* (as reported in S2F Data and S3H Data). **(G)** Effect of c1_e_–c3_e_ on the growth of additional gut bacteria species in radial diffusion assays. Data are displayed in S10B Fig. **(H)** Effect of top compounds on the growth of four species of the vaginal microbiota in dilution assays. Data are displayed in S11 Fig.
(XLSX)

S6 Data Determination of between-compound interactions and interactions with clinical antibiotics. The data underlying Figs 4 and S12. **(A)** Determination of between-antibiotic interactions in pairwise combinations of 12 clinical antibiotics. Data are displayed in Fig 4A. **(B)** Determination of interactions in pairwise combinations of selected compounds with antibiotics. Data are displayed in Figs 4B and S12. **(C)** Determination of between-compound interactions in pairwise combinations of selected compounds. Data are displayed in Figs 4B and S12.
(XLSX)

S7 Data Determining the stage in *Chlamydia* development targeted by our best antichlamydials. The data underlying Fig 5. **(A)** Effect of top compounds on EB formation in HeLa cells infected with CTL2-GFP. Data are displayed in Fig 5B. **(B)** Growth inhibition after addition of top compounds at different points in the developmental cycle, tested in HeLa cells infected with CTL2-GFP, as measured at 48 hpi by bulk GFP fluorescence. Data are displayed in Fig 5C. **(C)** Antichlamydial activity of our best compounds in A2EN cells (wild-type or mutant as indicated) infected with CTL2-GFP, as measured at 28.5 hpi by bulk GFP fluorescence. Data are displayed in Fig 5D. **(D)** LC3 lipidation and STING phosphorylation after compound exposure in uninfected A2EN cells and cells infected with CTL2. Data are displayed in Fig 5E and 5F.
(XLSX)

S8 Data Compounds can eradicate both established and persistent infections in a bactericidal manner. The data underlying Fig 6. **(A)** Time course of bacterial growth in HeLa cells infected with CTL2-GFP and treated with different concentrations of c1_e_–c5_e_ or azithromycin or doxycycline. Data are displayed in Fig 6A and 6B. **(B)** Time course of bacterial growth in HeLa cells infected with CTL2-GFP in the presence of penicillin G (present throughout the experiment) and treated with c1_e_–c5_e_ from 24 hpi with daily replenishment. Data are displayed in Fig 6D.
(XLSX)

S9 Data Identification of *Chlamydia* FabH as molecular target of c1_e_. The data underlying Figs 7 and S14–S15. **(A)** Results from two-dimensional thermal proteome profiling. logfc columns display the log2 fold-changes in intensity compared to the untreated control at each temperature. Intensity columns display the log2 signal intensity from the mass spectrometer. Data are displayed in Fig 7A and 7B. **(B)** Statistical analysis of two-dimensional thermal proteome profiling data using the TPP2D package. Abundance/stability score was calculated as the square root of the difference of residual sum squares of the null model (RSS0) and the alternative model (RSS1) and reflects how much more variance is explained by a dose–response model compared to a linear model. log2_Fstat is the log2(F-statistic+1) calculated from the TPP2D package. Data are displayed in Fig 7A. **(C)** Increase in the concentrations of c1_e_ used during parallel passaging of three CTL2 strains evolving resistance. Data are displayed in S14A Fig. **(D)** Growth of the three evolved CTL2 strains in HeLa cells treated with the indicated concentrations of c1_e_, as measured by inclusion area. Data are displayed in S14B Fig. **(E)** Expression levels of *fabH* in the three evolved CTL2 strains during infection of HeLa cells, as measured at 46 hpi by qRT-PCR. Data are displayed in S14D Fig. **(F)** Results from the mass spectrometric comparison of the proteomes of wild-type CTL2 and the c1_e_-resistant mutant 3. The analysis included four samples: WT_DMSO (infected with wild-type CTL2, treated with solvent-only during last hour), WT_c1e (infected with wild-type CTL2, treated with 0.1 µM c1_e_ during last hour), R3_DMSO (infected with resistant CTL2 mutant 3, treated with solvent-only during last hour), R3_c1e (infected with resistant CTL2 mutant 3, treated with 0.1 µM c1_e_ during last hour), each in triplicate. Statistical significance was determined using limma as described in the methods. Data are displayed in S14E Fig. **(G)** Inhibition of chlamydial branched chain fatty acid biosynthesis by c1_e_ and CER but not DOX. Data are displayed in Fig 7D. **(H)** Inhibition of chlamydial branched chain fatty acid biosynthesis by different concentrations of c1_e_. Data are displayed in Fig 7E. **(I)** Enzyme kinetics progress curves of CTL2 FabH (ctFabH) and *Escherichia coli* FabH (ecFabH) with acetyl-CoA and malonyl-CoA substrates. Data are displayed in Fig 7F. **(J)** Intact protein mass spectrometry of recombinant ctFabH after purification from *E. coli*, demonstrating a large proportion of the protein to be modified by acyl groups, primarily pentanoyl groups. Data are displayed in S15A Fig. **(K)** Intact protein mass spectrometry of purified acyl-modification-free ctFabH after incubation with the indicated acyl-CoAs or c1_e_, revealing covalent modification of ctFabH by c1_e_. Data are displayed in Fig 7G. **(L)** Intact protein mass spectrometry of purified acyl-modification-free ctFabH after sequential 10-min incubations with isovaleryl-CoA (IV-CoA) and c1_e_ in the indicated (>) order. Data are displayed in S15B Fig. **(M)** Single nucleotide polymorphisms in the genomes of the three resistant CTL2 mutants. **(N)** Insertions and deletions in the genomes of the three resistant CTL2 mutants.
(XLSX)

S1 File Raw Images. Raw images of western blots shown in S13 Fig.
(PDF)

## Abbreviations

**:**
ABs: aberrant bodies
AZM: azithromycin
CBCS: Chemical Biology Consortium Sweden
CC: *C. caviae*
CER: cerulenin
CIP: ciprofloxacin
CM: *C. muridarum;*
CTA: *C. trachomatis* A
CTD: *C. trachomatis* D
CTE: *C. trachomatis* E
CTL2: *C. trachomatis* L2
DMSO: dimethyl sulfoxide
DOX: doxycycline
DPBS: Dulbecco’s phosphate-buffered saline
EB: elementary body
GFP: Green Fluorescent Protein
Hpi: hours post-infection
HRP: horse radish peroxidase
IC_50_: half-maximal inhibitory concentration
IFUs: inclusion-forming units
MIC: minimum inhibitory concentration
MoA: mode of action
PEN: penicillin G
PRC-AUC: area under the precision-recall curve
QED: quantitative estimate of drug-likeness
RB: reticulate body
ROC-AUC: area under the receiver operating characteristic curve
STIs: sexually transmitted infections
