# Supplementary figures and images for: A multi-strategy antimicrobial discovery approach reveals new ways to treat Chlamydia
Source: PLoS Biol. 2025 Apr 29;23(4):e3003123. doi: 10.1371/journal.pbio.3003123 (PMC12040169; doi:10.1371/journal.pbio.3003123)

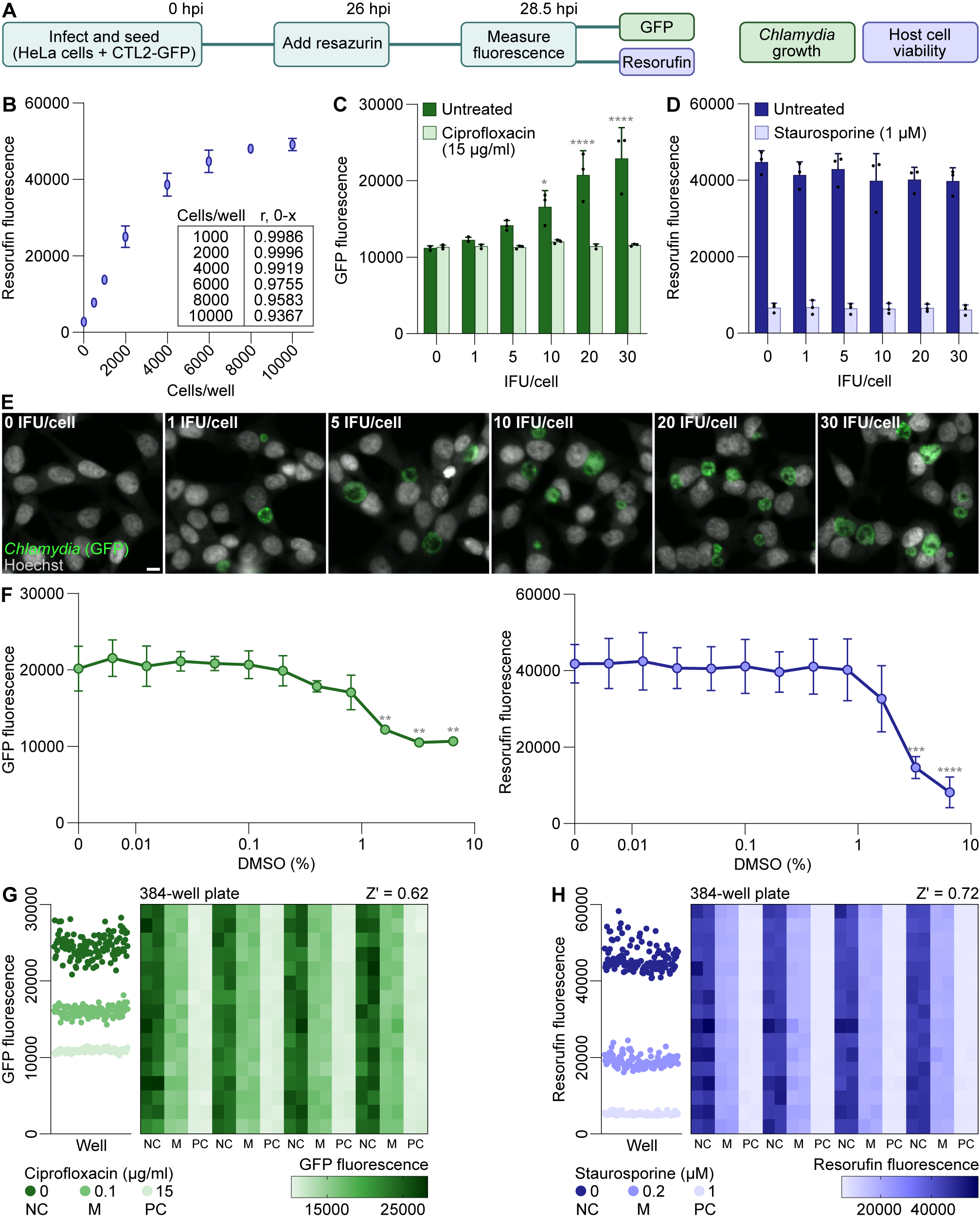

Supplement: S1 Fig — (A) Outline of the main steps and measurements in the screening assay. (B) Resorufin fluorescence at different HeLa cell seeding densities (mean ± SD, n = 3). The table shows Pearson’s r from a seeding density of 0 cells/well to the seeding density indicated. (C) GFP fluorescence at different infection doses (IFU/cell), with a seeding density of 4,000 cells/well (mean ± SD, n = 3, two-way ANOVA with Sidak’s multiple comparisons test of untreated vs. ciprofloxacin). (D) Host cell viability (via resorufin fluorescence) at different infection doses, with a seeding density of 4,000 cells/well (mean ± SD, n = 3, one-way ANOVA with Dunnett’s multiple comparisons test of infected vs. uninfected cells). (E) Representative images of HeLa cells infected in suspension with different amounts of CTL2-GFP. Since infection in suspension is less efficient than infection of adherent cells, even a dose of 30 IFU/cell left a significant number of cells uninfected. Scale bar is 10 µm. (F) DMSO tolerance of CTL2-GFP and HeLa cells, as measured by GFP and resorufin fluorescence, respectively (mean ± SD, n = 2 and n = 3, respectively, one-way ANOVA with Dunnett’s multiple comparisons test vs. 0% DMSO). (G, H) Plate uniformity and signal variability of the bacterial growth inhibition assay, based on measuring GFP fluorescence derived from CTL2-GFP (G), and the corresponding resazurin-based assay for host cell viability (H). Displayed are data from a representative of three independent experiments. In the left panels, each dot denotes a single well. NC, M, and PC refer to negative control, midlevel, and positive control for bacterial growth inhibition (G) and host cell toxicity (H), respectively. The data underlying this figure can be found in S1 Data. (TIF) [file pbio.3003123.s001.tif]

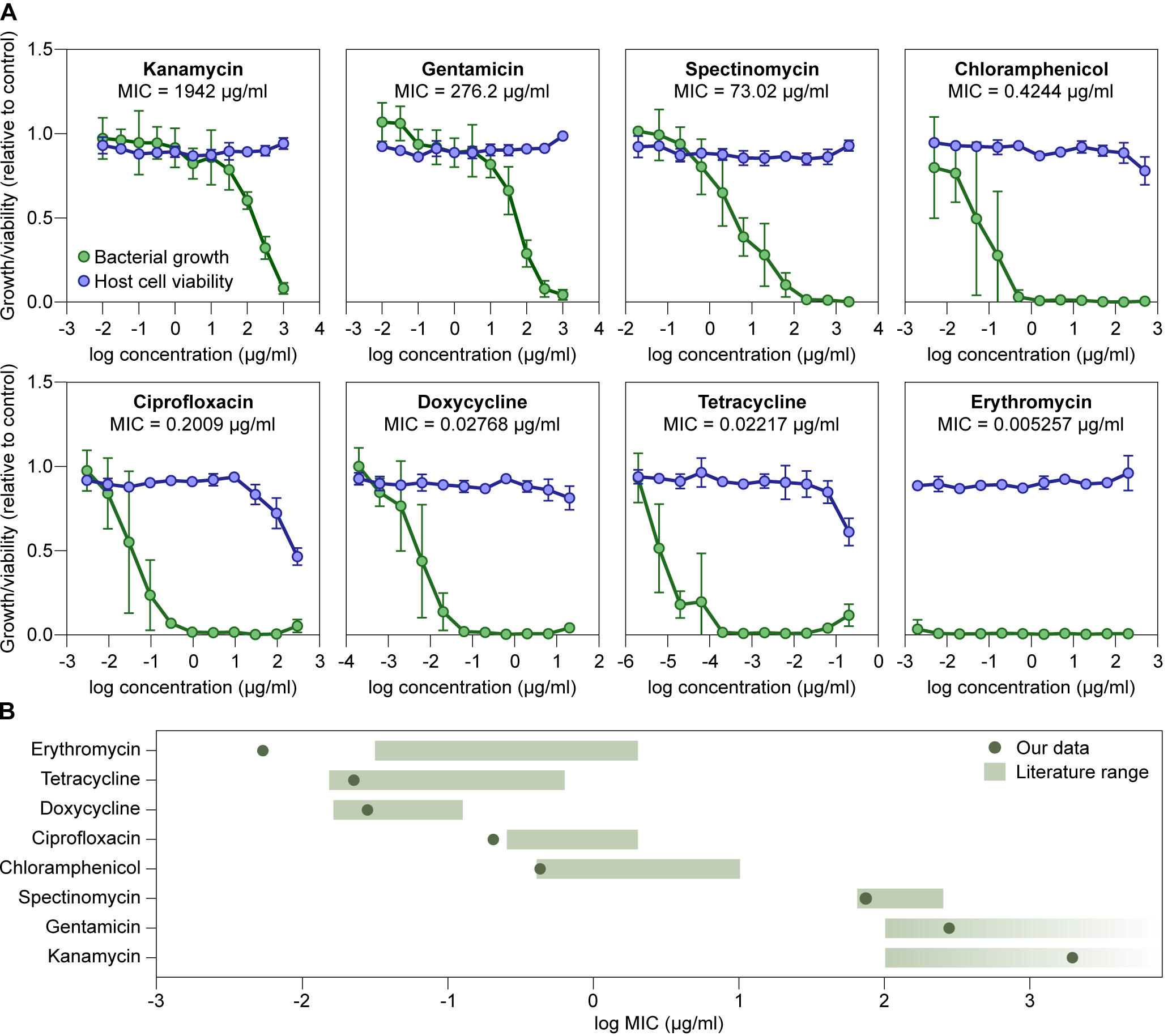

Supplement: S2 Fig — (A) Eight clinical antibiotics were tested for bacterial growth inhibition and host cell viability with the bulk fluorescence readouts of the screening assay protocol (mean ± SD, n = 3). (B) MICs of eight antibiotics as determined in (A) (mean of n = 3) and compared with previously reported data. The calculated MIC of erythromycin is likely less accurate, due to the shape of its dose-response curve (see (A)). Beta-lactam antibiotics were not included in this analysis, as the plasmid driving GFP-expression in CTL2-GFP also encodes a beta-lactamase. The data underlying this figure can be found in S1 Data. (TIF) [file pbio.3003123.s002.tif]

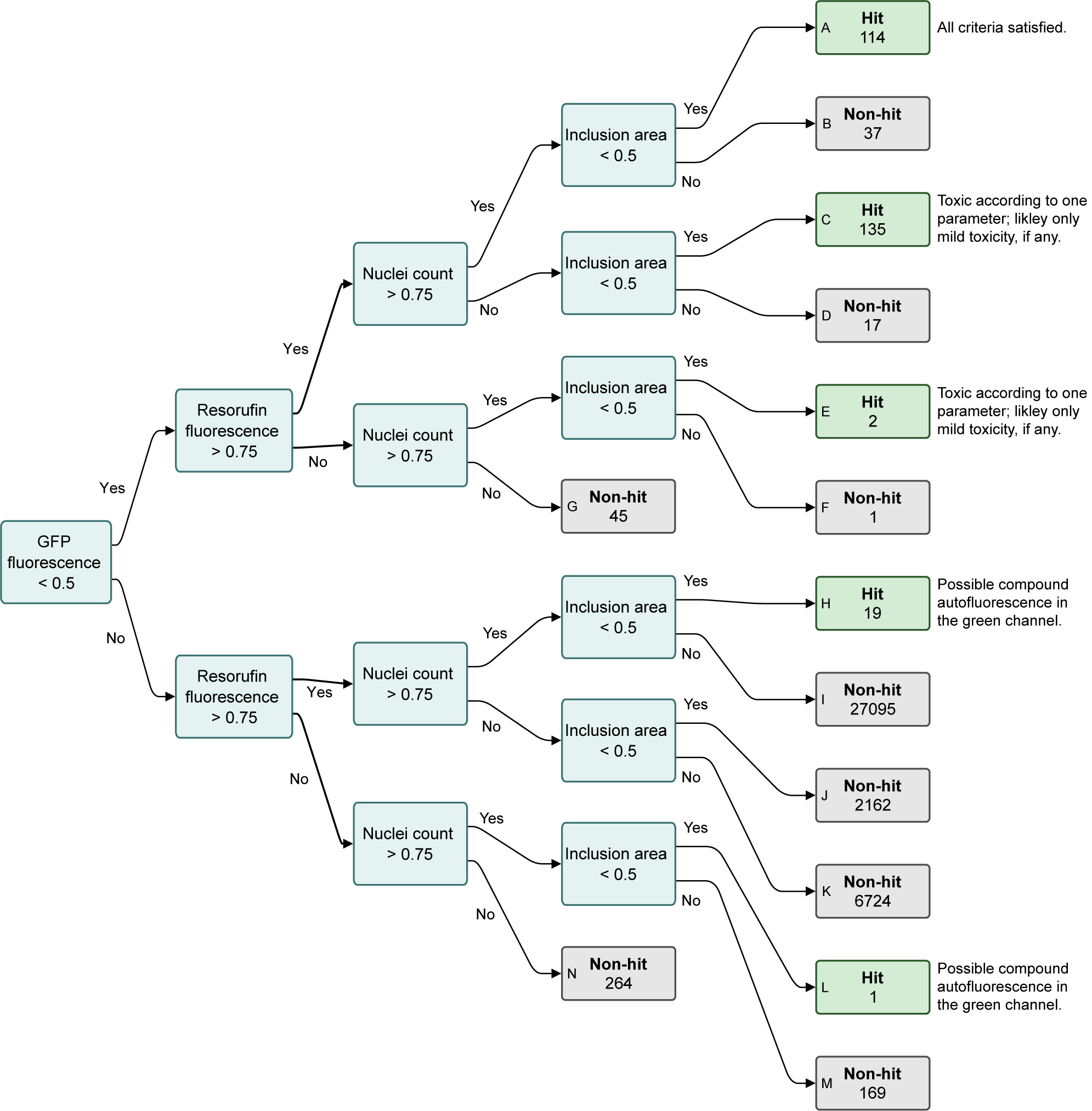

Supplement: S3 Fig — The decision tree enabled integration of information from both bulk fluorescence measurements and high-content imaging for hit selection. Inclusion area was selected as the parameter of choice for image-based assessment of bacterial growth, as all compounds that would have been hits according to inclusion count were also hits according to inclusion area, but not vice versa. (TIF) [file pbio.3003123.s003.tif]

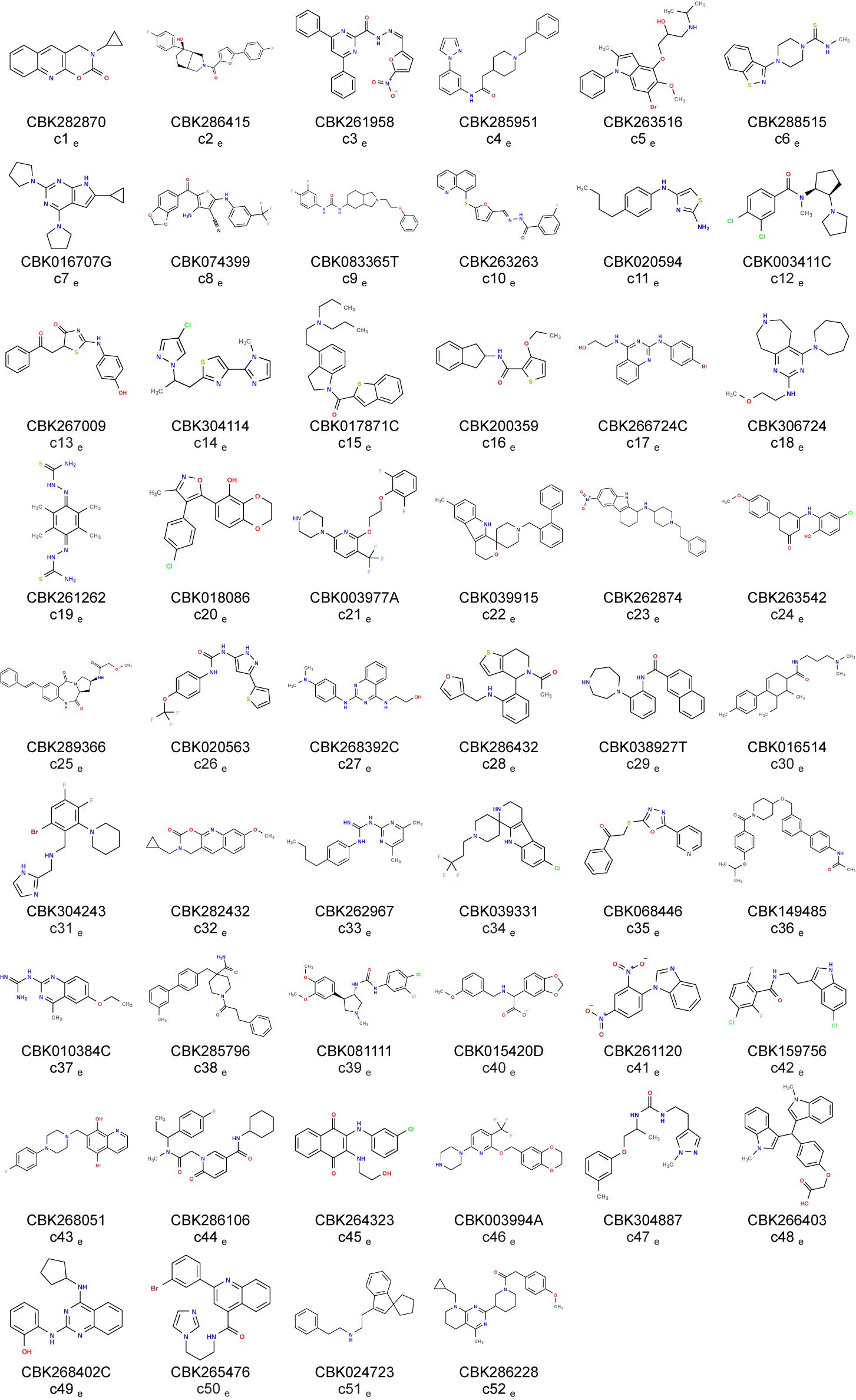

Supplement: S4 Fig — The structures were drawn based on their SMILES strings, using OpenBabel (version 3.0.0). (TIF) [file pbio.3003123.s004.tif]

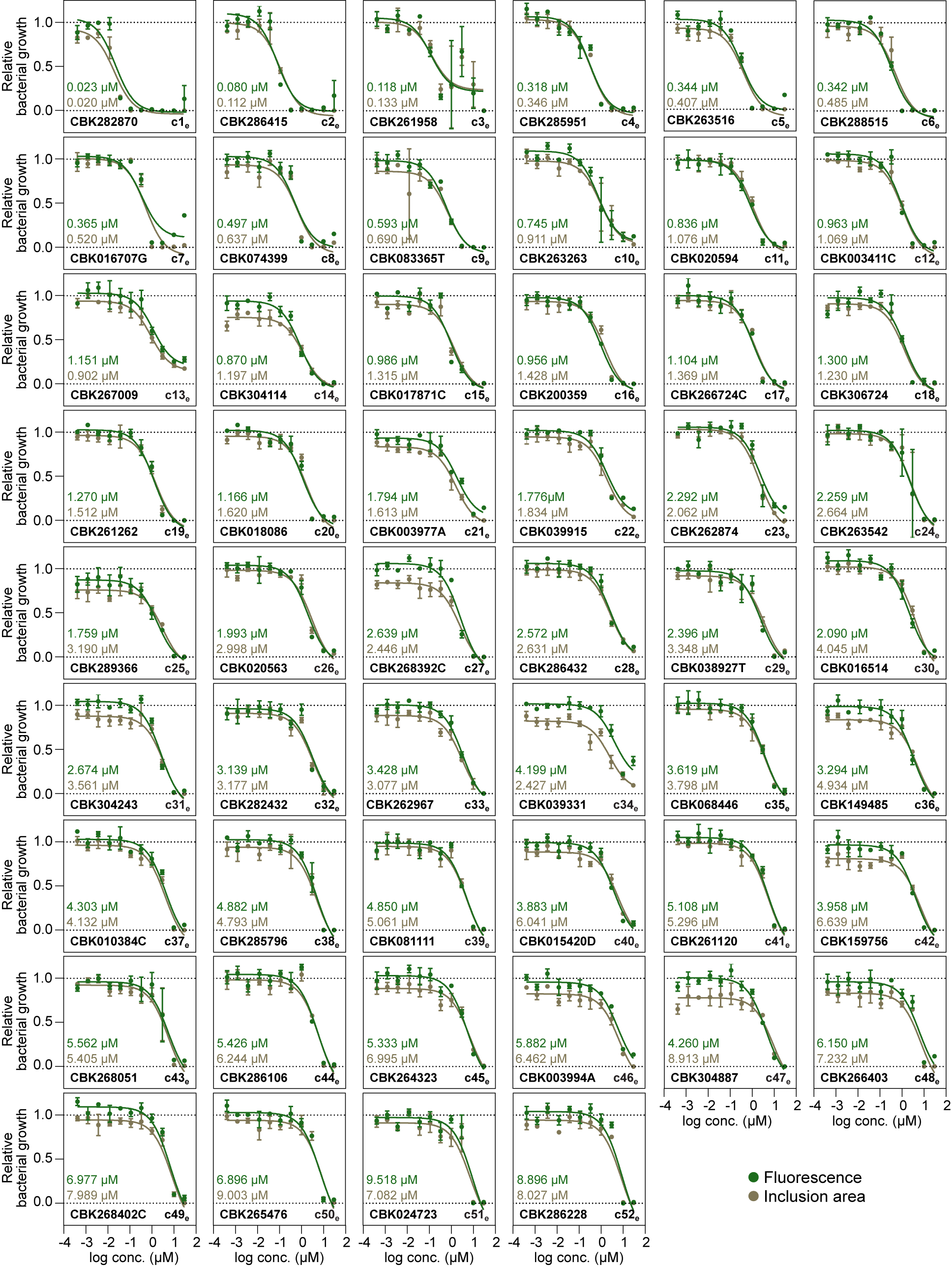

Supplement: S5 Fig — Data is shown for bulk GFP fluorescence as well as total inclusion area (mean ± SD, n = 3). The lines indicate the curve fits used for IC50 calculation, and the IC50 values are given in the respective plots. The data underlying this figure can be found in S2 Data. (TIF) [file pbio.3003123.s005.tif]

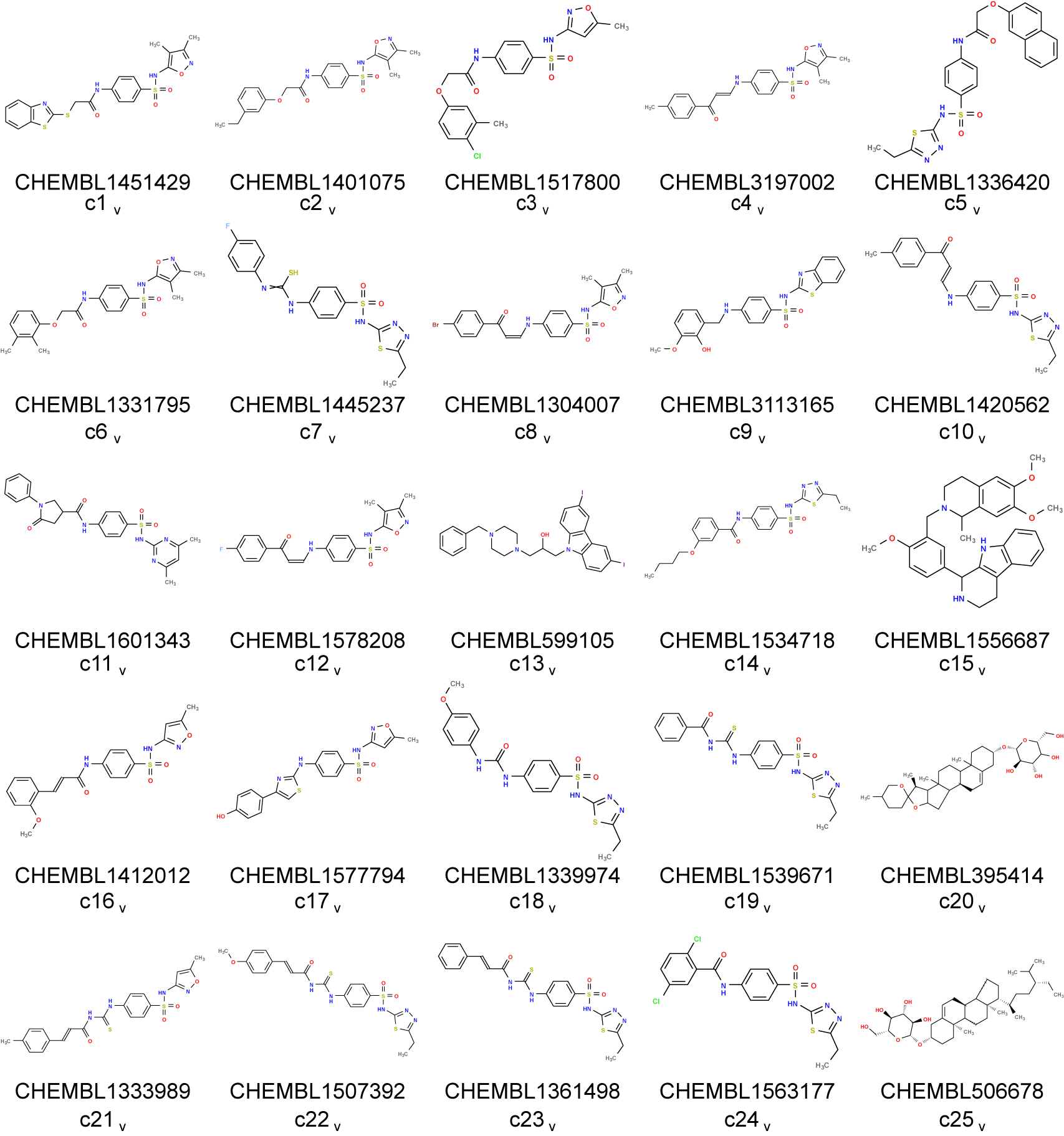

Supplement: S6 Fig — The structures were drawn based on their SMILES strings, using OpenBabel (version 3.0.0). Compounds c1v–c12v displayed antichlamydial activity with IC50 values below 10 µM and compounds c13v–c19v with IC50 values between 10 and 30 µM. (TIF) [file pbio.3003123.s006.tif]

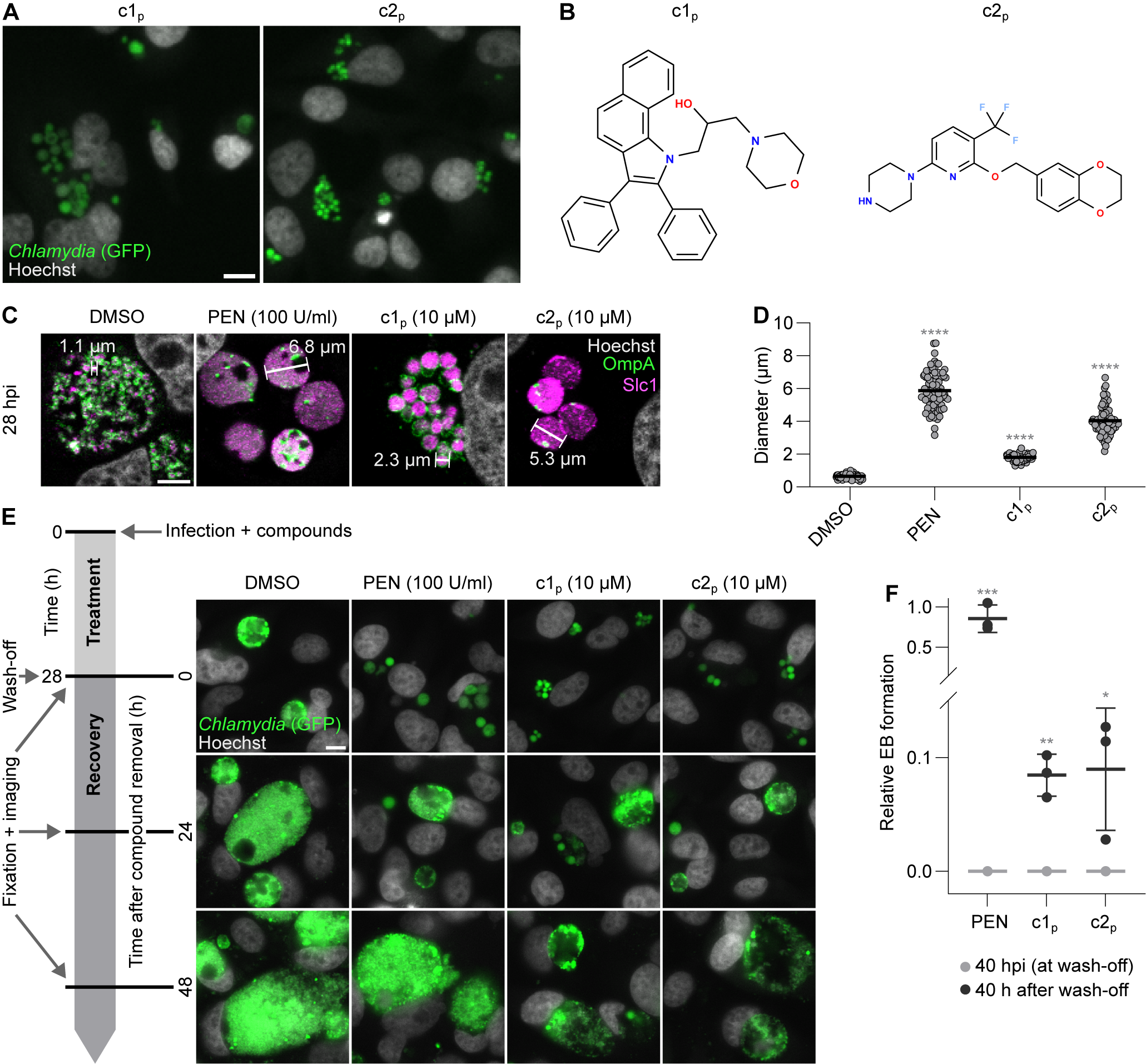

Supplement: S7 Fig — (A) Persistence-inducing compounds identified by inspection of images from the experimental compound library screen. Scale bar is 10 µm. (B) Structures of the identified compounds, drawn based on their SMILES strings, using OpenBabel (version 3.0.0). (C, D) Confocal microscopic confirmation of a persistent phenotype (formation of ABs) in HeLa cells infected with CTL2 and exposed to the indicated compounds. (C) Representative images of one of three experiments. Bacteria were detected using antibodies specific for OmpA (the major outer membrane protein) and Slc1 (a type III secretion chaperone). The annotated lines show the diameter of representative individual bacteria. Scale bar is 5 µm. (D) Size of compound-treated bacteria (collated data of n = 3, line at mean, 80 bacteria measured per condition and experiment, two-way ANOVA with Dunnett’s multiple comparisons test). (E) Recovery of the growth of CTL2-GFP in HeLa cells after compound removal. The images are representative of three experiments. Scale bar is 10 µm. (F) EB formation by CTL2-GFP and its recovery from persistence in HeLa cells after exposure to the compounds (10 µM) or penicillin G (100 U/ml) for 40 h (mean ± SD, n = 3). Data was normalized to values from DMSO-treated wells at wash-off (40 hpi), unpaired t tests with Holm-Sidak correction for multiple comparisons. PEN, penicillin G. The data underlying this figure can be found in S4 Data. (TIF) [file pbio.3003123.s007.tif]

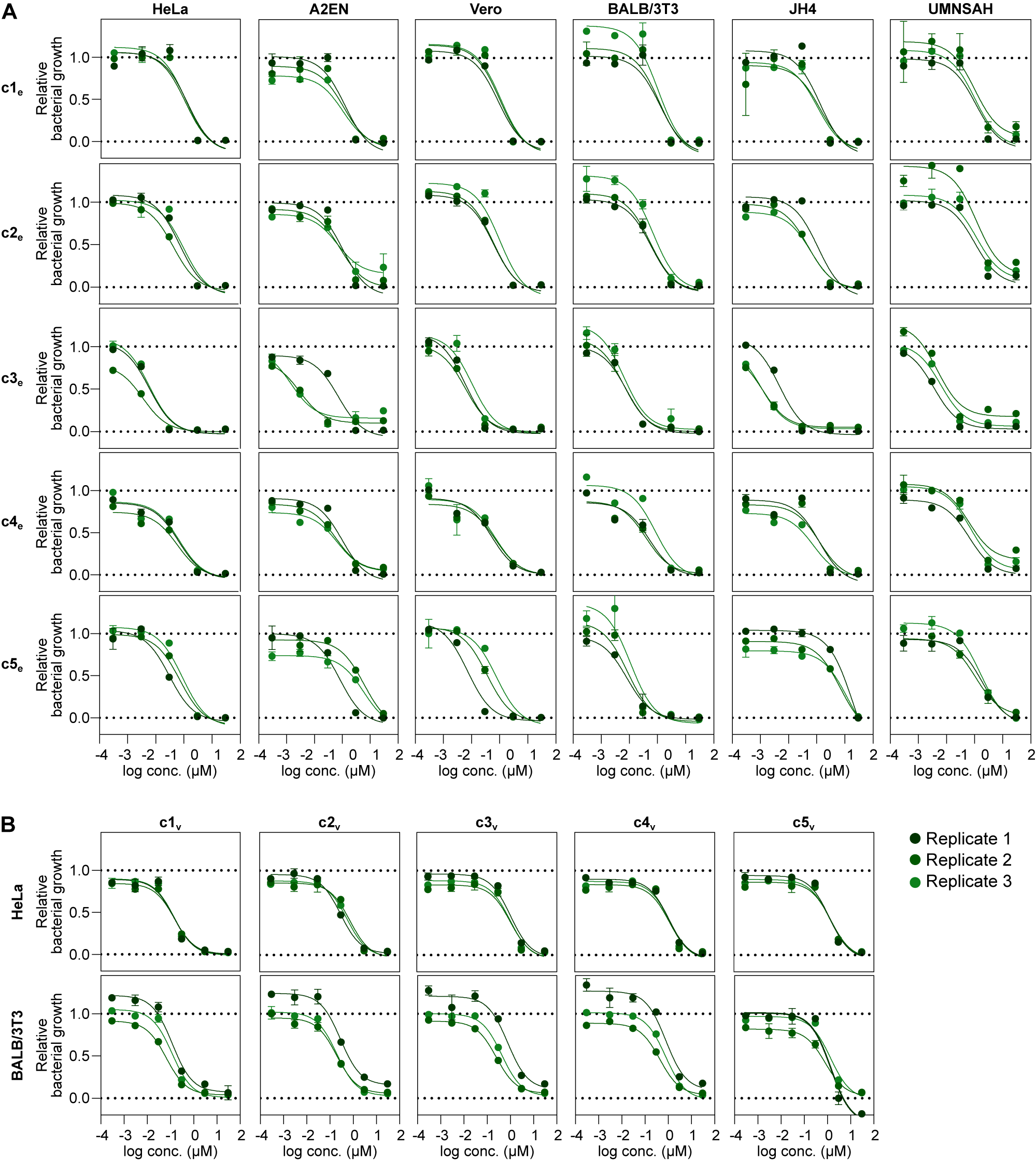

Supplement: S8 Fig — (A) Growth inhibition by top compounds from the experimental compound library screen. The data is based on measurements of bulk GFP fluorescence and shows separate curves from three biological replicates. Error bars indicate standard deviations of three technical replicates. The lines indicate the curve fits used for IC50 calculation. (B) Growth inhibition by top compounds from the virtual screen, presented as in (A). The data underlying this figure can be found in S5 Data. (TIF) [file pbio.3003123.s008.tif]

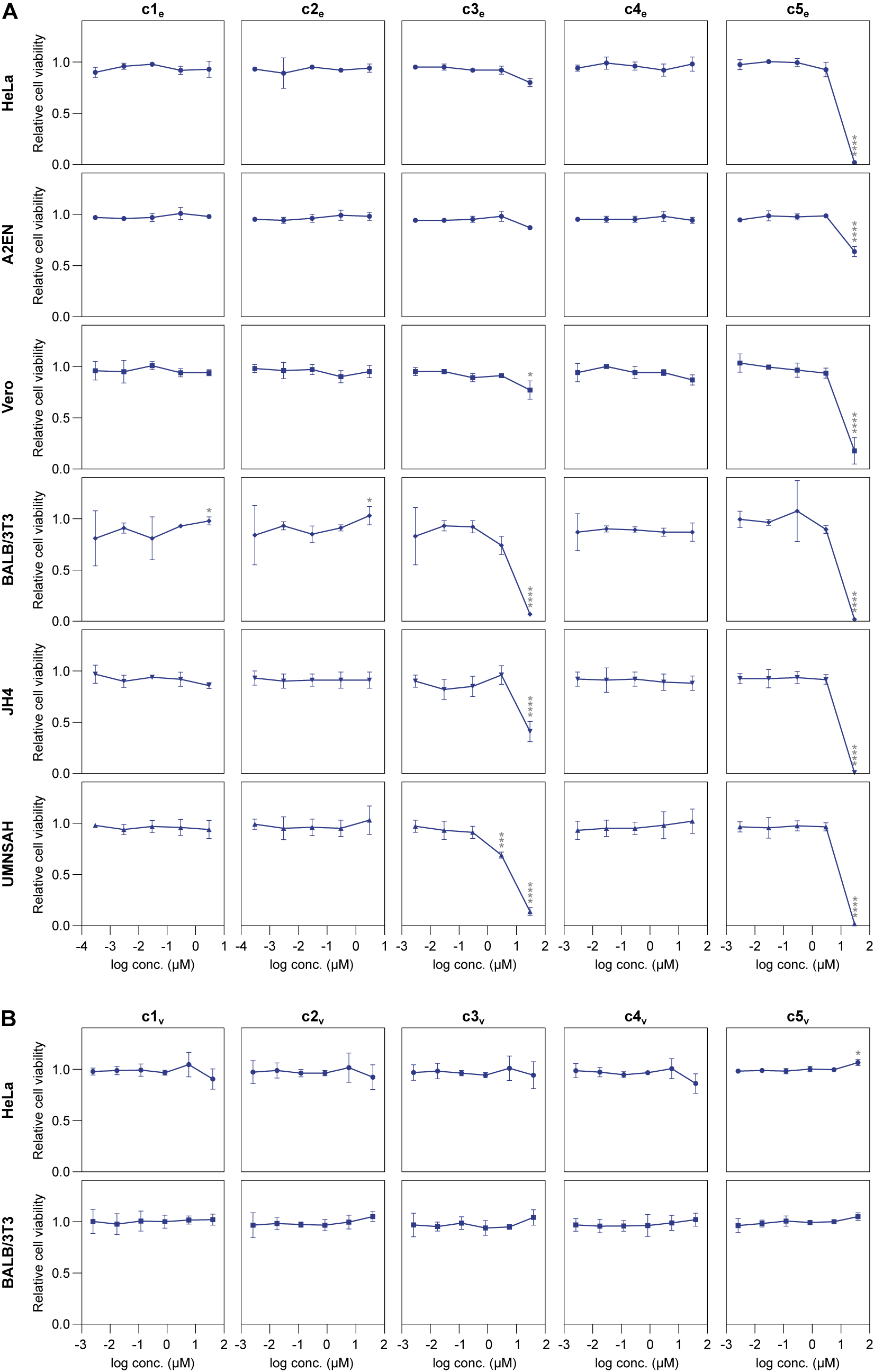

Supplement: S9 Fig — (A) Host cell viability after exposure to top compounds from the experimental compound library screen. The data is based on measurements of bulk resorufin fluorescence (mean ± SD, n = 3, two-way ANOVA with Dunnett’s multiple comparisons test of the lowest tested concentration of each compound vs. other concentrations). (B) Host cell viability after exposure to top compounds from the virtual screen, presented as in (A). The data underlying this figure can be found in S5 Data. (TIF) [file pbio.3003123.s009.tif]

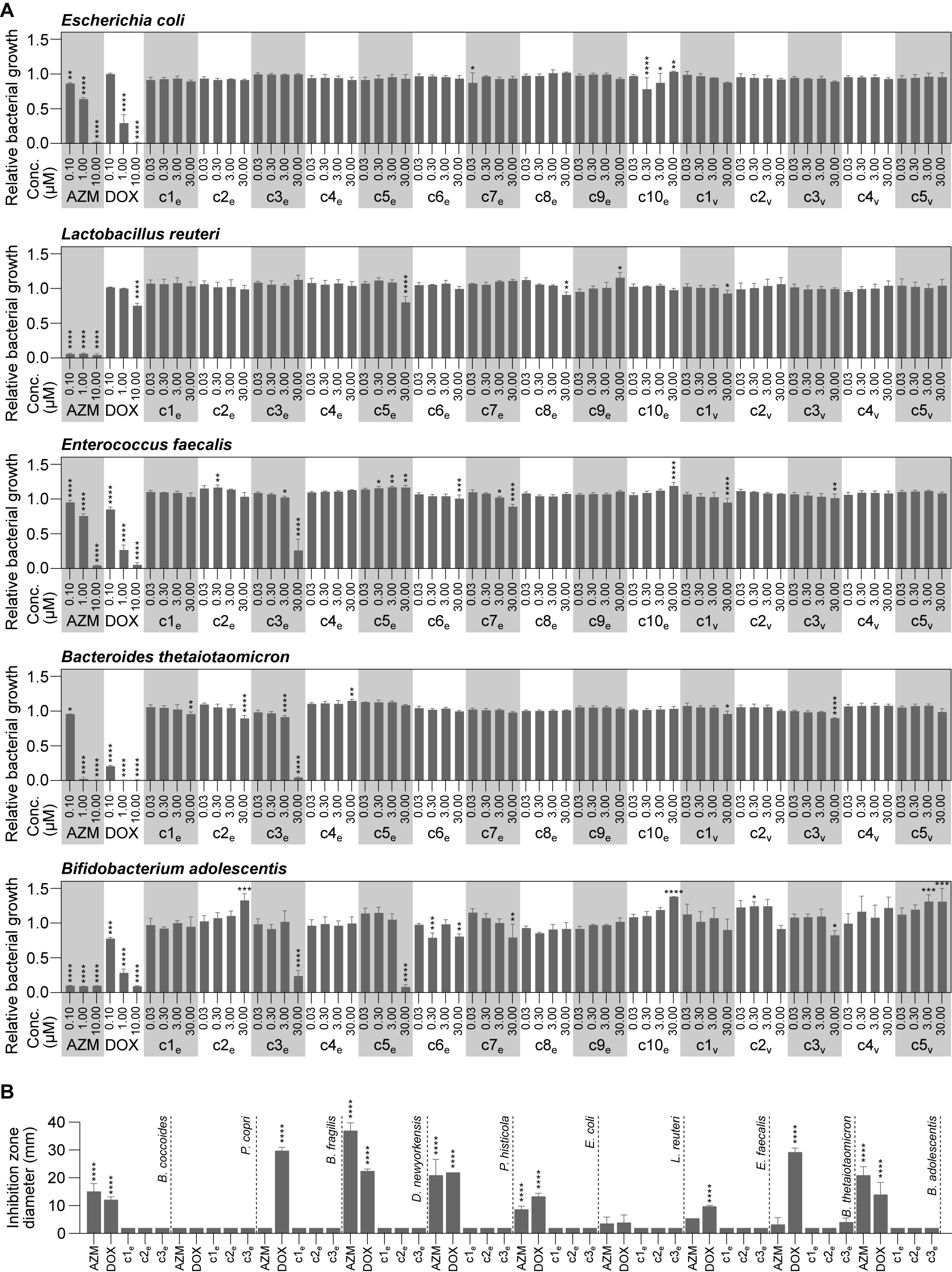

Supplement: S10 Fig — (A) Effect of top compounds on the growth of five species of gut bacteria in dilution assays. The bacteria were grown in liquid medium containing compounds for 18 h, at which point OD600 was measured. Data was normalized to values obtained from DMSO-treated wells (mean ± SD, n = 3, two-way ANOVA with Sidak’s multiple comparisons test of each compound and concentration vs. the mean of all 0.03 µM samples for a particular species). (B) Effect of c1e–c3e on the growth of additional gut bacteria species in radial diffusion assays (mean ± SD, n = 2–5, one-way ANOVA with Dunnett’s multiple comparisons test vs. non-inhibited DMSO-treated controls). Please note that the radial diffusion assay has limitations. It primarily produces qualitative results by indicating the presence or absence of antimicrobial activity and the diameters of inhibition zones can be influenced by various factors, including solubility and diffusion rate of the compounds. However, the more quantitative dilution assay was not applicable to all tested species, as for some species we encountered inconsistent bacterial growth in this format. AZM, azithromycin; DOX, doxycycline. The data underlying this figure can be found in S5 Data. (TIF) [file pbio.3003123.s010.tif]

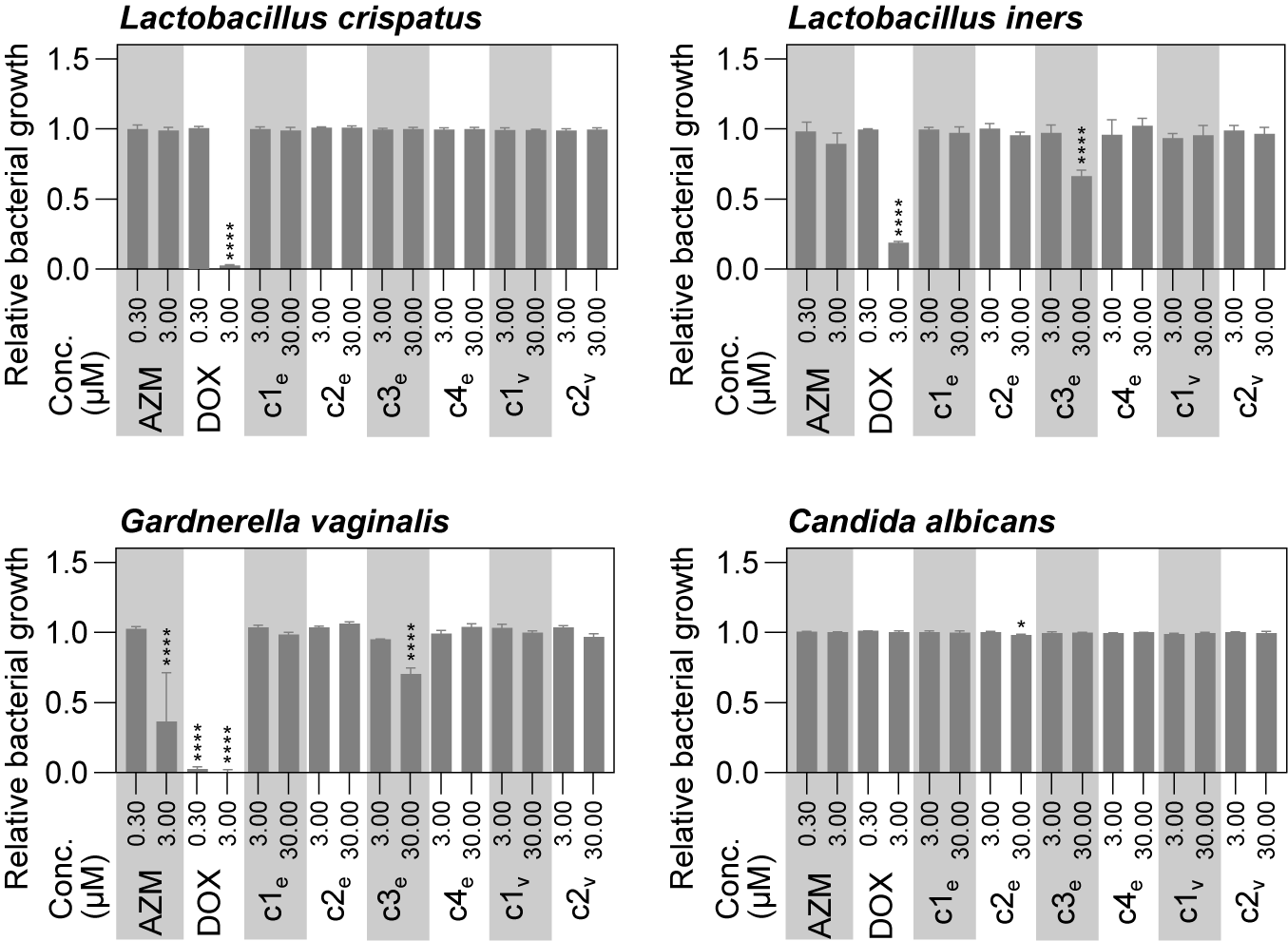

Supplement: S11 Fig — The microbes were grown in liquid medium containing compounds for 18–20 h, at which point OD600 was measured. Data was normalized to values obtained from DMSO-treated wells (mean ± SD, n = 3, two-way ANOVA with Sidak’s multiple comparisons test of each compound and concentration vs. the mean of all 3 µM samples for a particular species). AZM, azithromycin; DOX, doxycycline. The data underlying this figure can be found in S5 Data. (TIF) [file pbio.3003123.s011.tif]

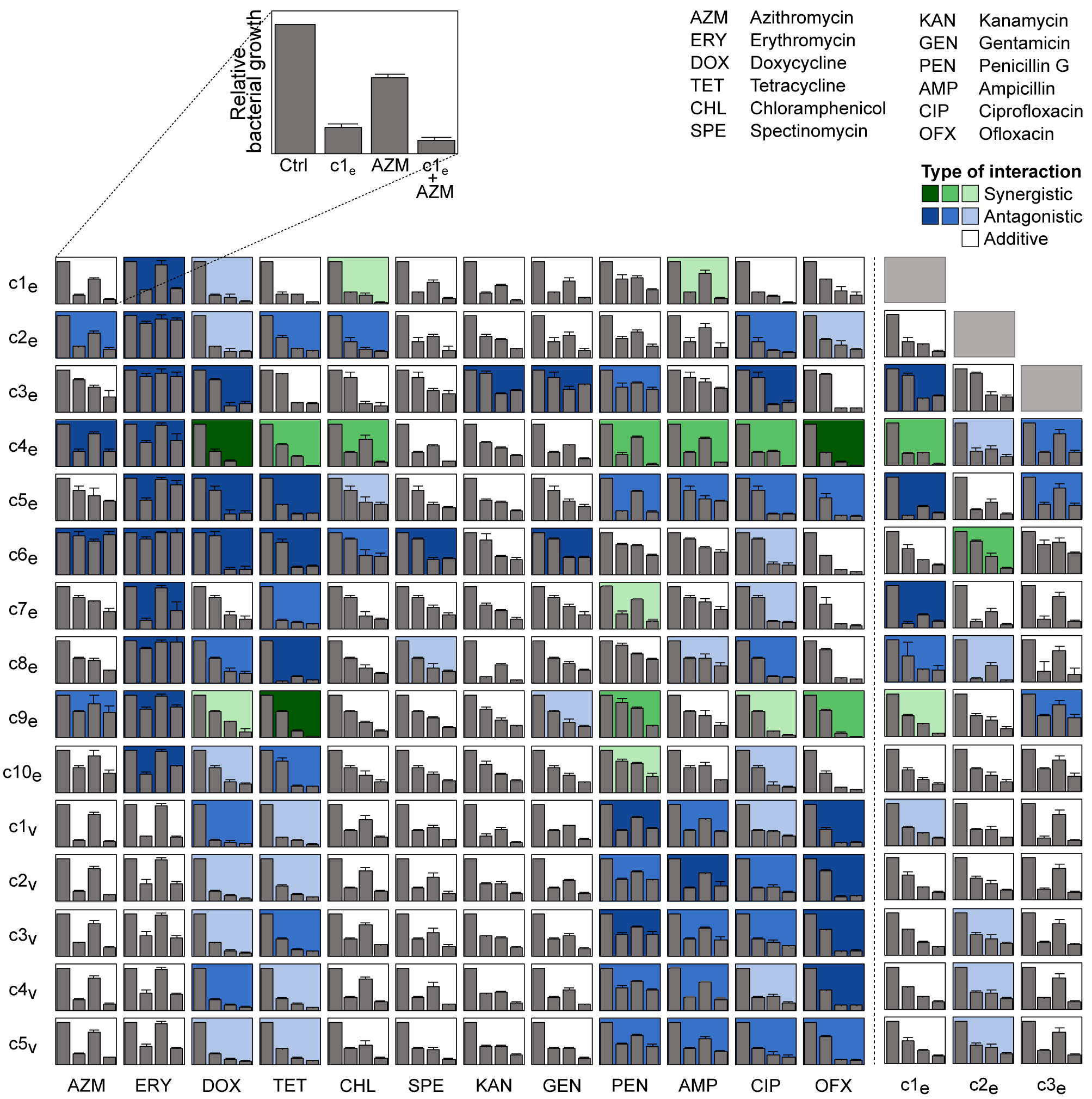

Supplement: S12 Fig — Bacterial growth inhibition in HeLa cells infected with CTL2-GFP (30 IFU/cell, 28.5 hpi) and classification of interaction type for all pairwise combinations of 15 selected top compounds with 12 clinical antibiotics (and c1e–c3e), tested at IC50 (mean ± SD, n = 3). Interaction type was classified based on calculations of epistasis, as described in the methods. Darker colors indicate stronger synergistic (green) or antagonistic (blue) interactions. Data for c1e–c5e were also included in Fig 4B. The data underlying this figure can be found in S6 Data. (TIF) [file pbio.3003123.s012.tif]

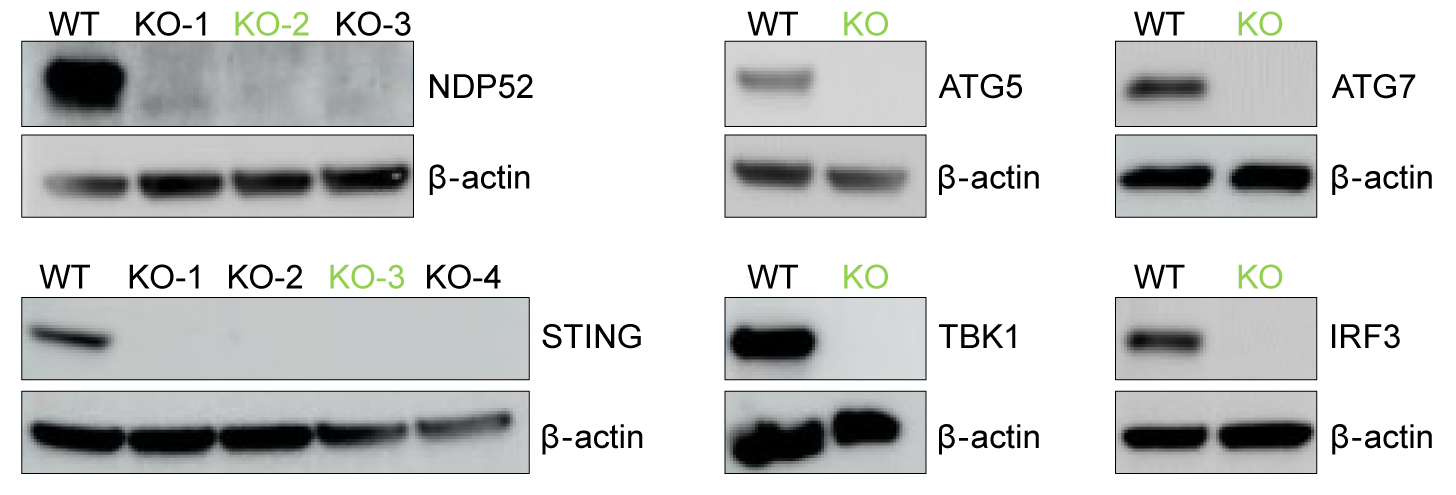

Supplement: S13 Fig — Western blot analysis of A2EN cell lines (wild-type (WT) or knockout (KO) for indicated genes) to confirm the absence of the targeted proteins in autophagy or the STING-pathway of the type I IFN response. Marked in green are the KO cell clones used in this study. The raw images of the blots are shown in S1 Raw Images. (TIF) [file pbio.3003123.s013.tif]

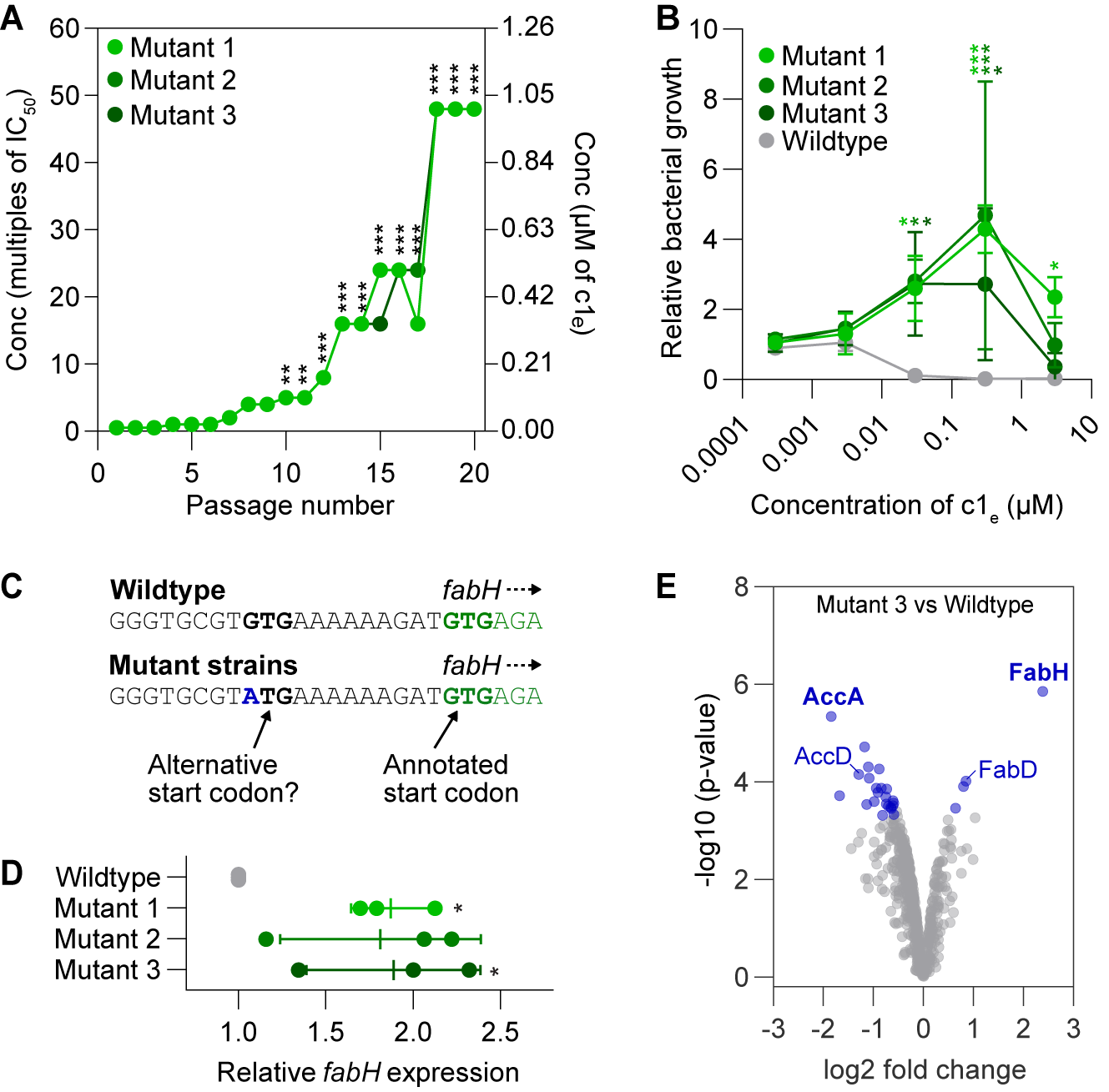

Supplement: S14 Fig — (A) Increase in the concentrations of c1e used during parallel passaging of three CTL2 strains evolving resistance (two-way ANOVA with Dunnett’s multiple comparisons test vs. passage 1). The starting concentration was 10.5 nM (0.5× IC50). Please note that the generation of resistant mutants was conducted solely for the purpose of target identification, not to estimate rates of resistance development. (B) Growth of the three evolved CTL2 strains in HeLa cells treated with the indicated concentrations of c1e, as measured by inclusion area. Bacterial growth is displayed relative to the DMSO control of the respective strain (mean ± SD, n = 3, two-way ANOVA with Dunnett’s multiple comparisons test vs. Wildtype). (C) Scheme illustrating the location of the mutation upstream of fabH (modified nucleotide marked in blue). (D) Expression levels of fabH in the three evolved CTL2 strains during infection of HeLa cells, as measured at 46 hpi by quantitative reverse transcription PCR (qRT-PCR) (mean ± SD, n = 3, one-way ANOVA with Dunnett’s multiple comparisons test vs. Wildtype). (E) Volcano plot displaying bacterial proteins up- or downregulated during infection of HeLa cells with the c1e-resistant CTL2 mutant 3 compared to infection with wild-type CTL2. Displayed are only data from the analysis conducted in the absence of c1e. The full dataset is available in S9F Data. The data underlying this figure can be found in S9 Data. (TIF) [file pbio.3003123.s014.tif]

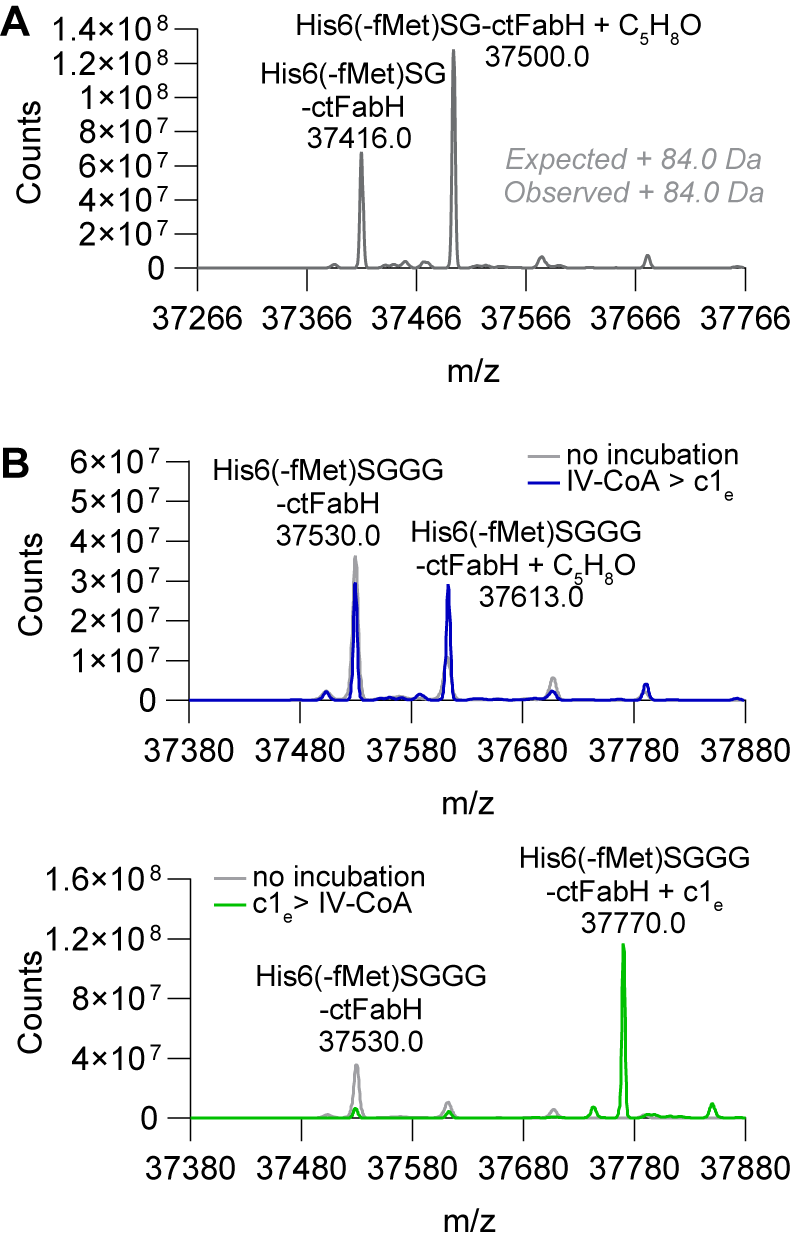

Supplement: S15 Fig — (A) Intact protein mass spectrometry of recombinant ctFabH after purification from Escherichia coli, demonstrating a large proportion of the protein to be modified by acyl groups, primarily pentanoyl-groups. (B) Intact protein mass spectrometry of purified acyl-modification-free ctFabH (10 µM) after sequential 10-min incubations with isovaleryl-CoA (IV-CoA, 50 µM) and c1e (50 µM) in the indicated (>) order. Note that prior incubation with isovaleryl-CoA or c1e prevents interaction with the respective other molecule during the second incubation step. The expected mass difference for +isovaleryl is +84.0 Da and for +c1e is +240.0 Da. The data underlying this figure can be found in S9 Data. (TIF) [file pbio.3003123.s015.tif]

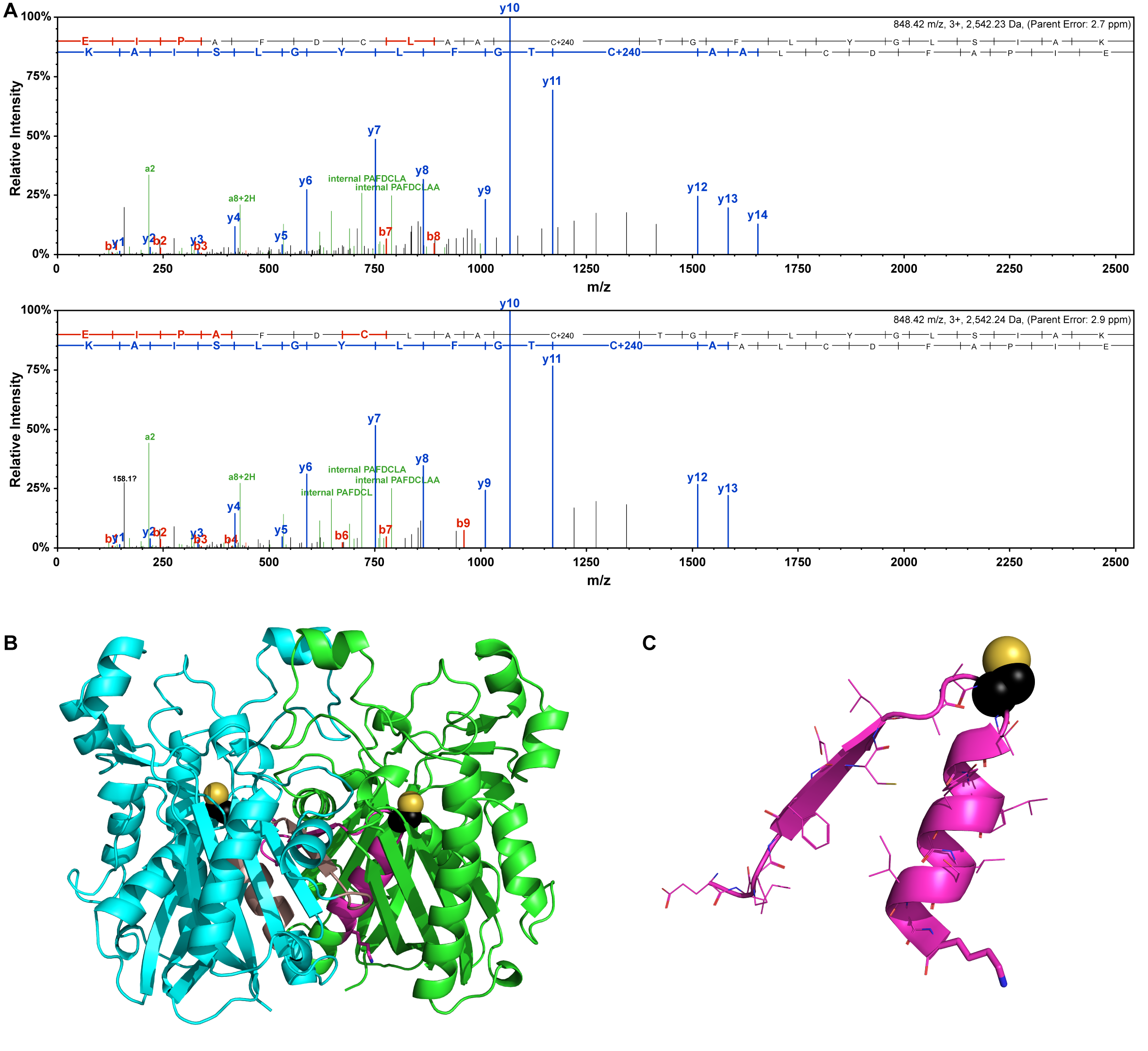

Supplement: S16 Fig — (A) Two representative MS/MS fragmentation spectra for a sample containing 10 µM ctFabH and 50 µM c1e. The predicted y- and b-ions are shown along the top of the figures and the fragments found in the spectra are marked in bold. The c1e-cysteine adduct is represented by the C+240 label. (B, C) AlphaFold3 model of a ctFabH dimer with the active site cysteine shown as spheres (black for carbon and gold for sulfur) and the active site peptide observed by mass spectrometry shown in magenta (B). A close-up of the active site peptide (C). (TIF) [file pbio.3003123.s016.tif]
