# Supplementary material for: A multi-strategy antimicrobial discovery approach reveals new ways to treat Chlamydia
Source: PLoS Biol. 2025 Apr 29;23(4):e3003123. doi: 10.1371/journal.pbio.3003123 (PMC12040169; doi:10.1371/journal.pbio.3003123)

S1 Raw Images

Part of Fig S13

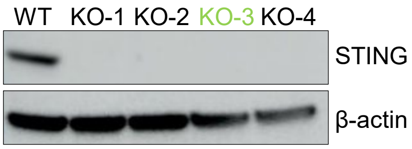

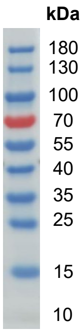

Original images

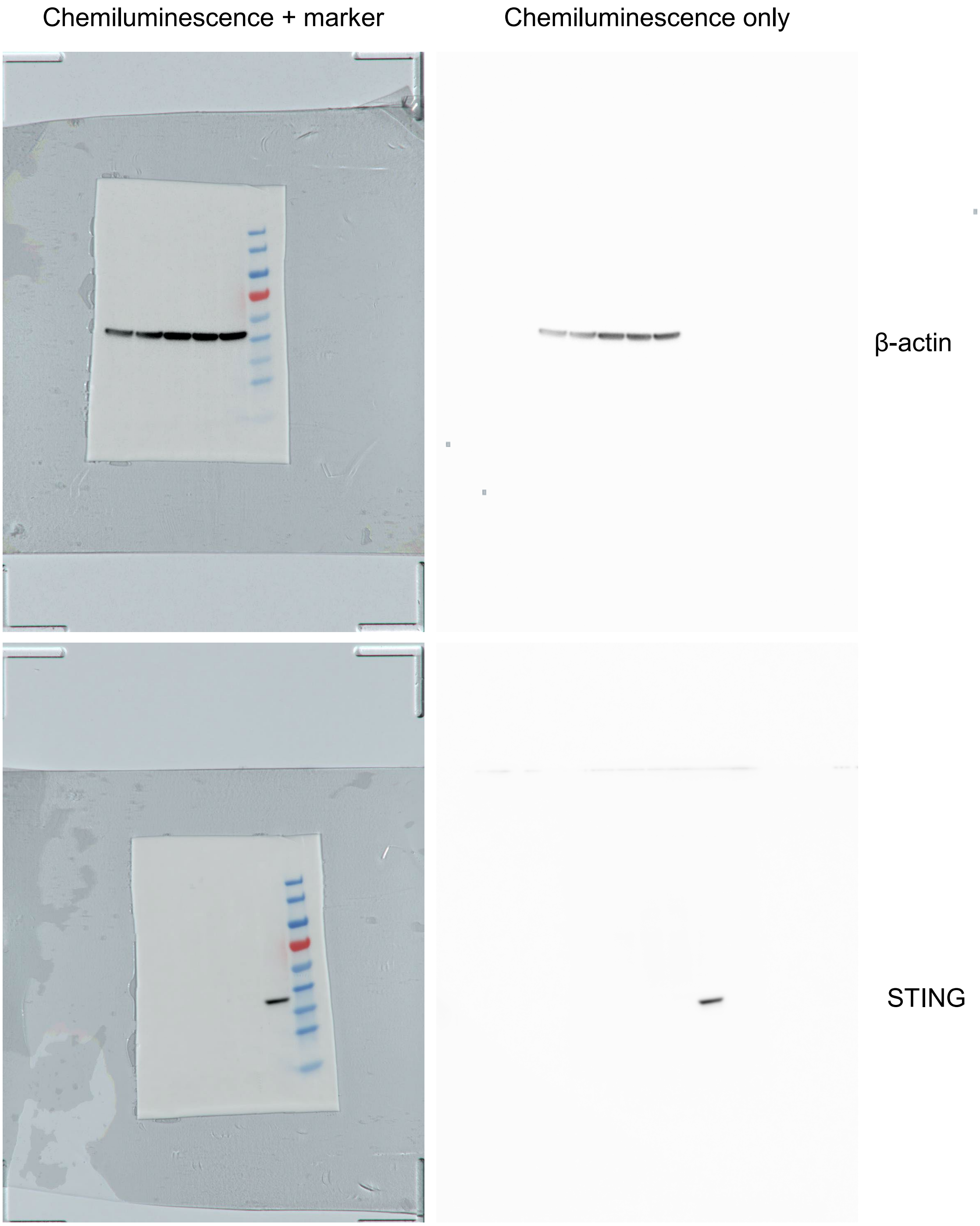

Note that to generate S13 Fig these images were flipped to show WT at the left side.

Part of Fig S13

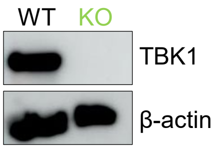

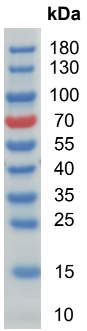

Original images

Chemiluminescence + marker   Chemiluminescence only

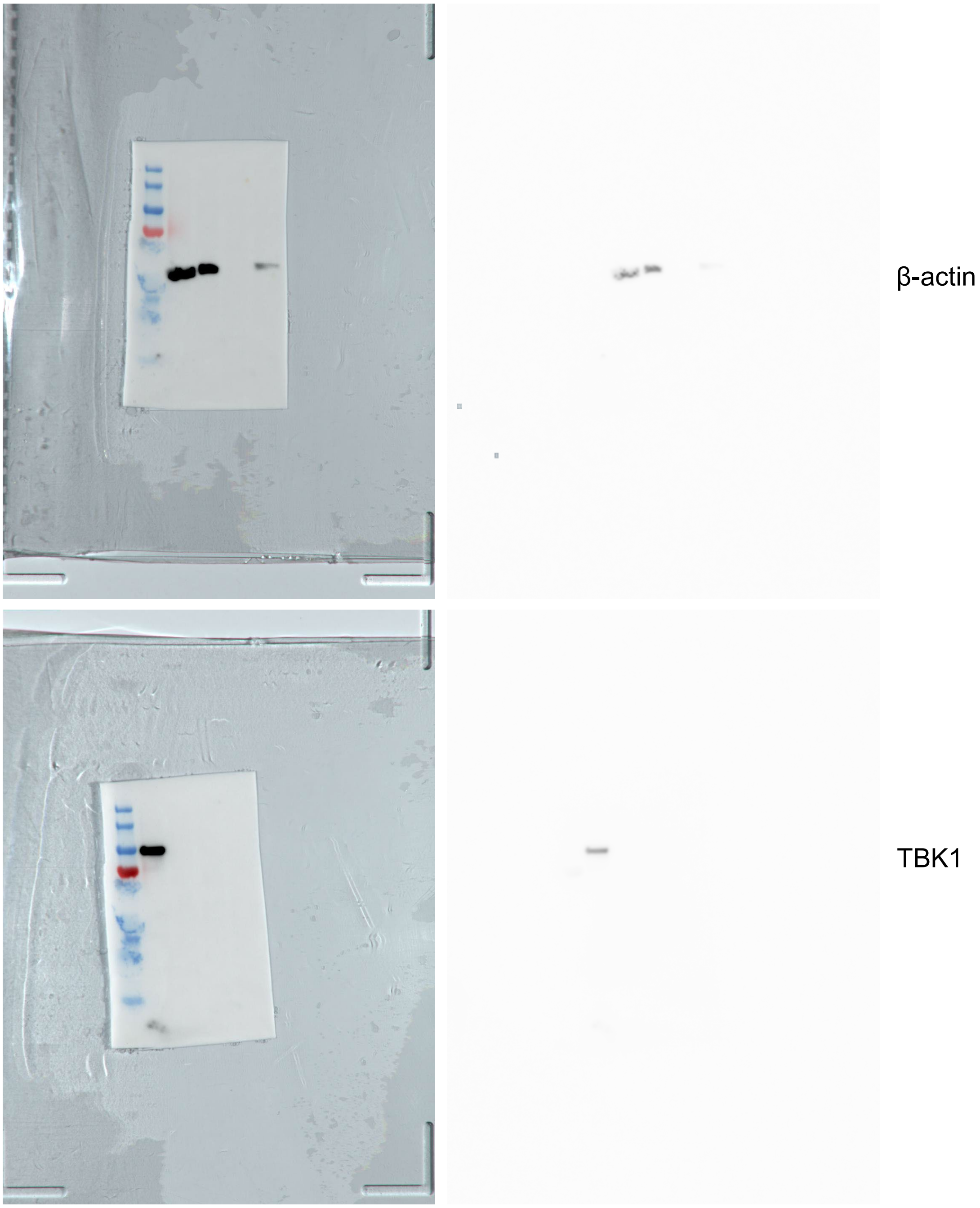

Part of Fig S13

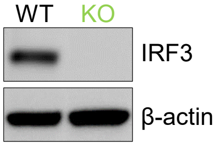

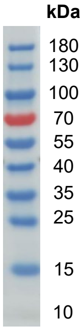

Original images

Chemiluminescence + marker

Chemiluminescence only

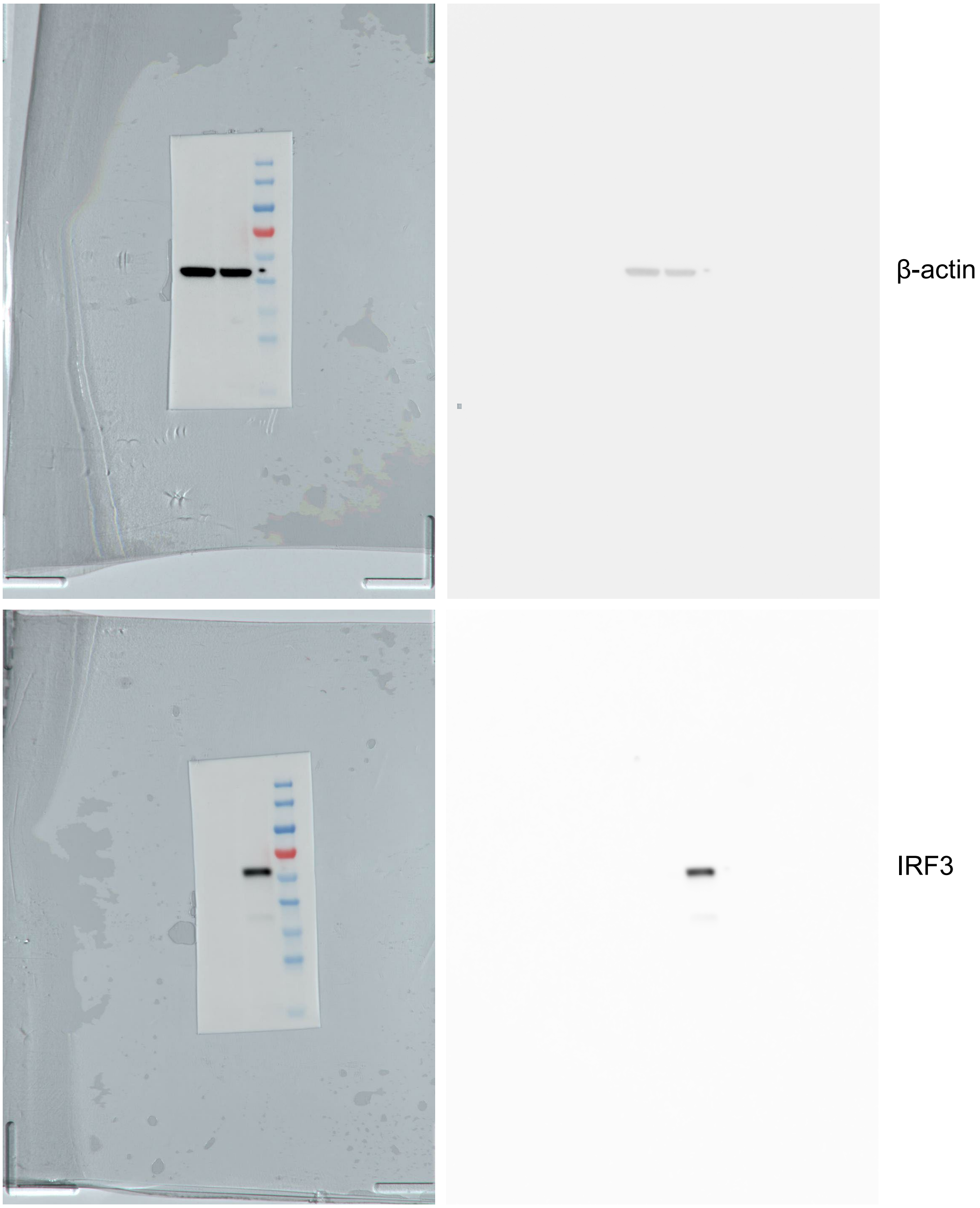

Note that to generate S13 Fig these images were flipped to show WT at the left side.

Part of Fig S13

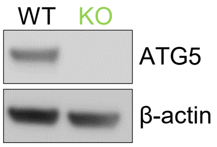

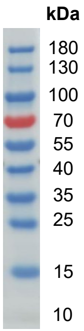

Original images

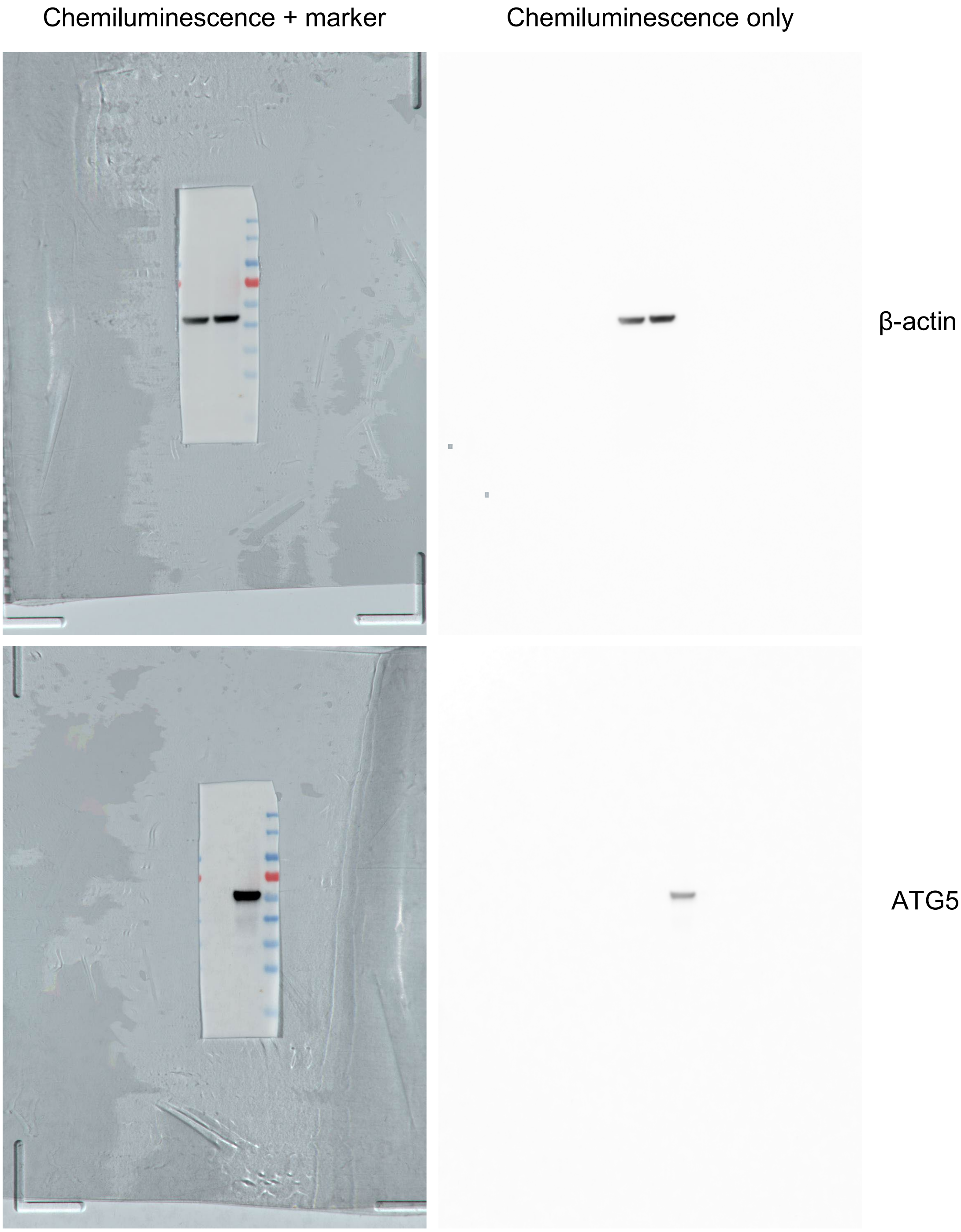

Note that to generate S13 Fig these images were flipped to show WT at the left side.

Part of Fig S13

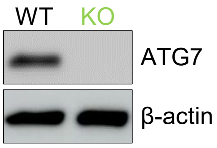

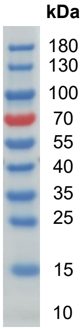

Original images

Chemiluminescence + marker | Chemiluminescence only

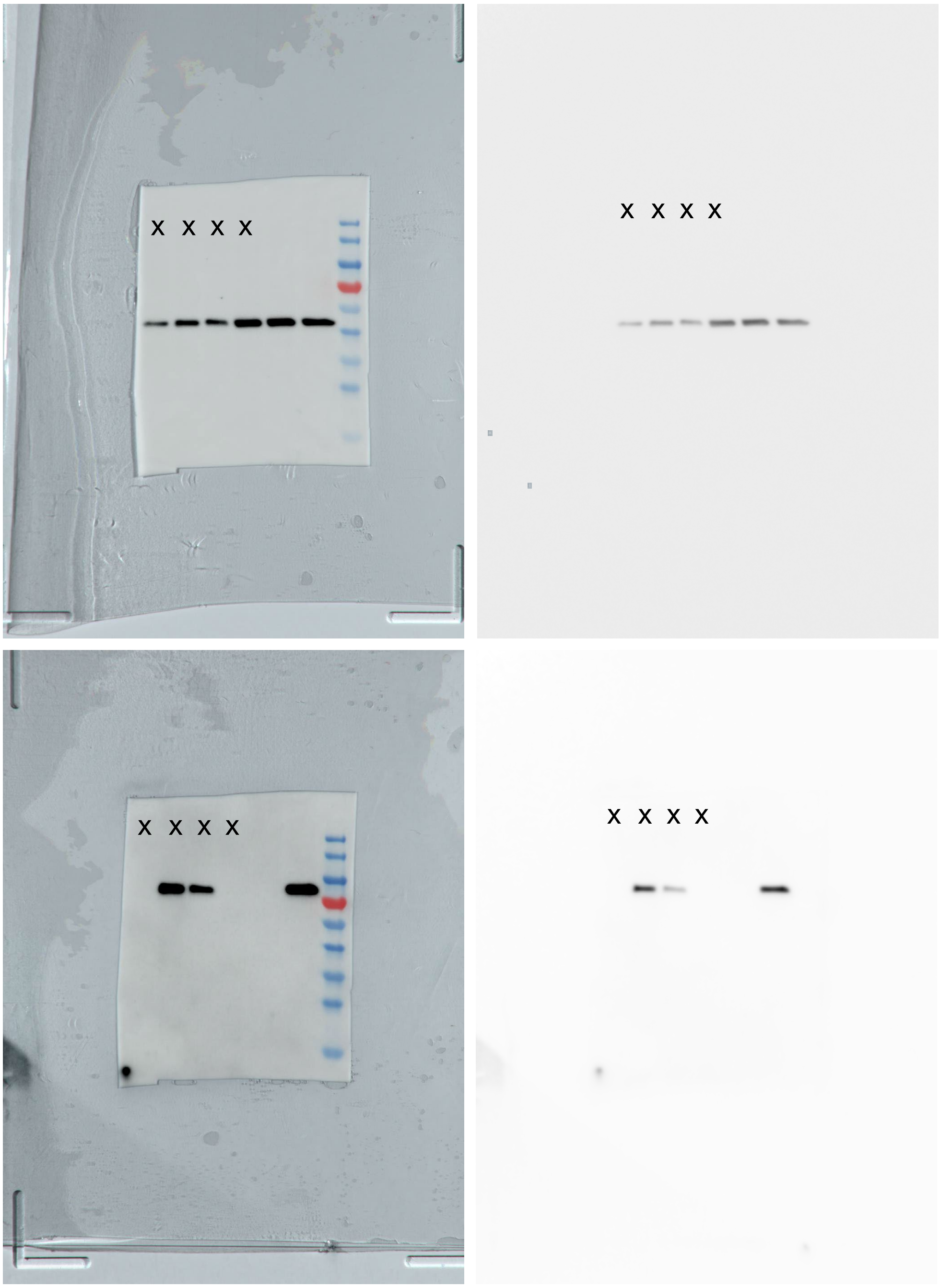

β-actin

ATG7

Note that to generate S13 Fig these images were flipped to show WT at the left side. Moreover, only the first two lanes are shown in Fig S13 as the other lanes (marked with x) in part contain clones that are not deficient for ATG7.

Part of Fig S13

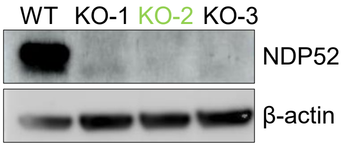

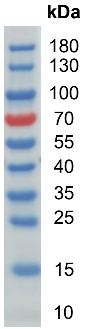

Original images

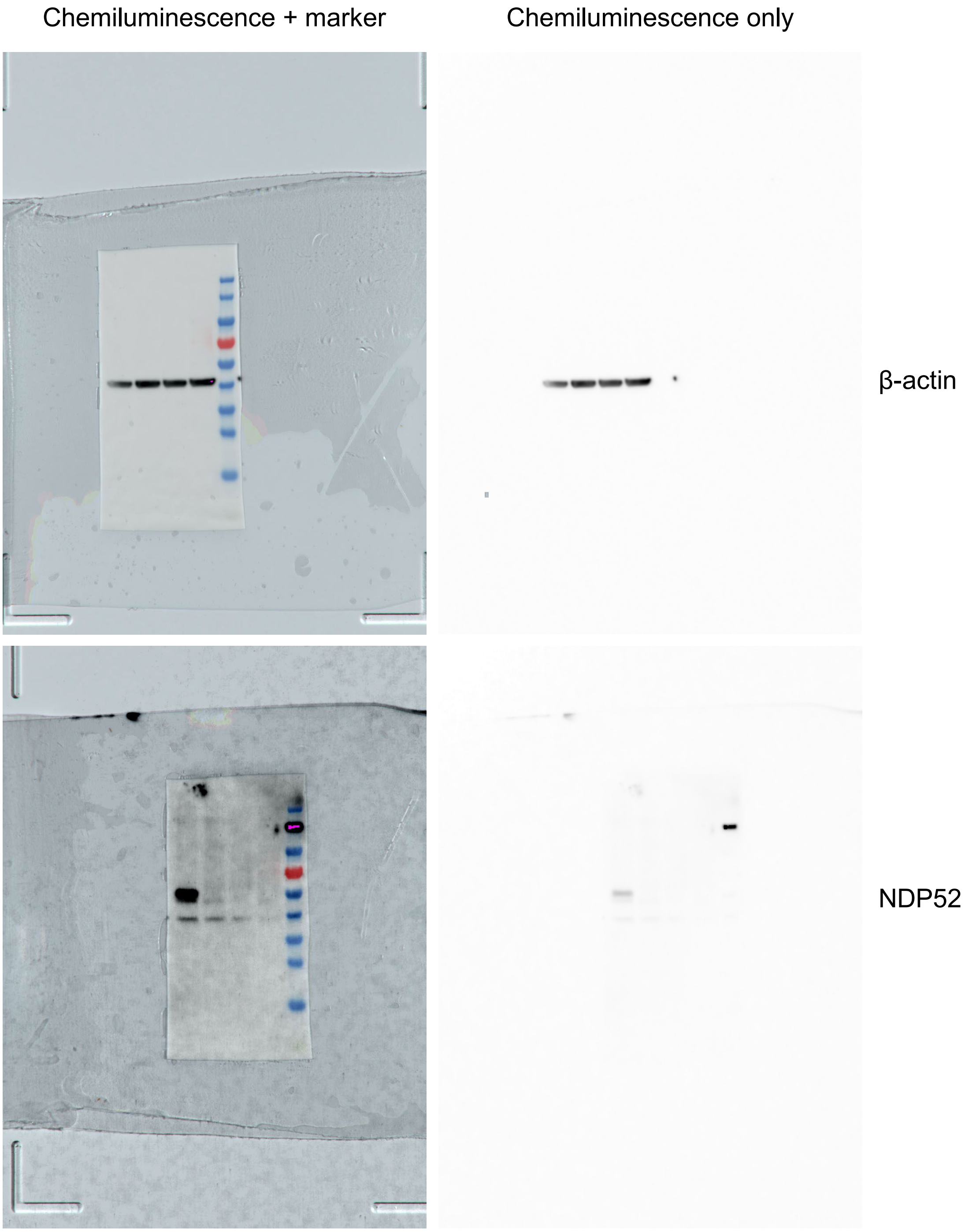

Supplement: S1 File — Raw images of western blots shown in S13 Fig. (PDF) [file pbio.3003123.s026.pdf]
